# Acute effects of daylight saving time clock changes on mental and physical health in England: population based retrospective cohort study

**DOI:** 10.1136/bmj-2025-085962

**Published:** 2025-12-17

**Authors:** Melanie A de Lange, Kate Birnie, Rebecca C Richmond, Chin Yang Shapland, Sophie V Eastwood, Kate Tilling, Neil M Davies

**Affiliations:** 1 MRC Integrative Epidemiology Unit, Bristol Medical School, University of Bristol, Bristol, UK; 2 Population Health Sciences, Bristol Medical School, University of Bristol, Bristol, UK; 3 NIHR Oxford Health Biomedical Research Centre, University of Oxford, Oxford, UK; 4 Institute of Cardiovascular Science, University College London, London, UK; 5 Division of Psychiatry, University College London, London, UK; 6 Department of Statistical Science, University College London, London, UK; 7 Department of Public Health and Nursing, Norwegian University of Science and Technology, Trondheim, Norway

## Abstract

**Objective:**

To explore the acute effects of daylight saving time clock changes on mental and physical health events in primary and secondary care in England.

**Design:**

Population based retrospective cohort study.

**Setting:**

English primary care practices contributing to the Clinical Practice Research Datalink GOLD database, linked to hospital admissions and accident and emergency data.

**Participants:**

683 809 people (road traffic injuries: all ages; cardiovascular disease: aged ≥40 years; all other conditions: ≥10 years) registered with a participating English general practice, with a health event for one of the health conditions of interest in their primary or secondary care record in the eight weeks surrounding the spring or autumn clock changes between 2008 and 2019.

**Main outcome measures:**

Health events were defined as a diagnosis code (or symptom code and prescription for mental health conditions in primary care) of anxiety, major acute cardiovascular disease, depression, eating disorder, road traffic injury, self-harm, or sleep disorder in primary or secondary care or a psychiatric condition in accident and emergency. Negative binomial regression models, adjusted for day of the week and region (and Easter weekend in spring), compared mean event rates per day in the week after the clock changes and the control period (four weeks before the changes and weeks 2-4 after).

**Results:**

In the week after the autumn clock change, five health conditions had fewer events: anxiety (from 17.3 events per day (per year, per region) to 16.7; incidence rate ratio 0.97, 95% confidence interval 0.95 to 0.98), acute cardiovascular disease (from 50.0 to 48.9; 0.98, 0.96 to 0.999), depression (from 44.6 to 42.7; 0.96, 0.95 to 0.97), psychiatric conditions (from 3.5 to 3.3; 0.94, 0.90 to 0.98), and sleep disorders (from 5.4 to 4.9; 0.92, 0.87 to 0.97). Little evidence was found of reductions in eating disorder diagnoses, road traffic injuries, or self-harm or of changes after the spring clock change.

**Conclusion:**

The week after the autumn clock change was associated with a reduction in events for cardiovascular disease, sleep disorders, and mental health disorders, but little evidence suggested that the spring clock change was associated with a change in the number of health events. Electronic health records contain the date that a health event is recorded by a clinician, which is not necessarily the date of symptom onset.

## Introduction

Daylight saving time was introduced during the first world war and involves moving the clocks one hour forward in spring and one hour back in autumn. Daylight saving time operates in around 70 countries and affects a quarter of the world’s population.^1^ ^2^ However, in recent years the clock changes have become the focus of intense debate.

Research conducted outside of the UK suggests that the clock changes may be detrimental to population health, via changes in light exposure and disruption to sleep and circadian rhythms.^3^ For example, a meta-analysis of 12 studies from 10 countries reported that the risk of acute myocardial infarction increased by 4% in the week after the spring clock change.^4^ Furthermore, a study using 20 years of US registry data identified a 6% rise in fatal traffic accidents in the week after the spring clock change,^5^ and an analysis of nationwide psychiatric hospitals in Denmark found an 11% rise in the incidence rate of unipolar depression after the autumn clock change.^6^ However, other studies have found little evidence that the clock changes increase the risk of adverse health events.^7^ ^8^ ^9^ ^10^ ^11^ ^12^ ^13^ ^14^

Although several UK studies have examined the effects of the clock changes on sleep,^15^ ^16^ ^17^ ^18^ research on the acute downstream effects of the clock changes on health in the UK has been limited. One study found that neither the spring nor the autumn clock change affected psychiatric admissions or suicides in Scotland.^12^ Another study, using UK survey data, reported a very small reduction in self-reported life satisfaction in the week after the spring clock change but no effect after the autumn clock change.^19^ Meanwhile, a few studies have looked at the effects of the daylight saving time transitions on UK road traffic accidents.^20^ ^21^ ^22^ In contrast to previous findings in the US,^5^ the most recent of these examined 14 years of police data from Great Britain and reported no effect on road traffic fatalities after the spring clock change. Furthermore, the study actually found a very small reduction in casualties after the spring clock change (0.003% fewer casualties per year). It detected little evidence of changes in casualties or fatalities after the autumn clock change.^11^

In the past decade, countries such as Mexico, Turkey, and Jordan have ended their twice yearly clock changes.^23^ ^24^ ^25^ In the US, several recent attempts (the latest in January 2025) have been made to introduce a “sunshine protection act” that would make daylight saving time permanent all year round. However, this is yet to make it into federal law.^26^ ^27^ ^28^ In 2019 the European Union voted to stop changing the clocks in EU countries in 2021.^29^ However, this policy has yet to be implemented, owing in part to the covid-19 pandemic, Brexit, and the complexity of agreeing on what system to change to.^30^ Nevertheless, October 2025 saw renewed interest in the plans among members of the European Parliament, with a debate about the barriers preventing the policy from moving forward.^31^ ^32^ The EU’s decision in 2019 did not prompt an immediate policy change in the UK. However, a subsequent House of Lords inquiry highlighted the need for more research.^33^ In late 2024 the British Sleep Society joined the appeal from other sleep societies for the daylight saving time clock changes to be abolished and recommended that permanent standard time (Greenwich Mean Time) be adhered to throughout the year.^34^ In March 2025, in a debate about the possible benefits of implementing double British Summer Time (having the clocks two hours ahead of GMT in summer and one hour ahead in winter), the UK government stated that the current evidence for altering the existing daylight saving time system was not overwhelming but raised some interesting questions, such as the effects of the clock changes on road safety and mental health.^35^

Here, we used a large database of linked primary and secondary care records to investigate the acute effects of the clock changes on people’s health in England. The health conditions we examined were anxiety, major acute cardiovascular disease, depression, eating disorder, road traffic injury, self-harm, and sleep disorders in primary or secondary care (hospital admissions and accident and emergency visits), as well as psychiatric conditions in accident and emergency. We hypothesised that the number of events for all of these health conditions would increase after the spring clock change, when people tend to lose sleep, and decrease after the autumn clock change, when they typically gain sleep.

## Methods

### Data sources

This research was pre-specified in Clinical Practice Research Datalink (CPRD) protocol 22/002468 (see supplementary text S1 for the protocol and S2 for minor deviations from the protocol). We used the CPRD GOLD database. This is a UK longitudinal primary care database established in 1987. It contains anonymised, routinely collected medical records from participating primary care practices using the Vision software system.^36^ The July 2024 version used in this study contained the records of more than 21.5 million people from 985 primary care practices. As of mid-2024, 4.3% of the UK population and 4.4% of UK primary care practices actively contributed to the database.^37^

CRPD GOLD is broadly representative of the UK population in terms of age, sex, and ethnicity compared with the UK census in 2011.^36^ Data include diagnoses, symptoms, prescriptions, tests, vaccinations, referrals, demographics, and lifestyle information.^36^ We also used linked data on hospital admissions from Hospital Episode Statistics Admitted Patient Care (HES APC), hospital accident and emergency visits (HES A&E), and Office for National Statistics (ONS) Index of Multiple Deprivation (IMD) (based on patient’s postcode). Because linked data were available only for 75% of English practices,^36^ our study was restricted to England.

### Study population

**Fig 1:**
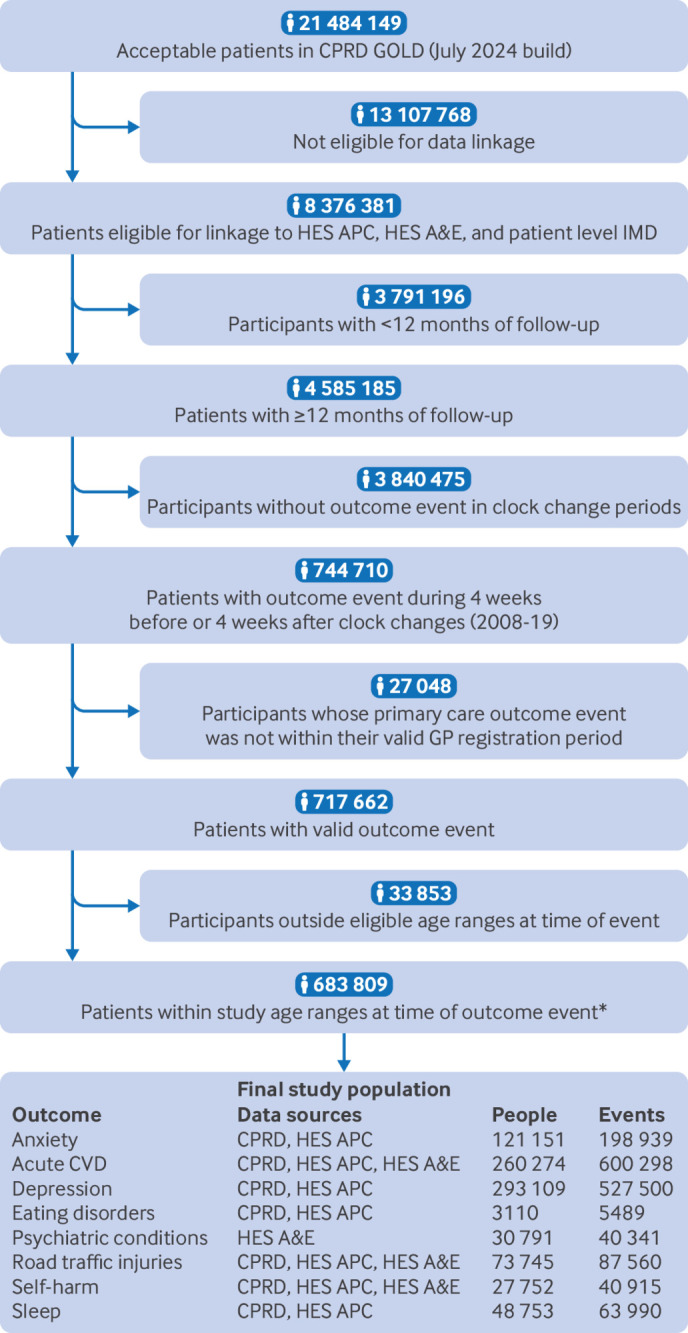
Participant flow diagram. *Road traffic injuries: all ages; cardiovascular disease (CVD): ≥40 years; other conditions: ≥10. CPRD=Clinical Practice Research Datalink; HES A&E=Hospital Episode Statistics accident and emergency visits; HES APC=Hospital Episode Statistics Admitted Patient Care

To be included in this population based retrospective cohort study, patients had to be registered at an English general practice, have a record classified as “acceptable” by CPRD, be registered at an “up to standard” general practice (in terms of quality of practice data), and be eligible for data linkage to HES APC, HES A&E, and patient level IMD. Patients also had to have at least one year of research quality follow-up time in CPRD GOLD during the study period (established by CPRD using practice and patient level data quality checks). We also required patients to have a health event (defined below) for one of our eight health conditions of interest in their primary or secondary care record within the eight weeks surrounding the spring or autumn clock changes between 2008 and 2019. Because this study looks at the numbers of events in particular eight week periods, and we can assume that the population remains fairly constant over these small periods of time, only people with events contributed to the analysis. For quality control purposes, we excluded primary care events outside of a patient’s valid general practice registration period (based on practice and patient level data quality checks). We placed no age restrictions on road traffic injury events. However, for acute cardiovascular disease events to be included, patients had to be aged ≥40 years at the time of the clock change (the age at which people become eligible for their NHS health check and are at higher risk of cardiovascular disease).^38^ For all other health conditions, patients had to be aged ≥10 years at the time of the clock change. We chose age ≥10 to ensure that we captured adolescents who may be disproportionately affected by the sleep deprivation caused by the clock changes owing to a shift towards having a later chronotype (going to bed and getting up later) during adolescence.^39^ ^40^ Patients could have events for more than one of our health conditions during the study period and could contribute to both spring and autumn analyses. Our final sample consisted of 683 809 patients and 1 565 032 health events (fig 1).

### Exposures

The exposures of interest were the spring and autumn clock changes between 2008 and 2019. In England, the clocks went forward one hour at 1 am on the last Sunday in March and went back one hour at 2 am on the last Sunday in October. A complete list of these dates is provided in supplementary table S1.

### Outcomes

Our eight health conditions of interest were anxiety, acute major cardiovascular disease, depression, eating disorders, psychiatric conditions (accident and emergency only), road traffic injuries, self-harm, and sleep disorders (fig 1). We chose these conditions because previous research suggests that the clock changes affect cardiovascular disease,^4^ road traffic injuries,^5^ and depression,^6^ and we wanted to be able to compare our results with those of similar existing studies. The clock changes are also reported to affect the duration, efficiency (proportion of time in bed spent asleep), and fragmentation (number of awakenings) of sleep.^18^ ^41^ Examining the effects of the clock changes on sleep disorders therefore seemed logical, particularly given that our earlier study found electronic health records to be a valuable data source for measuring the prevalence of insomnia.^42^ We also wanted to extend the scope of the existing literature on the effect of the clock changes on mental health outcomes, with previous studies focusing on depression,^6^ suicide,^43^ and mental health visits to emergency departments,^44^ to explore other mental health conditions that may be better captured in primary care data. We therefore included self-harm (including suicide), eating disorders, and anxiety in our study. We were able to identify cardiovascular disease, road traffic injuries, and self-harm in all three healthcare datasets: primary care, HES APC, and HES A&E data. However, accident and emergency data are not specific enough to identify individual conditions such as depression, anxiety, sleep disorders, or eating disorders. We therefore included psychiatric conditions in accident and emergency data separately.

We defined health events in secondary care as the presence of a diagnosis code in a person’s health record that was entered during a hospital admission (HES APC data) or accident and emergency consultation (HES A&E data). In HES APC data, these were ICD-10 (international classification of diseases, 10th revision) codes. The HES A&E dataset has its own coding system. We identified road traffic injury and self-harm events by using the accident and emergency patient group code, which gives the reason for the accident and emergency episode. We identified cardiovascular disease and psychiatric conditions events on the basis of the patient group code combined with a two digit diagnosis code (see supplementary text S3). We defined health events in primary care as the presence of a medical code recorded during a general practice consultation. For most of our health conditions, these medical codes represented diagnoses. However, for sleep disorders, anxiety, and depression, medical codes often relate to symptoms rather than diagnoses. For these conditions, we counted medical codes as a health event if they were specific enough to identify the condition. However, where the codes related to symptoms that were not considered adequate to define the health condition, we counted the health event only if the patient also had a relevant prescription within the 90 day period either side of the symptom code. Detailed definitions of all outcomes are provided in supplementary text S3. We included both incident and prevalent events for all health conditions (including subsequent events), and we counted each record with a code for the condition as a separate event.

Where possible, we created potential code lists on the basis of existing, published lists (see https://github.com/MeldeLange/dst_cprd for details). Details of how we created medical code and ICD-10 code lists for outcomes when no existing code lists were available are provided in supplementary text S4. All preliminary code lists were checked by a general practitioner (SVE) with experience in using the codes in research and clinical practice. On the basis of the feedback, we refined the lists to ensure that only relevant codes were included. The final code lists used in the analyses can be found at https://github.com/MeldeLange/dst_cprd.

### Covariates

Our analysis was restricted to the eight weeks surrounding the clock changes. This meant that we could assume that no confounders of the effect of the clock changes on our outcomes existed. We considered several sociodemographic and lifestyle characteristics to be potential effect modifiers of the effect of the clock changes on health. We defined age and incident versus prevalent cases at the time of the clock change (exposure). Index of multiple deprivation data were from 2019. Sex was recorded only once in the primary care data. We defined body mass index, alcohol status, smoking status, and systolic and diastolic blood pressure at the most recent record before the clock change. We identified cardiovascular disease event subgroups on the basis of disease codes in primary care and HES APC data. Creation of subgroups for other outcomes or in accident and emergency data was impossible because the codes used were not sufficiently specific. Full details of how we derived all covariates are provided in supplementary table S2. Briefly, they consisted of two categories plus a category for missing values (if necessary). We chose different age cut-offs for the eight health outcomes so that we had equally sized binary categories based on the median value. We considered geographical region and year to be potential effect modifiers. Region consisted of nine categories based on the Strategic Health Authority of each general practice. Our study included data from 391 general practices in the North East, North West, Yorkshire and the Humber, East Midlands, West Midlands, East of England, London, South East, and South West (see supplementary table S3 for the number of general practices per region).

Other covariates that could affect the outcome were the Easter weekend in spring and day of the week. We considered the Easter weekend and day of the week to be competing exposures that may coincide with the clock change and could affect health outcomes but are not related to the clock change. Dates of the five day Easter weekends in our study period are provided in supplementary table S4.

### Statistical analysis

#### Primary analysis

We plotted the total number of events per day for each health outcome for the eight week period surrounding the spring and autumn clock changes. We also fitted a negative binomial regression model to plot the total number of events per day compared with the expected number of events for each day of the week on the basis of the average over the eight week period. We did negative binomial regression analyses, adjusted for the day of the week and region (and Easter weekend in spring), to estimate incidence rate ratios and 95% confidence intervals comparing the mean daily number of events (per year, per region) in the first week after the clock changes with those in the control period. The control period consisted of the four weeks before the clock changes and weeks 2-4 after the clock changes (supplementary figure S1a). Here the incidence rate ratio represents the relative change in the mean number of events per day (per year, per region) in the week after the clock change compared with the control period. We chose a one week exposure period because the effects of the clock changes on sleep are believed to last around a week.^41^ It also meant that our results were comparable to most of the studies examining the acute effects of the clock changes, which have focused on the first one or two weeks after the clock change.^4^ ^10^ We chose our control period so that we had a relatively narrow window around the clock change to reduce the risk of other time varying factors, such as other exposures or changes in the underlying population, biasing the results.^45^ In keeping with previous studies examining the health effects of the clock changes, we wanted to include weeks before and after the clock change in our control period so that we were not just capturing seasonal trends.^8^ ^46^ ^47^ ^48^ ^49^ We used negative binomial regression rather than Poisson regression to account for over-dispersion in the data. In our analyses, we split our data by year (12 years), region (nine regions), and day (56 days in an eight week period), which gave us 6048 data points in our primary analysis.

We adjusted the number of events per day to account for the fact that the Sunday of the clock changes had more or fewer hours than other days. We adjusted for the shortening of the Sunday of the spring clock change by dividing the number of events by 23 and multiplying by 24, and we accounted for the lengthening of the Sunday of the autumn clock change by dividing the number of events by 25 and multiplying by 24.

We used Stata version 18 for analyses. The data cleaning and analysis code written by MAdL was reviewed by KB. The complete code is available at https://github.com/MeldeLange/dst_cprd. This study meets all five CODE-EHR framework standards for using structured healthcare data in clinical research^50^ (see supplementary text S5 for the completed checklist). It was written according to the STROBE-RECORD checklist for observational studies using routinely collected health data^51^ (see supplementary text S6).

#### Secondary analyses

As a secondary analysis, we ran the primary analysis stratified by age, sex, Index of Multiple Deprivation, and incident versus prevalent cases. We also stratified anxiety, depression, psychiatric conditions in accident and emergency, self-harm, sleep disorders, and road traffic injuries by alcohol status; eating disorders by body mass index; and acute cardiovascular disease by body mass index, alcohol status, smoking status, systolic and diastolic blood pressure, and cardiovascular disease subgroup (see supplementary table S2 for details of how these covariates were derived). We used Cochran’s Q to test for differences between strata.

We also examined the number of health events over different post-clock change periods (see supplementary figures S1b-e). Specifically, we compared health events on the individual days of the Sunday to Saturday in the week after the clock change with a control period consisting of the same day in the four weeks before the clock change and weeks 2-4 after the change. This aimed to examine whether the clock changes were changing the daily timing of events rather than the total number of events across the whole week. Secondly, we compared health events on the Monday to Friday of the first week after the clock change with a control period of the Monday to Friday in the four weeks before the clock change and weeks 2-4 after the change. Thirdly, we compared health events in the first two weeks after the clock changes with a control period of the four weeks before the change and weeks 3-4 afterwards. Fourthly, we compared health events in the four weeks after the clock change with a control period of the four weeks before the clock changes. Whereas most previous studies examining the acute downstream health effects of the clock changes have found the largest effects in the first week,^5^ ^8^ ^49^ how long it takes for people’s sleep schedules to adapt may vary depending on the clock change, as well as individual characteristics such as chronotype, habitual sleep duration, and light exposure.^2^ ^52^ ^53^ Secondary analyses 3 and 4, therefore, aimed to explore the duration of the effect of clock changes on the health outcomes in our study.

#### Sensitivity analyses

We did two sensitivity analyses to examine the effect of a person having more than one event for the same outcome in the eight weeks surrounding one particular clock change. Firstly, for each clock change, we excluded anyone with an event for that outcome in weeks 12-5 before the clock change (that is, the eight weeks before our eight week study period). We then kept just the first event in the eight week study period for each individual. Secondly, we excluded the events of anyone with an extreme number of events (≥20) in a particular eight week period from the sub-dataset for those eight weeks. We kept only one randomly selected event for each remaining person in those eight weeks.

#### Negative control

We also did a negative exposure analysis to test whether our results were specific to the clock changes or were due to seasonality more generally. Here, we repeated the primary analysis but defined the exposed week as the fourth week before the clock change. The control period consisted of weeks 8-5 and weeks 3-1 before the clock change (see supplementary figure S1f).

### Patient and public involvement

In this study, we analysed routinely collected electronic health records data. Patients and the public were not directly involved in the design, conduct, reporting, or dissemination plans.

## Results

### Participant’s characteristics

In our study, 57% of participants were female, 64% were aged ≥40 years, and 26% were in the top fifth of deprivation (table 1). The characteristics of people who had an event in spring and autumn were similar. This suggests that our assumption that the characteristics of people having events does not change over time holds.

**Table 1:** Sample characteristics at each patient’s start of follow-up*. Values are numbers (percentages)

Characteristics	Total sample (n=683 809)†	Spring clock change (n=410 763)	Autumn clock change (n=424 264)
Sex:			
Male	294 482 (43.1)	174 981 (42.6)	180 635 (42.6)
Female	389 318 (56.9)	235 775 (57.4)	243 622 (57.4)
Missing	9 (0.0)	7 (0.0)	7 (0.0)
Age category, years:			
<10	14 323 (2.1)	7148 (1.7)	8260 (1.9)
10-19	51 783 (7.6)	28 783 (7.0)	31 263 (7.4)
20-29	86 196 (12.6)	49 808 (12.1)	52 911 (12.5)
30-39	95 629 (14.0)	56 299 (13.7)	59 456 (14.0)
40-49	105 432 (15.4)	63 531 (15.5)	65 852 (15.5)
50-59	88 482 (12.9)	53 497 (13.0)	54 900 (12.9)
60-69	88 393 (12.9)	54 169 (13.2)	55 425 (13.1)
70-79	88 569 (13.0)	56 383 (13.7)	56 369 (13.3)
80-89	56 143 (8.2)	35 696 (8.7)	34 671 (8.2)
≥90	8859 (1.3)	5449 (1.3)	5157 (1.2)
Index of Multiple Deprivation:			
Most deprived	175 621 (25.7)	107 208 (26.1)	110 765 (26.1)
Rest	507 650 (74.2)	303 198 (73.8)	313 167 (73.8)
Missing	538 (0.1)	357 (0.1)	332 (0.1)

* Start of follow-up=latest date of patient’s start date in Clinical Practice Research Datalink GOLD or study start date (2 March 2008); Index of Multiple Deprivation is from 2019.

† Patients can appear in both spring and autumn analyses, so total sample is less than spring and autumn samples combined.

### Primary analysis

Supplementary figures S2-S17 show the total number of events per day over the eight week clock change periods for each health outcome over the study period (years 2008-19); supplementary figures S18-S33 show the total number of events per day compared with the expected number of events for that day of the week. We found little evidence of seasonal patterns, such as a general increase or decrease in event numbers over the eight week period. However, the overriding pattern seen in the graphs was that the number of health events varied according to the day of the week, with the largest variation being between weekdays and weekends. This is most likely an artefact of the healthcare system, as people are less likely to visit their general practitioner on a weekend.

**Fig 2:**
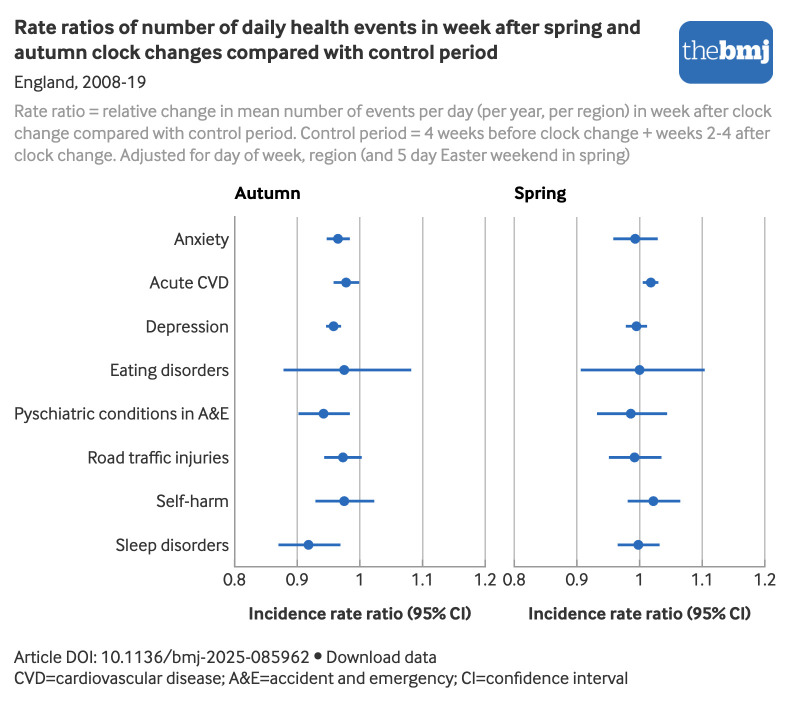
Adjusted rate ratios of mean number of health events per day in week after spring and autumn clock changes compared with control period (England, 2008-19). An interactive version of this graphic and downloadable data are available at https://public.flourish.studio/visualisation/26428983/

In spring, the mean number of major acute cardiovascular disease events per day increased by 2% in the week after the clock changes compared with the control period (from 49.3 events per day (per year, per region) to 49.6) (incidence rate ratio 1.02, 95% confidence interval (CI) 1.005 to 1.030) (fig 2; supplementary table S5). However, both sensitivity analyses conducted to explore the effect of individual patients having multiple events in the same eight week clock change period indicated that this result may have been driven by some individuals having multiple events and is, therefore, not a true effect of the clock change (see supplementary tables S6 and S7). We saw no differences in event numbers in the primary analysis for the other health outcomes in the full week after the spring clock change compared with the control period. The results of the sensitivity analysis for these outcomes were very similar to the primary analysis, suggesting that people having multiple events did not affect these results.

In the week after the autumn clock change, we saw a reduction in the mean number of events per day for anxiety disorders (−3%) (incidence rate ratio 0.97, 95% CI 0.95 to 0.98) (from 17.3 events per day (per year, per region) to 16.7), major acute cardiovascular disease (−2%) (0.98, 0.96 to 0.999) (from 50.0 events per day (per year, per region) to 48.9), depression (−4%) (0.96, 0.95 to 0.97) (from 44.6 events per day (per year, per region) to 42.7), psychiatric conditions in accident and emergency (−6%) (0.94, 0.90 to 0.98) (from 3.5 events per day (per year, per region) to 3.3), and sleep disorders (−8%) (0.92, 0.87 to 0.97) (from 5.4 events per day (per year, per region) to 4.9) (fig 2; supplementary table S5).

### Secondary analyses

The decline in event rates for anxiety, depression, and psychiatric conditions in accident and emergency in the week after the autumn clock change was driven by people in the youngest age categories (see supplementary table S8 for stratified results and Cochran’s Q test of heterogeneity P values). For example, the mean number of psychiatric condition events recorded in accident and emergency fell by 10% in people aged 10-35 years (incidence rate ratio 0.90, 95% CI 0.843 to 0.954) (from 1.7 events per day (per year, per region) to 1.5), whereas no differences existed in those aged over 35 (0.99, 95% CI 0.930 to 1.044). In addition, we saw differences by sex and alcohol consumption for psychiatric conditions in accident and emergency. Women had an 11% decrease in psychiatric conditions in the week after the autumn clock change (incidence rate ratio 0.89, 95% CI 0.835 to 0.947) (from 1.8 events per day (per year, per region) to 1.6), whereas men showed no difference from the control period. In addition, current drinkers and those with missing alcohol status had a 5% (from 1.52 events per day (per year, per region) to 1.44) and 13% (from 1.23 events per day (per year, per region) to 1.08) decrease in psychiatric events in the week after the autumn clock change, respectively, whereas for non/ex-drinkers we found weak evidence against the null hypothesis.

Our secondary analysis showed considerable variability in daily event numbers over the first week after the clock change (see supplementary table S5). In many cases, increases or decreases seen on particular days did not persist for the whole week, suggesting that the clock changes could have affected the timing of events across the week, rather than the total number of events within the week. For example, the mean number of depression events dropped by 8% on the Sunday of the spring clock change (from 8.3 (per year, per region) to 7.7), whereas the mean number of road traffic injuries increased by 2% on the same day (from 4.44 (per year, per region) to 4.48). However, we saw no effect for either of these outcomes over the entire first week.

Overall, the results of our analysis of events on the Monday-Friday in the first week after the clock changes were highly consistent with our primary analysis. Analyses comparing the number of events in the two and four weeks after the clock changes with their respective control periods (two week analysis control period: four weeks before the clock change and weeks 3-4 after; four week analysis control period: four weeks before the clock change) found few differences (supplementary table S5). However, event rates for depression were 2% lower than during the control period in the two weeks after the autumn clock change (43.6 events per day (per year, per region) versus 44.6). The number of sleep disorder events recorded was 7% lower than during the control period in the two weeks (5.0 events per day (per year, per region) versus 5.4) and 3% lower in the four weeks (5.2 events per day (per year, per region) versus 5.4) after the autumn clock change.

### Negative control

Our negative exposure analysis (in which the exposed week was the fourth week before the clock change and the control period consisted of weeks 8-5 and weeks 3-1 before the clock change) detected little evidence of the downward trends in autumn seen in the main analysis (supplementary table S9). In spring, it detected an increase in sleep disorders in the week after the negative exposure, which was not found in our main analysis.

## Discussion

In this population based retrospective cohort study, we found a reduction in the number of events (diagnoses or symptoms and accompanying prescription for mental health conditions in primary care) recorded for multiple health conditions in the week after the autumn clock change. This included anxiety, major acute cardiovascular disease, depression, psychiatric conditions in accident and emergency, and sleep disorders. The reduction in sleep disorders persisted for four weeks, although the effect attenuated over that period. We found little evidence that the spring clock changes were associated with a difference in the number of health events recorded in the week after the change.

### Comparison with other studies

The latest meta-analysis of the effects of the clock changes on acute myocardial infarction reported a 4% increase after the spring clock change, but substantial heterogeneity was present in study results and designs.^4^ We found initial evidence of a 2% increased risk of acute cardiovascular disease in the week after the spring clock change, which was not supported by sensitivity analyses examining the effect of individuals having multiple events. Our results may differ because we included primary care data, used data only from England, with its unique geographical location and healthcare system, and included other cardiovascular disease conditions, such as stroke. Studies have pointed to the clock changes affecting the day of the week when strokes occur rather than the total number of events across the post-clock change week.^8^ In line with our results, other studies have found no difference in overall hospital admissions for or deaths from cardiovascular disease after the spring change.^54^ ^55^

Our null findings for road traffic injuries in spring align with those of a systematic review that reported inconsistent findings.^10^ Our results are also consistent with the only previous study to include data from England, which detected a very small absolute reduction in police recorded road traffic casualties after the spring clock change (−0.003%).^11^ Meanwhile, our results for depression are supported by studies that have also found little evidence of an effect of the spring clock change on depression and other mental health conditions.^6^ ^13^ ^44^

A Danish study found that the autumn clock change was associated with an 11% increase in unipolar depressive episodes in hospitals.^6^ This discrepancy with our results (4% decrease) may be because our analyses included primary care data, which capture fewer acute events. Some misclassification could also be present in our study owing to a delay existing between people experiencing depression and seeing a clinician in primary care. In addition, the contrasting results may be due to disparities in sunrise and sunset times between England and Denmark as a result of their geographical locations.

The meta-analysis of acute myocardial infarction mentioned above reported that the autumn clock change had no effect.^4^ Other studies, however, have reported a 30% reduction in cardiac arrests,^56^ a 7.5% decrease in hospital admissions due to cardiovascular disease,^7^ and a 3% reduction in all cause mortality after the autumn change.^57^ Our 2% decrease in cardiovascular disease events after the autumn clock change is therefore generally in line with estimates reported by the existing literature. The effect of the clock changes on sleep disorders and anxiety have not been explored previously, so our evidence of a decline in these conditions after the autumn clock change represents a novel contribution to the literature.

### Strengths and limitations of study

This comprehensive examination of the effects of the daylight saving time clock changes on the health of the English population benefitted from access to 12 years of data from a large dataset of linked electronic health records that is broadly representative of the UK population.^36^ Our inclusion of primary care, hospital admission, and accident and emergency data meant that our study captured a more complete picture of the effect of the clock changes on demand for health services than previous studies. Furthermore, the breadth of data available meant that we were able to examine multiple different health outcomes (some not examined in this context before) in the same dataset. In addition, we did several sensitivity analyses to evaluate the robustness of our results.

The quality of routinely collected health data relies on the accuracy and completeness of data recorded by health professionals.^58^ CPRD GOLD data have been found to have high levels of validity,^58^ and we used data only from general practices that CPRD considered to be “up to standard.” Nevertheless, mental health outcomes and sleep disorders are particularly difficult to define in electronic health records, with wide variability in the definitions and codes used by researchers to identify cases.^59^ For this reason, we used definitions and code lists for these outcomes created by an earlier study after the authors systematically reviewed previously used code lists.^60^ We have also fully reported the outcome definitions and code lists that we used in this study (see supplementary text S3 and https://github.com/MeldeLange/dst_cprd, respectively).

However, electronic health records include only health events for which the individual seeks medical help. More subtle effects on health, such as a slight dip in mood, might not have been captured in this study. Additionally, the date of a health event in electronic health records represents the date that a clinician recorded the event. This is not necessarily the same as the date of the onset of the health condition, as patients may wait to see whether symptoms disappear on their own, face delays in accessing a healthcare professional, or experience delays between reporting symptoms to a clinician and receiving a diagnosis. This means that in this study, potential for misclassification in primary care data could exist, whereby people with a diagnosis after the clock change may have been experiencing symptoms for non-acute conditions, such as anxiety and depression, before the change but sought or received help only after the change. This could also have led to diagnoses affected by the clock changes that fell beyond our eight week study period. However, the effect of a delay between presenting to a general practitioner with symptoms and receiving an official diagnosis may have been limited somewhat in our study by the fact that for sleep disorders, anxiety, and depression (for which codes tend to relate to symptoms rather than diagnoses) we included symptoms recorded by a general practitioner as a health event if a relevant concurrent prescription accompanied them.

Although we controlled for day of the week, geographical region, and the Easter weekend in our analyses, we could not adjust for the competing exposure of school half term holidays, as these vary widely throughout England. Our stratified analyses could also have lacked statistical power, particularly for less common health outcomes such as eating disorders. In addition, the asymmetry of the control period in our main analysis (four weeks before the clock change plus weeks 2-4 after) could potentially mean that we captured some seasonal trends.

### Implications for policy

Our study contributes to the ongoing debate about England’s clock change policy. However, the results should be interpreted in the context of the complete literature on daylight saving time clock changes and health.^61^ In aggregate, our evidence and the rest of the literature suggest that the effects of the clock changes on health are not consistently harmful.^62^ Our study found that, in England, the autumn clock change is associated with a reduction in demand for NHS services for cardiovascular disease, sleep disorders, and mental health disorders. The reductions in health events reported in this study are relatively small in terms of percentages (decreases of 2-8%). However, because the clock changes affect the whole population of England, the underlying change in number of events is high. Even small effects can have substantial implications for demand for NHS appointments and prescriptions. Furthermore, reductions in conditions such as sleep disorders could prevent people going on to develop other mental and physical health problems such as diabetes, depression, dementia, and cardiovascular disease.^63^ ^64^ ^65^ ^66^

The clock changes are believed to influence health via a combination of sleep and light. Examining the mediators through which the clock changes affect health was not possible in our study as we did not have data on sleep or light exposure. However, the extra sleep gained over the autumn clock change could reduce cardiovascular disease risk by helping to control cortisol concentrations, inflammation, blood pressure, and insulin sensitivity.^18^ ^46^ ^47^ ^67^ Sufficient sleep is also vital for good mental health.^64^ ^68^

The fact that we found little evidence that the spring clock change (when people lose an hour of sleep) was associated with an increase in health events, combined with the fact that people may gain only around half an hour of sleep over the autumn clock change,^18^ suggests that morning light may be particularly important. Despite the clocks moving forward, the lack of morning light immediately after the spring clock change is theoretical, as it is still light when most people wake up.^34^ However, after the autumn clock change, in late October, people are exposed to more sunlight in the mornings and less sunlight in the evenings.^6^ Morning sunlight exposure is important for resetting our circadian rhythm each day (it naturally runs just over 24 hours)^69^ ^70^ and improving sleep.^34^ It may also reduce the risk of cardiovascular disease by lowering blood pressure.^71^ Morning light also improves mental health, as evidenced by the fact that morning light therapy is an effective treatment for depression.^72^ ^73^ This could be a potential reason for the discrepancy in findings between our study and the study of depression in Denmark, as sunrise is generally earlier in England than in Denmark. As a result, people in England will benefit from a greater increase in morning sunlight after the autumn clock change than people in Denmark.

In this study, we focused on the short term effects of the clock changes on health. However, debates about whether to abolish daylight saving time should also account for the longer term effects of observing it for seven months of the year. These longer term effects are more difficult to ascertain because of confounding from other seasonal factors.^34^ ^74^ ^75^ That said, our results can be interpreted in several ways. On the one hand, we found that the clock changes were not associated with negative health effects (the spring clock change was not associated with changes in the number of health events, whereas the autumn clock change was associated with a reduction in health events). On this basis, it could be argued that we should keep the current system of clock changes. Moving the autumn clock change forward a couple of weeks to early October to avoid the period of dark mornings just before the autumn clock change could also potentially be beneficial.^76^ On the other hand, our results suggest that sleep and morning light exposure are important for our mental and physical health. It could therefore be argued that, if England does abolish the clock changes, we should opt for permanent standard time (GMT), which prioritises morning light and sleep, rather than permanent daylight saving time.^34^

Further research should investigate the mechanisms underlying the possible reduction in mental health conditions observed after the autumn clock change in this study. Future studies could also use data from countries such as Australia, where daylight saving time is observed only in certain states, to compare rates of health events under daylight saving time and non-daylight saving time conditions in geographically similar locations during the same time period. Additional work should also analyse the emerging data from countries that have recently abolished daylight saving time clock changes to monitor the long term effects of permanent daylight saving time and standard time on health. Examining the effects of the clock changes specifically on the paediatric population would also be valuable, as would conducting cross-country comparisons to examine whether the effect of the clock changes varies according to the timing of sunrise and sunset.

### Conclusions

In summary, the week after the autumn clock change was associated with a reduction in cardiovascular disease, sleep disorders, and mental health disorders. However, little evidence suggested that the spring clock change was associated with changes in the number of health events. Future research should explore the mechanisms underlying the reduction in health events that we observed after the autumn clock change.

## What is already known on this topic

Some studies (mainly outside the UK) suggest that the daylight saving time clock changes, particularly the spring clock change, have a detrimental effect on population health
On this basis, sleep societies have unanimously called for the clock changes to be abolished and recommend that GMT be adopted year round
In 2019 the EU voted to end the practice of changing the clocks, but whether England will do the same is not clear

## What this study adds

This extensive study of the effects of the daylight saving time clock changes on health in England used a large, representative general practice and hospital dataset
The study examined multiple health conditions and estimates the effects of the clock changes on demand for NHS services
The week after the autumn clock change was associated with a decrease in health events for sleep disorders, cardiovascular disease, anxiety, depression, and psychiatric conditions

## Data Availability

This study is based in part on data from the Clinical Practice Research Datalink obtained under licence from the UK Medicines and Healthcare products Regulatory Agency. The data are provided by patients and collected by the NHS as part of their care and support. The Office for National Statistics (ONS) is the provider of the ONS data contained within the dataset. HES and ONS data, copyright (2021/2022), are re-used with the permission of the Health and Social Care Information Centre. All rights reserved. The interpretation and conclusions contained in this study are those of the authors alone. The raw data used in this study are not publicly available and cannot be shared. Access to CPRD and linked data are subject to protocol approval via CPRD’s Research Data Governance (RDG) Process. See https://www.cprd.com/data-access for further details. Full disease code lists and Stata code for analyses are available at https://github.com/MeldeLange/dst_cprd.

## Web extra

Extra material supplied by authors

Web appendix: Supplementary materials

## Notes

Contributors: NMD and MAdL developed the study concept. MAdL did the data analysis and drafted the first version of the manuscript. SVE checked the medical code and ICD-10 and HES A&E disease code lists used in the analysis. KB reviewed the code used to clean and analyse the data. MAdL, NMD, RCR, KT, KB, CYS, and SVE interpreted the data, reviewed and revised the manuscript, and approved the final version as submitted. The corresponding author attests that all listed authors meet authorship criteria and that no others meeting the criteria have been omitted. MAdL is the guarantor.

Transparency: The lead author (the manuscript’s guarantor) affirms that the manuscript is an honest, accurate, and transparent account of the study being reported; that no important aspects of the study have been omitted; and that any discrepancies from the study as originally planned (and, if relevant, registered) have been explained.

Dissemination to participants and related patient and public communities: The findings from this study will be shared with the public and healthcare professionals via press releases, social media coverage, and presentations at scientific conferences. CPRD provides lay summaries of all approved protocols and details of all published studies on its website (www.cprd.com).

Provenance and peer review: Not commissioned; externally peer reviewed.
