# Supplementary material for: Acute effects of daylight saving time clock changes on mental and physical health in England: population based retrospective cohort study
Source: BMJ. 2025 Dec 17;391:e085962. doi: 10.1136/bmj-2025-085962 (PMC12709669; doi:10.1136/bmj-2025-085962)
Supplement: Supplementary file 1 — Web appendix: Supplementary materials [file delm085962.ww1.pdf]

The Effects of Daylight Saving Time Clock Changes on Health in England: Evidence from the Clinical Practice Research Datalink (CPRD)

Supplementary material

Contents

Text S1: CPRD Protocol 22/002468 1

Text S2: Minor deviations from protocol 24

Text S3: Definitions of outcomes 25

Text S4: Creation of code lists from scratch 33

Text S5: CODE-EHR Checklist 34

Text S6: STROBE-RECORD Statement 36

Table S1: List of dates of DST clock changes 2008-2019 46

Table S2: Derivation of covariates 46

Table S3: Number of GP practices per region 48

Table S4: List of dates of 5-day Easter weekends 2008-2019 48

Table S5: Adjusted* rate ratios of the number of events in the 1 week (including by day and Monday-Friday), 2 week and 4 week periods after the Spring and Autumn clock changes (England, 2008-2019) versus control periods** 49

Table S6: Sensitivity analysis: Adjusted* rate ratios of the number of health events in the week after the Spring and Autumn clock changes (England, 2008-2019) versus control period**, with events of patients with an event for the same health condition in the 8 weeks before the study period removed for that clock change and only the first event for that health condition in the 8 week period kept for the remaining patients 51

Table S7: Sensitivity analysis: Adjusted* rate ratios of the number of health events in the week after the Spring and Autumn clock changes (England, 2008-2019) versus control period**, with events for people with 20 or more events for the same health condition in a single 8-week clock change period excluded from that period & one random event kept for the remaining patients 51

Table S8: Secondary analysis: Adjusted rate ratios of the number of health events in the week after the Spring and Autumn clock changes compared to the control period (England, 2008-2019), stratified by sociodemographics 52

Table S9: Negative control exposure analysis: Adjusted* rate ratios of the number of health events in the week after the Spring and Autumn negative controls (4 weeks before the clock changes) (England, 2008-2019) versus control period** . 58

Figure S1: Exposure and control periods for primary and secondary analyses. ... 59

Figure S2: Anxiety events per day over the Spring clock changes. 62

Figure S3: CVD events per day over the Spring clock changes. 62

Figure S4: Depression events per day over the Spring clock changes. 63

Figure S5: Eating disorder events per day over the Spring clock changes. 63

Figure S6: Psychiatric conditions events per day over the Spring clock changes. 64

Figure S7: Road traffic injury events per day over the Spring clock changes. 64

Figure S8: Self-harm events per day over the Spring clock changes. 65

Figure S9: Sleep disorder events per day over the Spring clock changes. 65

Figure S10: Anxiety events per day over the Autumn clock changes. 66

Figure S11: CVD events per day over the Autumn clock changes. 66

Figure S12: Depression events per day over the Autumn clock changes. 67

Figure S13: Eating disorder events per day over the Autumn clock changes. 67

Figure S14: Psychiatric conditions events per day over the Autumn clock changes. 68

Figure S15: Road traffic injuries events per day over the Autumn clock changes. 68

Figure S16: Self-harm events per day over the Autumn clock changes. 69

Figure S17: Sleep disorders events per day over the Autumn clock changes. 69

Figure S18: Anxiety events per day over the Spring clock changes versus expected number of events per day. 70

Figure S19: CVD events per day over the Spring clock changes versus expected number of events per day. 70

Figure S26: Anxiety events per day over the Autumn clock changes versus expected number of events per day. 75

Figure S27: CVD events per day over the Autumn clock changes versus expected number of events per day. 75

Figure S28: Depression events per day over the Autumn clock changes versus expected number of events per day. 76

Figure S29: Eating disorder events per day over the Autumn clock changes versus expected number of events per day. 76

Figure S30: Psychiatric conditions events per day over the Autumn clock changes versus expected number of events per day. 77

Figure S32: Self-harm events per day over the Autumn clock changes versus expected number of events per day. 78

Figure S33: Sleep disorder events per day over the Autumn clock changes versus expected number of events per day. 78

Text S1: CPRD Protocol 22/002468

CPRD Research Data Governance (RDG) Application Template

ALL APPLICATIONS MUST BE COMPLETED AND SUBMITTED VIA THE CPRD ELECTRONIC RESEARCH APPLICATION PORTAL (eRAP) www.erap.cprd.com

Applicants may use this template offline to prepare their research application, prior to submission on eRAP. Applicants must also read CPRD's Research Data Governance (RDG) Guidance on how to complete their application found on the eRAP landing page under Related resources (https://www.erap.cprd.com/)

GENERAL INFORMATION ABOUT THE PROPOSED RESEARCH STUDY
**Study Title (Max. 255 characters including spaces)**<br><br>**Estimating the Effects of Daylight Saving Time Clock Changes on Cardiovascular Outcomes, Depressive Symptoms and Road Traffic Injuries in England**
**Research Area** (place '**X**' in all boxes that apply)

Drug Safety		Economics	
Drug Utilisation		Pharmacoeconomics	
Drug Effectiveness		Pharmacoepidemiology	
Disease Epidemiology	x	Methodological	
Health Services Delivery			

Does this protocol describe an observational study using purely CPRD data?

Yes	**x**	No	

Does this protocol involve requesting any additional information from GPs, or contact with patients?

Yes		No	**x**

If yes, provide the reference number:

Chief Investigator

Title:	Professor
Full name:	Kate Tilling
Job title:	Professor of Medical Statistics and MRC Investigator
Affiliation/organisation:	University of Bristol
Email address:	Kate.Tilling@bristol.ac.uk
CV Number (if applicable):	n/a
Will this person be analysing the data? (Y/N)	N

Corresponding Applicant

Title:	Mrs
Full name:	Melanie de Lange
Job title:	PhD Student
Affiliation/organisation:	University of Bristol
Email address:	melanie.delange.2018@bristol.ac.uk
CV Number (if applicable):	n/a
Will this person be analysing the data? (Y/N)	Y

List of all investigators/collaborators

Title:	Dr
Full name:	Kate Birnie
Job title:	Senior Research Fellow
Affiliation/organisation:	University of Bristol
Email address:	Kate.Birnie@bristol.ac.uk
CV Number (if applicable):	465_20
Will this person be analysing the data? (Y/N)	Y

Title:	Professor
Full name:	Neil Davies
Job title:	Professor of Medical Statistics
Affiliation/organisation:	University College London
Email address:	neil.m.davies@ucl.ac.uk
CV Number (if applicable):	456_15CEL
Will this person be analysing the data? (Y/N)	N

Title:	Dr
Full name:	Sophie Eastwood
Job title:	Clinical Research Fellow
Affiliation/organisation:	University College London
Email address:	sophie.eastwood@ucl.ac.uk
CV Number (if applicable):	n/a

Will this person be analysing the data? (Y/N)	N

ACCESS TO THE DATA

Sponsor of the study

Institution/Organisation:	Medical School, University of Bristol
Address:	Oakfield House, Oakfield Grove, Clifton, Bristol, BS8 2BN

Funding source for the study

Same as Sponsor?	Yes		No	X
Institution/Organisation:	Wellcome Trust			
Address:	Gibbs Building, 215 Euston Road, London, NW1 2BE			

Institution conducting the research

Same as Sponsor?	Yes	X	No	
Institution/Organisation:				
Address:				

Data Access Arrangements

Indicate with an '**X**' the method that will be used to access the data for this study:

Study-specific Dataset Agreement	X

Institutional Multi-study Licence	
Institution Name	
Institution Address	

Will the dataset be extracted by CPRD?

Yes	X	No	

If yes, provide the reference number:

Data Processor(s):

Processing	X
Accessing	X
Storing	X

Processing area (UK/EEA/Worldwide)	UK
Organisation name	Medical School, University of Bristol
Organisation address	Oakfield House, Oakfield Grove, Clifton, Bristol, BS8 2BN

INFORMATION ON DATA

Primary care data (place '**X**' in all boxes that apply)

CPRD GOLD	X	CPRD Aurum	

Reference number (if applicable):

Please select any linked data or data products being requested

Patient Level Data (place '**X**' in all boxes that apply)

ONS Death Registration Data		NCRAS Cancer Registration Data	
HES Admitted Patient Care	X	NCRAS Systemic Anti-Cancer Treatment (SACT) data	
HES Outpatient		NCRAS National Radiotherapy Dataset (RTDS) data	
HES Accident and Emergency	X	Second Generation Surveillance System (SGSS, COVID-19)	
HES Diagnostic Imaging Dataset		COVID-19 Hospitalisations in England Surveillance System (CHESS)	
Mental Health Data Set (MHDS)			
CPRD Mother Baby Link			
Pregnancy Register			

Area Level Data (place '**X**' in <u>one</u> Practice / Patient level box that may apply)

**Practice level (UK)**		**Patient level (England only)**	
Practice Level Index of Multiple Deprivation		Patient Level Index of Multiple Deprivation	X

Practice Level Index of Multiple Deprivation Domains		Patient Level Index of Multiple Deprivation Domains	
Practice Level Carstairs Index for 2011 Census (Excluding Northern Ireland)		Patient Level Carstairs Index for 2011 Census	
2011 Rural-Urban Classification at LSOA level		2011 Rural-Urban Classification at LSOA level	
		Patient Level Townsend Score	

Reference / Protocol number (where applicable):

Are you requesting linkage to a dataset not listed above?

Yes		No	**X**

If yes, provide the Non-Standard Linkage reference number:

Does any person named in this application already have access to any of these data in a patient identifiable form, or associated with an identifiable patient index?

Yes		No	**X**

If yes, provide further details:

PART 2: PROTOCOL INFORMATION

**Applicants must complete all sections**
**Study Title**<br><br>**Estimating the Effects of Daylight Saving Time Clock Changes on Cardiovascular Outcomes, Depressive Symptoms and Road Traffic Injuries in England**
**Lay Summary (Max. 250 words)**<br><br>**Exploring the effects of Daylight Saving Time (DST) Clock Changes on Cardiovascular Disease, Depression and Road Traffic Injuries in England**<br><br>There is growing evidence that daylight saving clock changes (the clocks moving one hour forward in spring and one hour back in autumn) may have adverse effects on people's health due to sleep deprivation and the disruption of biological rhythms. For example, studies outside of England have shown that the number of depressive episodes, heart attacks, strokes and fatal road traffic accidents increases in the weeks after the clock changes. This has prompted countries such as the US and the EU to vote to end DST clock changes. However, it is unclear whether DST will be abolished in England.<br><br>The purpose of this research is to estimate the effect of daylight savings time clock changes on the health of the English population. To do this we will compare the number of GP and hospital visits for cardiovascular disease, depression and road traffic injuries in the 4 weeks directly before and after the clock changes.<br><br>This data will provide evidence as to whether England should abolish DST clock changes. It will also offer insights into the wider effects of sleep and circadian disruption on mental and physical health.

Technical Summary (Max. 300 words)

Growing evidence suggests that daylight saving time (DST) clock changes (one hour forward in spring and one hour back in autumn) may have adverse effects on population health, likely via sleep deprivation and circadian disruption. For example, several studies using small datasets from outside England have reported increased incidence of depressive episodes, myocardial infarction, atrial fibrillation, strokes and fatal traffic accidents in the weeks immediately following the clock changes. This has prompted the US and the EU to vote to end DST clock changes. However, it is currently unclear whether England will do the same.

The aim of this study is to estimate the effects of DST transitions on cardiovascular disease, depression and road traffic injuries in the English population. Using CPRD and HES data for 2008-2022, regression discontinuity analysis will be used to compare the average number of primary and secondary care visits for these health outcomes in the 4 weeks before and after the spring and autumn clock changes. Regression discontinuity designs are a statistical method that uses a specific threshold or cut-off point (here the time of DST clock change) to estimate the causal, real-world, effects of policies. Differences in the number of visits will be compared in the 1- (modelled daily), 2- and 4-week periods before and after the clock changes.

Research findings will help to quantify the effects of DST clock changes on English population health by estimating the number of incident cases of disease and recurrent events attributable to DST clock changes. The results will be triangulated to formulate policy recommendations as to whether the English government should abolish DST clock changes. More broadly, the research will also offer insights into the wider effects of sleep and circadian disruption on mental and physical health.

Outcomes to be Measured

Primary outcomes

All primary/secondary care visits (incident & prevalent cases) in the 1- (modelled daily), 2- and 4-week periods before and after the clock changes, stratified by age (10 year age bands), for:

Depression (aged ≥10*).

Road traffic injuries (all ages).

Cardiovascular disease (aged ≥40**).

*Age ≥10 was chosen to ensure we capture adolescents who may be disproportionately affected by the sleep deprivation caused by the clock changes due to a shift towards having a later chronotype (going to bed and getting up later) during adolescence.

**The age at which people become eligible for their NHS health check and are considered at higher risk of cardiovascular disease.

Secondary outcomes

All primary/secondary care visits (incident & prevalent cases) in the 1- (modelled daily), 2- and 4-week periods before and after the clock changes, aged ≥10, stratified by age (10 year age bands), for:

Sleep disorders. This analysis will be exploratory, and will only be conducted if it is possible to adequately define sleep disorders within CPRD data.

Other mental health (anxiety & self-harm) & eating disorders.

The number of subsequent hospitalisations/referrals for depression/cardiovascular disease, or deaths amongst those visiting primary/secondary care for depression/cardiovascular disease in the 1-, 2- and 4-week periods before and after the clock changes.

Detailed definition of outcomes

Cardiovascular disease, road traffic injuries, self-harm and eating disorders will be defined using ICD10, Read codes and HES A&E codes.

Depression and anxiety will be defined using ICD-10, Read codes and HES A&E codes.

Read codes alone will be used to define depression/anxiety if the code is specific enough (see code lists C1 and C6 for depression/anxiety diagnoses).

However, where Read codes relate to symptoms of depression/anxiety (see code lists C2 and C7 for depression/anxiety symptoms) that are not considered adequate to define depression/anxiety, patients will only be counted as having depression/anxiety if they have been prescribed a drug used to treat depression within 90 days.

Sleep disorders will be defined using ICD-10 and Read codes.

Sleep disorders are difficult to define which means that most Read codes are not specific and relate to symptoms rather than diagnoses. Patients will therefore only counted as having a sleep disorder if they have a Read code for a sleep disorder diagnosis or symptom and have been prescribed a drug used to treat sleep disorders within 90 days.

See Appendix A for dates of clock changes, Appendix B for preliminary ICD10 codes, Appendix C for preliminary Read v2 codes, Appendix D for preliminary prescription codes and Appendix E for HES A&E codes.

*Notes to reviewer:

Having death as an outcome would be interesting, but unfortunately we do not have the budget to buy the linked ONS data.

Objectives, Specific Aims and Rationale

Aims and objectives

The overall aim of this research is to estimate the effect of daylight savings time clock changes on the health of the English population.

More specifically we want to:

Estimate the effects of DST clock changes on depression, road traffic injuries and cardiovascular disease.

Gain insights into the broader effects of sleep deprivation and circadian disruption on mental and physical health, including on sleep disorders, anxiety, self-harm, eating disorders.

Make policy recommendations as to whether England should abolish DST clock changes.

Rationale

Knowledge/information to be gained: By quantifying the health effects of DST clock changes on the English population the knowledge gained from the study will help to build an evidence base which can then be used to inform public health policy relating to whether the England should abolish DST clock changes. The findings will also improve our understanding of the effects of sleep deprivation and circadian disruption on mental and physical health.

Primary hypothesis to be tested: Primary/secondary care visits for depression, road traffic injuries and cardiovascular disease will be higher in the weeks after DST clock changes than in the weeks prior to the clock changes.

How achievement of objectives will further the research aim: In achieving our objectives we will have conducted one of the largest studies of the effects of DST on health in the UK to date. As well as informing English policy, the findings also have relevance to other countries considering abolishing DST clock changes. More widely, the research will improve our understanding of how sleep and circadian rhythms affect physical and mental health.

Study Background

Switching to daylight saving time (DST) involves moving the clocks one hour forwards in spring and one hour backwards in autumn[1]. It was first introduced during World War One[2] as a way of maximising exposure to daylight during the working day[1] and subsequently reducing energy use[3]. DST is now in operation in over 70 countries, including the UK, and affects over a quarter of the world's population[1],[4].

There is growing evidence that DST clock changes may have adverse effects on population health[1], likely via effects of sleep deprivation and circadian disruption[5]. For example, several studies using small datasets from outside of England have reported increased incidence of depressive episodes[1], myocardial infarction (MI)[6], atrial fibrillation[7], strokes[8] and fatal traffic accidents[9] in the weeks immediately following the clock changes. The likely adverse health effects of DST transitions has prompted calls for DST to be abolished and a number of countries are currently reconsidering its use[3,10]. Most prominently, in March 2019 the European Union voted to end DST after 2021[11]. However, it is currently unclear whether England will do the same.

Although previous studies have generally found consistent effects of DST on health outcomes, most have used relatively small datasets limiting the precision of effect estimates. For example, a 2018 review noted that the combined number of observations from 6 previous studies of DST and MI amounted to less than 90,000 individuals[12]. Furthermore, it is not clear to what extent previous findings can be generalised across countries with different climates, lifestyles and healthcare systems[6].

Study Type

This study will be descriptive in that it will describe and compare the frequency of primary and secondary care visits for depression, road traffic injuries, cardiovascular disease, sleep disorders, anxiety, self-harm and eating disorders within certain time periods.

It will also be hypothesis testing in that we will be looking at whether there is a discontinuity in, and statistical difference between, the number of visits before and after the clock changes. Our hypothesis is that there will be a difference and that the number of visits will be higher in the weeks after the clock changes.

Study Design

This research utilises a regression discontinuity design. Regression discontinuity designs are a statistical method that uses a specific threshold or cut-off point to estimate the causal effects of interventions. They are particularly valuable in evaluating the real world effects of policies, which inherently cannot be examined in a randomised control trial[13]. Here the DST clock changes act as a cut-off point determining whether people are exposed (the weeks after the clock changes) or unexposed (weeks before the clock changes). A recent meta-analysis comparing the results of regression discontinuity studies and randomised control trials concluded that regression discontinuity studies have high levels of interval validity[14].

We will also conduct stratified subgroup analysis to explore whether the effects of the clock changes are modified by factors such as year, incident vs. prevalent case, sex, age, ethnicity, socioeconomic position, alcohol, smoking, BMI, blood pressure, co-morbidities and disease subgroups.

Feasibility counts

Table 1 presents feasibility counts for our 3 primary outcomes. Counts for depression and cardiovascular disease were taken from existing CPRD publications. Feasibility counts for road traffic injuries are based on the November 2022 CPRD Gold primary care database.

Table 1: Estimated Sample Sizes of Patients in England Eligible for Linkage to HES datasets

**Outcome**	**Data Source**	**Average cases per year**	**Average cases per week per year**	**Average cases in 8 week period per year***	**Cases in 8 week period for our 15 year study**
Road traffic injuries (all cases)	CPRD Gold**	5,275	101	812	12,174
Cardiovascular disease*** (incident only)	Gho et al (2018), Wu et al. (2022), Yang et al. (2021), Lawson et al. (2021)[15-18]	19,489	375	2998	44,974
Depression (all cases)	Kendrick et al. (2015)[19]****	22,020	423	3388	50,815

*We are looking at the 8 week period of 4 weeks before & after each clock change.

**Identified using CPRD Gold medical code 279: Motor vehicle traffic accidents (MVTA). These estimates do not include HES and are likely to be conservative.

***Myocardial infarction, atrial fibrillation, stroke & heart failure.

****Counts are based on 75% of the counts in the original paper which used all patients in England rather than just those with HES linkage (75% of English practices have HES linkage).

Sample size considerations

The minimum detectable effect size for our primary outcomes at 80% power was calculated in Stata using the power twoproportions function.

Table 2: Calculations to Estimate Minimum Detectable Effect Size at 80% Power

**Outcome**	**Total underlying population from existing literature***	**Cases in 4 week pre-exposure period over 15 year study**	**Prevalence (unexposed)****	**Assumed Prevalence exposed*****	**Minimum detectable effect size (percent increase between exposed and unexposed) at 80% power**
Road traffic injuries	3,383,148	6,090	0.001800099	0.001931686	7.31%
Cardiovascular disease	3,383,148	22,485	0.006646177	0.006896073	3.76%
Depression	3,383,148	25,410	0.007510756	0.007776637	3.54%

*From Wu et al (2022) Patients in England aged ≥16 years, contributing data 1998-2017,eligible for HES linkage.[16]

**15-year 4-weekly cases/total population

***(Unexposed prevalence * (effect size detectable/100)) + unexposed prevalence

Planned use of linked data (if applicable):

The following linkages will be requested:

HES Admitted Patient Care (HES-APC)

HES A&E

Index of Multiple Deprivation (IMD), patient level

Linked HES data will be important to gain full data on our outcomes, many of which will be more likely to be captured in secondary care data. Linked CPRD and HES data will be used to identify prevalent and incident cases for subgroup analyses exploring the effects of DST clock changes on prevalent and incident cases separately (where appropriate). Patient Level Index of Multiple Deprivation data will be used to identify whether socioeconomic position is a modifier of the effect of DST clock changes on our health outcomes.

A secondary use of the linked data will be to identify significant subsequent health events (hospitalisations/referrals for depression/cardiovascular disease, or deaths) amongst those visiting primary/secondary care for depression/cardiovascular disease.

Definition of the Study population

a) Target population: The population of England (all ages for road traffic accidents) (aged ≥10 for depression, anxiety, self-harm, eating disorders and sleep disorders) (aged ≥40 for cardiovascular disease).

b) Recruitment period & start/end of follow-up: 2008 – 2022

Recruitment starts in 2008 as all the datasets we are using are available from then onwards (first full year of HES A&E data is 2008).

c) Inclusion criteria (based on clinical data):

1. All patients in England, eligible for data linkage, registered in the CPRD GOLD database in the 4 weeks before and after each DST clock change.

2. All ages for road traffic injuries.

Aged ≥10 for depression, sleep disorders, anxiety, self-harm and eating disorders.

Aged ≥40 for cardiovascular disease.

3. Clinical record with at least 12 consecutive months of records classified as ‘acceptable’ by the CPRD from all “up to standard” practices.

In the UK the clocks go forward 1 hour at 1am on the last Sunday in March and go back 1 hour at 2am on the last Sunday in October. Consequently, the clock changes do not occur on the same date each year. For a full list of clock change dates by year see Appendix A.

Exclusion criteria:

Patients aged <10 years for depression, anxiety, eating disorders, self-harm and sleep disorders

Patients aged <40 for cardiovascular disease

For analysis of significant subsequent health events: those with data not up to standard during the follow-up period.

d) Index date: Event date (date of visit)

e) Incident case: First ever record for this disease code. Prevalent case: Has record for this disease code prior to event date.

f) Sampling from base population: N/A.

g) Exposure windows: Exposed = The 4 weeks after the clock changes.

Unexposed = The 4 weeks before the clock changes.

h) Linked data will be subject to the same eligibility criteria.

Selection of comparison group(s) or controls

In this study we are comparing our exposed group (those visiting primary/secondary care after the clock change) to our unexposed group (those visiting primary/secondary care before the clock change)

Figure 1: Diagram to Show Unexposed and Exposed Periods

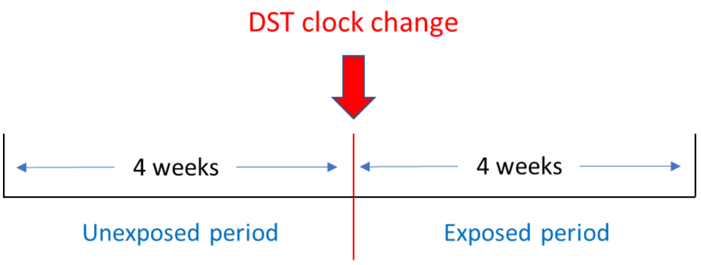

Exposures, Outcomes and Covariates

Exposure periods:

Unexposed: 4 weeks before the clock change.

Exposed: 4 weeks after the clock change.

Primary outcomes

All primary/secondary care (incident & prevalent cases) visits in the 1- (modelled daily), 2- and 4-week periods before and after the clock changes, stratified by age, for:

Depression (aged ≥10).

Road traffic injuries (all ages).

Cardiovascular disease (aged ≥40*.

*The age at which people become eligible for their NHS health check and are considered at higher risk of cardiovascular disease.

Secondary outcomes

All primary/secondary care visits (incident & prevalent) in the 1- (modelled daily), 2- and 4-week periods before and after the clock changes, aged ≥10, stratified by age, for:

Sleep disorders. This analysis will be exploratory, and will only be conducted if it is possible to adequately define sleep disorders within CPRD data.

Other mental health (anxiety & self-harm) & eating disorders.

The number of subsequent hospitalisations/referrals for depression/cardiovascular disease, or deaths amongst those visiting primary/secondary care for depression/cardiovascular disease in the 1-, 2- and 4-week periods before and after the clock changes.

Definition of outcomes

Cardiovascular disease, road traffic injuries, self-harm and eating disorders will be defined using ICD10, Read codes and HES A&E codes.

Depression and anxiety will be defined using ICD-10, Read codes and HES A&E codes.

Read codes alone will be used to define depression/anxiety if the code is specific enough (see code lists C1 and C6 for depression/anxiety diagnoses).

However, where Read codes relate to symptoms of depression/anxiety (see code lists C2 and C7 for depression/anxiety symptoms) that are not considered adequate to define depression/anxiety, patients will only be counted as having depression/anxiety if they have been prescribed a drug used to treat depression within 90 days.

Sleep disorders will be defined using ICD-10 and Read codes.

Sleep disorders are difficult to define which means that most Read codes are not specific and relate to symptoms rather than diagnoses. Patients will therefore only counted as having a sleep disorder if they had a Read code for a sleep disorder diagnosis or symptom and were prescribed a drug used to treat sleep disorders within 90 days.

*Note to reviewer:

Depression, anxiety and sleep disorders will not be defined based on prescription alone in order to avoid

Data/ Statistical Analysis

We will use a regression discontinuity design to investigate whether the mean number of primary/secondary care visits for depression, cardiovascular disease, road traffic injuries, anxiety, eating disorders or self-harm (and sleep disorders if possible), differs between the 4 weeks before and after the clock changes.

Data for spring and autumn clock changes will be analysed separately, as will the data for each type of diagnosis (depression, cardiovascular disease, road traffic injuries, anxiety, eating disorders, self-harm and sleep disorders).

As well as the whole 4-week study period, differences in visits in the 1-week (modelled daily) and 2-week periods before and after the clock changes will be compared.

The same analysis plan will be used to examine differences in the number of subsequent hospitalisations/referrals for depression/cardiovascular disease, or deaths amongst those visiting primary/secondary care for depression/cardiovascular disease before and after the clock changes.

We will also conduct stratified subgroup analysis to explore whether the effects of the clock changes are modified by factors such as year, incident vs. prevalent case, sex, age, ethnicity, socioeconomic position, alcohol, smoking, BMI, blood pressure, co-morbidities and disease subgroups.

To investigate whether differences observed represent a true discontinuity rather than seasonal patterns, mean daily visits for the whole period of the 4 weeks before and after the clock changes will be plotted. Meta-regression will then be used to quantify whether the greatest discontinuity occurs at the time of the clock changes.

Sensitivity analyses excluding certain years will be conducted to address the potential confounding effects of Easter Sunday and COVID lockdowns.

Analyses will be adjusted for the fact that the Sunday following the clock changes has more or less hours than other days. The shortening of the Sunday after transition into DST will be adjusted for by multiplying the number of primary/secondary care visits by 24/23, and the lengthening of the Sunday after transition out of DST by multiplying by 24/25.

As a sensitivity analysis we will explore different methods of bandwidth selection using appropriate packages in stata[20]. We will also build in a sensitivity analysis with a different cut-off point to the clock change.

Our study is hypothesis driven and we only have 3 primary outcomes. In addition, we will focus on interpreting the effect sizes and confidence intervals rather than just p values. However, where appropriate, we will account for multiple testing when interpreting p-values, for example via False Discovery Rate adjustment or a Bonferroni correction.

*Notes to reviewer:

We are using individual level data to do our regression discontinuity analysis. Using individuals as their own controls would be tricky as once someone has had an outcome (e.g. heart attack / going to GP about depression) this makes them less likely to have the same outcome again during the study period.

Plan for addressing confounding

There are several potential confounders of the relationship between sleep and circadian rhythms before and after the clock changes, and the number of primary/secondary care visits:

Figure 2: DAG to Show Relationships between Exposure, Outcome and Confounders

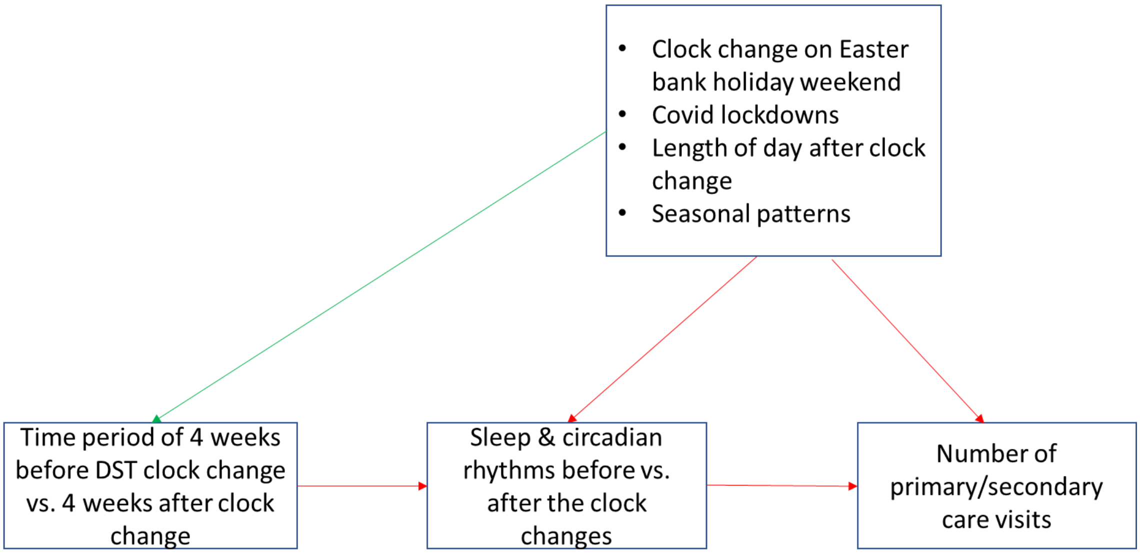

Easter – The DST clock change falls on Easter Sunday twice during our study period of 2007-2022 (See Appendix A). This could confound the acute effects of the clock change as Easter Monday is a bank holiday so people do not need to get up for work. Consequently the effects of the clock change on sleep may be delayed by a day. People are also less likely to access healthcare services on a bank holiday. To explore whether this is an issue we will conduct a sensitivity analysis excluding these years.

Length of day – The absolute number of diagnoses on the Sunday of the clock change may be confounded by the difference in the length of this day compared to all other days (-1 in spring and +1 in autumn). This will be adjusted for as described above.

Covid 19 lockdowns in 2020/21 - In 2020 the spring DST clock change was 29th March. On 19th March people ordered to stay at home & on 23rd March lockdown measures legally came into force. The 2020 Autumn clock change was 25th October. New restrictions were introduced on the 22nd September and on the 5th November the second national lockdown came into force in England. These lockdowns and restrictions may have affected people's sleep (not needing to travel to work could increase sleep, stress from the situation could reduce sleep). It could also affect our health outcomes by reducing people's access to healthcare services, as well as directly affecting rates of some outcomes such as of depression and cardiovascular disease risk (due to stress). To explore whether COVID lockdowns have affected our results we will conduct a sensitivity analysis excluding these years.

Seasonal effects - It is possible that depression increases after the autumn clock change regardless of the clock change because it is getting colder and darker (and vice versa in spring). To investigate this, mean daily visits surrounding both spring and autumn clock changes will be plotted separately to assess whether patterns show a true discontinuity or are purely seasonal trend.

Common individual level confounders (e.g. age, sex etc.) shouldn't apply in this analysis as, although potentially associated with our outcomes, they are not associated with our exposure (sleep and circadian rhythms in the period before versus after the clock changes). They will therefore act as effect modifiers in this analysis and be examined through stratified subgroup analysis.

Plans for addressing missing data<br><br>We will investigate the extent and randomness of missing data in our exposure, outcomes and effect modifiers, and use multiple imputation to impute missing data where appropriate. Missing data is likely to be a particular issue for effect modifiers such as alcohol intake, BMI, smoking and blood pressure which are known to not be available for all patients and may not be missing at random.
Patient or user group involvement<br><br>No patients or user groups were involved in the drafting of this protocol. However, depending on our findings, we will work with Bristol's Integrative Epidemiology Unit Public Engagement Department (www.bristol.ac.uk/integrative-epidemiology/engagement/) which engages with members of the public to share research and benefit from others' insights. The department encourages discussion about research methods and findings through a programme of activities in partnership with festivals, schools and community groups. This will enable me to engage the public, patients and policy makers in the interpretation and dissemination of our results, as well as discussing ideas for future research (see also Plans for disseminating study results section).
Plans for disseminating and communicating study results<br><br>This study will form the core of a funded 3-year PhD project. We will publish the results of the study as open-access peer-reviewed publications. Our results will be disseminated in up to six papers as detailed below:<br><br>Analysis plan for Estimating the Effects of 'Daylight Saving Time' Clock Changes on Health Outcomes in England.<br><br>The effect of daylight savings time transitions on depression in England.<br><br>The effect of daylight savings time transitions on cardiovascular disease in England.<br><br>The effect of daylight savings time transitions on road traffic injuries in England.<br><br>The effect of daylight savings time transitions on anxiety, eating disorders and self-harm in England.<br><br>The effect of daylight savings time transitions on sleep disorders in England (if possible).<br><br>All code will be made freely available on GitHub. We will disseminate findings through national and international conferences as are appropriate. Depending on the findings we will also explore additional options, such as media press releases and blog posts on IEUREKA (https://ieureka.blogs.bristol.ac.uk/), for wider dissemination to the public and policy makers. I will also work with PolicyBristol (www.bristol.ac.uk/policybristol) to specifically engage with policy makers and translate my research findings into clear and accessible policy briefings.
Conflict of interest statement<br><br>N/A

Limitations of the study design, data sources, and analytic methods

This study has several limitations:

This is an observational study. We will attempt to control for confounders and identify effect modifiers via, adjusting, stratified subgroup analysis and secondary analyses excluding certain years. However, any differences observed before and after the clock changes could still have arisen due to uncontrolled confounding. That said, there are unlikely to be any major individual level confounders, because DST is unlikely to be related to most individual level factors. Furthermore, we will exploit random variation in when in the year the DST change occurs to investigate this further.

GP and hospital data will only capture the most severe cases so more subtle effects of the clock changes won't be captured in this study. However, most substantial health consequences are likely to be recorded.

There may be a delay in patients seeking and then getting medical help for non-urgent conditions such as sleep disorders and depression. This may lead to some misclassification whereby people diagnosed after the clock change were already experiencing symptoms prior to the clock change. We will evaluate this using sensitivity analyses, for example, using a fuzzy regression discontinuity design[21].

Data (such as smoking status and BMI) are unlikely to be missing at random in healthcare data. This may make it challenging to use multiple imputation. We will run sensitivity analyses to investigate this, for example comparing the results from a complete case analysis with an analysis assuming those with missing BMI/smoking are normal weight/never smokers.

References

Hansen, B. T., Sønderskov, K. M., Hageman, I., Dinesen, P. T. & Østergaard, S. D. (2017) Daylight Savings Time Transitions and the Incidence Rate of Unipolar Depressive Episodes. *Epidemiology*, 28(3), pp. 346-353. https://doi.org/10.1097/EDE.0000000000000580

Malow, B. A., Veatch O. J. & Bagai, K. (2019) Are Daylight Saving Time Changes Bad for the Brain? *JAMA Neurology*, 77(1), pp. 9–10. https://doi.org/10.1001/jamaneurol.2019.3780

Roenneberg, T., Wirz-Justice, A., Skene, D. J., Ancoli-Israel, S., Wright Jr., K. P., Dijk, D-J., Zee, P., Gorman, M. R., Winnebeck, E. C. & Klerman, E. B. (2019) Why Should We Abolish Daylight Saving Time? *Journal of Biological Rhythms*, 34(3), pp. 227–230. https://doi.org/10.1177/0748730419854197

Zhang, H. X., Dahlen, T., Khan, A., Edgren, G. & Rzhetsky, A. (2020) Measurable Health Effects Associated with the Daylight Saving Time Shift. *Plos Computational Biology*, 16(6), e1007927. https://doi.org/10.1371/journal.pcbi.1007927

Kantermann, T., Juda, M., Merrow, M. & Roenneberg, T. (2007) The Human Circadian Clock's Seasonal Adjustment Is Disrupted by Daylight Saving Time. *Current Biology*, 17(22), pp. 1996-2000. https://doi.org/10.1016/j.cub.2007.10.025.

Jiddou, M. R., Pica, M., Boura, J., Qu, L. & Franklin, B. A. (2013) Incidence of Myocardial Infarction With Shifts to and From Daylight Savings Time. *The American Journal of Cardiology*, 111(55), pp. 631-635. https://doi.org/10.1016/j.amjcard.2012.11.010

Chudow, J. J., Dreyfus, I., Zaremski, L., Mazori, A. Y., Fisher, J. D., Di Biase, L., Romero, J., Ferrick, K. J. & Krumerman, A. (2020) Changes in Atrial Fibrillation Admissions Following Daylight Saving Time Transitions. *Sleep Medicine*, 69, pp. 155-158. https://doi.org/10.1016/j.sleep.2020.01.018

Sipila, J. O. T., Ruuskanen, J. O., Rautava, P. & Kytö, V. (2016) Changes in Ischemic Stroke Occurrence Following Daylight Saving Time Transitions. *Sleep Medicine*, 27-28, pp. 20-24. https://doi.org/10.1016/j.sleep.2016.10.009.

Fritz, J., VoPham, T., Wright K. P. Jr. & Vetter, C. (2020) A Chronobiological Evaluation of the Acute Effects of Daylight Saving Time on Traffic Accident Risk. *Current Biology*, 30(4), pp. 729-735.e2. https://doi.org/10.1016/j.cub.2019.12.045

Rishi, M. A., Ahmed, O., Barrantes Perez, J. H. et al. (2020) Daylight Saving Time: An American Academy of Sleep Medicine Position Statement. *Journal of Clinical Sleep Medicine*, 16(10), pp. 1781–1784. https://doi.org/10.5664/jcsm.8780

European Parliament (2019). Could Switching Between Summer and Winter Time End in 2021? *European Parliament Press Release*, April 3, 2019. Available at: https://www.europarl.europa.eu/pdfs/news/expert/2019/3/press_release/20190304IPR30073/20190304IPR30073_en.pdf.

Manfredini, R., Fabbian, F., De Giorgi, A., Zucchi, B., Cappadona, R., Signani, F., Katsiki, N. & Mikhailidis, D. P. (2018) Daylight Saving Time and Myocardial Infarction: Should We Be Worried? A Review of the Evidence. *European Review for Medical and Pharmacological Sciences*, 22(3), pp. 750-755. https://doi.org/10.26355/eurrev_201802_14306

Venkataramani, A. S., Jacob, B. & Jena, A. B. (2016) Regression Discontinuity Designs in Healthcare Research, *British Medical Journal*, 352, i1216. https://doi.org/10.1136/bmj.i1216

Chaplin, D. D, Cook, T. D., Zurovac, J., Coopersmith, J. S., Finucane, M. M., Vollmer, L. N., Morris, R. E. (2018). The Internal and External Validity of the Regression Discontinuity Design: A Meta-Analysis of 15 Within-Study Comparisons. *Journal of Policy Analysis and Management*. 37 (2): 403–429.

Gho, J. M. I. H., et al. (2018) An Electronic Health Records Cohort Study on Heart Failure Following Myocardial Infarction in England: Incidence and Predictors. *BMJ Open* 8 (3): e018331.

Wu, J., et al. (2022) Temporal Trends and Patterns in Atrial Fibrillation Incidence: A Population-Based Study of 3·4 Million Individuals. *The Lancet Regional Health – Europe,* 17: 100386.

Yang, Z., et al. (2022) Association of Major Blood Lipids with Post-Stroke Dementia: A Community-Based Cohort Study. *European Journal of Neurology,* 29 (4): 968-979.

Lawson, C. A., et al. (2021) Outcome Trends in People with Heart Failure, Type 2 Diabetes Mellitus and Chronic Kidney Disease in the UK Over Twenty Years. *EClinicalMedicine* [illegible]

List of Appendices

Appendix A: List of Dates of DST Clock Changes 2008-2022

Appendix B: ICD10 Codes

Appendix B1: Depression ICD10 Codes

Appendix B2: Road Traffic Injuries ICD10 Codes

Appendix B3: Cardiovascular Disease ICD10 Codes

Appendix B4: Sleep Disorders ICD10 Codes

Appendix B5: Anxiety ICD10 Codes

Appendix B6: Self-Harm ICD10 Codes

Appendix B7: Eating Disorders ICD10 Codes

Appendix C: Read v2 codes

Appendix C1: Depression Diagnosis Read Codes

Appendix C2: Depression Symptom Read Codes

Appendix C3: Road Traffic Injuries Read Codes

Appendix C4: Cardiovascular Disease Read Codes

Appendix C5: Sleep Disorders Read Codes

Appendix C6: Anxiety Diagnosis Read Codes

Appendix C7: Anxiety Symptom Read Codes

Appendix C8: Self-Harm Read Codes

Appendix C9: Eating Disorders Read Codes

Appendix D:Prescription codes

Appendix D1: Depression Prescription Product Codes

Appendix D2: Anxiety Prescription Product Codes

Appendix D3 : Sleep Disorders Prescription Product Codes

Appendix E: HES A&E codes

Appendix F: Tables & Diagrams

Appendix F1: Feasibility Counts

Appendix F2: Sample Size Considerations

Appendix F3: Comparison Groups

Appendix F4: Confounding

Grant ID (optional)

Text S2: Minor deviations from protocol

The linked HES A&E data provided by CPRD only contained data up to March 2020. We therefore reduced our study period from 2008-2022 to 2008-2019. This also removed the need to address the effects of covid lockdowns in our study.

Once we started analysing our data we discovered that there were patients who had more than one event for the same health condition within the 8-week period surrounding an individual clock change. Whilst this was not common, we decided to examine whether this was affecting our results by running the two sensitivity analyses described in the main paper.

We did not run stratified analyses by year, marital status, disease comorbidities or ethnicity as there were no prior indications that these would act as effect modifiers and because the sample sizes of the individual strata (and subsequently statistical power) would have been low. In addition ethnicity is poorly recorded in patients registered prior to 2006[1].

We originally planned to conduct a sensitivity analysis excluding years where the clock change coincided with Easter Sunday in order to examine the potential confounding effects of Easter Sunday on the relationship between the clock changes and the number of health events. However, instead we decided to adjust all of our Spring regression analyses using an indicator variable for the five days of the Easter weekend. This maximised our sample size, allowed us to take the whole long weekend into account and considered the effects of Easter wherever it fell within our 8-week study periods around the Spring clock change.

In our analysis we split the data by day, year and region to account for all sources of variability in the data.

Because the effect sizes we found in our study were small we did not go on to explore the number of subsequent hospitalisations/referrals for depression/cardiovascular disease, or deaths amongst those visiting primary/secondary care for depression/cardiovascular disease in the 1-, 2- and 4-week periods before and after the clock changes.

Text S3: Definitions of outcomes

Search and selection of disease and prescription codes

Medical codes (symptoms/diagnoses) and product codes (prescriptions) were used to identify events in primary care. ICD-10 codes (symptoms/diagnoses) were used to identify events in Hospital Episode Statistics Admitted Patient Care, and A&E codes were used to identify events in Hospital Episode Statistics Accident and Emergency data.

Where available, existing published code lists were used as a starting point for my code lists. Where previous code lists did not exist (e.g. road traffic injuries (ICD-10 & medical codes), sleep (ICD-10 codes)), these were created by searching the ICD-10 dictionary (International Classification of Diseases, 10th revision) or the CPRD GOLD medical dictionary using keywords. All accident and emergency (A&E) code lists were created from scratch using the dataset's unique coding system. All code lists were checked by a general practitioner experienced in using the codes in clinical practice and refined following their feedback.

For the eight health conditions, codes not relevant to a current diagnosis or symptom (e.g. condition in remission, history of condition) were excluded, as were codes where the timing of the code was not considered to be directly related to the onset of disease (e.g. monitoring/management plans/referrals/advice/counselling). Codes were also excluded if they represented tests (e.g. imaging or sleep/mood rating scales) as they only show a test has been carried out, not that a diagnosis was confirmed.

The HES Accident & Emergency dataset has its own coding system. Road traffic injuries and self-harm were able to be identified on the basis of the 'aepatgroup' code which gives the reason for the A&E episode. CVD events were identified on the basis of the 'aepatgroup' combined with a 2 digit diagnosis code. It was not possible to specify individual codes for depression, anxiety, eating disorders or sleep disorders. Instead we included the broad category of 'psychiatric conditions' in A&E only as a separate condition.

Anxiety

Anxiety events in primary and secondary care were defined using ICD-10 and medical codes. ICD-10 codes alone were used to define anxiety. Medical codes alone were used to define anxiety if the code was specific enough. However, where medical codes related to symptoms of anxiety that were not considered adequate to define anxiety, patients were only counted as having anxiety if they were prescribed a drug used to treat anxiety within 90 days either side of the medical code.

ICD-10 codes for the following conditions were included/excluded from our definition:

Included	Excluded
Phobic anxiety disorders<br>Other anxiety disorders*<br>Obsessive-compulsive disorder<br>Reaction to severe stress, and adjustment disorders<br>Dissociative [conversion] disorders<br>Other neurotic disorders	Specific (isolated) phobias (e.g. heights)<br>Somatoform phobias

*This includes mixed anxiety & depressive disorder which is included in the definitions of both depression and anxiety.

Medical codes for the following conditions (diagnoses & symptoms) were included/excluded from our definition:

**Included**	**Excluded**
Generalised anxiety disorder<br>Panic disorder<br>Agoraphobia<br>Social anxiety disorder<br>Mixed anxiety and depression*<br>Obsessive compulsive disorders **<br>Trauma- and stress-related disorders** with anxiety, including PTSD, acute stress disorder, and adjustment disorder with anxiety	Specific phobias (e.g. heights)<br>Somatic symptoms disorder

*Mixed anxiety & depressive disorder is included in the definitions of both depression and anxiety.

**Prior to the publication of DSM-5 in 2013, obsessive-compulsive and stress-related disorders were classified as anxiety disorders. In a major change, DSM-5 classified these conditions separately from anxiety disorders. This study uses patient data from 2008-2019. It is not clear how, or whether, the change affected the allocation of Read codes by GPs. The accuracy of the medical codes to identify these conditions may therefore be suboptimal.

Drugs used to treat anxiety:

**Substance name**
Alprazolam
Amitriptyline hydrochloride/ Chlordiazepoxide
Buspirone hydrochloride
Chlordiazepoxide hydrochloride
Diazepam
Duloxetine hydrochloride
Escitalopram oxalate
Lorazepam
Meprobamate
Moclobemide
Oxazepam
Oxprenolol hydrochloride
Paroxetine hydrochloride
Pericyazine
Perphenazine
Pregabalin
Trazodone hydrochloride
Venlafaxine hydrochloride

Cardiovascular disease (major, acute)

Major, acute cardiovascular disease events in primary and secondary care were defined with medical codes, ICD-10 and A&E codes.

ICD-10 and medical codes (diagnoses & symptoms) for the following conditions were included/excluded from our definition:

Included	Excluded
Unstable angina/acute coronary syndrome<br>Myocardial infarction*<br>Acute ischaemic heart disease<br>Heart failure<br>Cardiac arrest<br>Atrial fibrillation & arrhythmias<br>Stroke<br>Transient ischaemic attack	

*Codes for events following or subsequent to MI have been included as there is thought to be a high likelihood of miscoding with these MI codes which could lead to MIs being missed.

To be included as an A&E case of major, acute cardiovascular disease events had to have one of the following ‘aepatgroup’ codes (the reason for the A&E visit) and one of the following ‘diag2’ codes (diagnosis codes):

aepatgroup	diag2
Brought in dead<br>Other than above<br>Not known	Cardiac conditions<br>Cerebro-vascular conditions<br>Other vascular conditions

Depression

Depression events were defined in primary and secondary care using ICD-10 and medical codes. ICD-10 codes alone were used to define depression. Medical codes alone were used to define depression if the code was specific enough. However, where medical codes related to symptoms of depression that were not considered adequate to define depression, patients were only counted as having depression if they were also prescribed a drug used to treat depression within 90 days either side of the medical code.

ICD-10 codes for the following conditions were included/excluded from our definition:

Included	Excluded
Depressive episode<br>Recurrent depressive disorder<br>Persistent mood [affective] disorders<br>Mixed anxiety and depressive disorder*<br>Depressive conduct disorder	

*Mixed anxiety & depressive disorder is included in the definitions of both depression and anxiety.

Medical codes for the following conditions (diagnoses & symptoms) were included/excluded from our definition:

Included	Excluded
Major depressive disorder<br>Dysthymia<br>Recurrent depressive disorder<br>Mixed anxiety and depression*<br>Disruptive mood dysregulation disorder<br>Depression in dementia (or other condition)<br>Trauma- and stress-related disorders with depressed mood (including adjustment disorders with depressed mood**)	Bipolar and related disorders (including bipolar I, II and cyclothymic disorder)<br>Premenstrual dysphoric disorder<br>Suicide***<br>Self-harm***<br>Maternal depression

*Mixed anxiety & depressive disorder is included in the definitions of both depression and anxiety.

**Symptoms and treatment of adjustment disorders overlap with both depression and anxiety. To avoid misclassification, adjustment disorders will be included within our definitions of both depression and anxiety. Also see note on stress-related disorders on anxiety table.

***Suicide and self-harm are examined as a separate condition to depression.

Drugs used to treat depression:

Substance name
Agomelatine
Amitriptyline Hydrochloride
Amitriptyline Hydrochloride/ Perphenazine
Citalopram hydrobromide
Citalopram hydrochloride
Clomipramine hydrochloride
Dosulepin hydrochloride
Dosulepin Hydrochloride
Duloxetine hydrochloride
Escitalopram oxalate
Fluoxetine hydrochloride
Fluvoxamine maleate
Imipramine hydrochloride
Isocarboxazid
Lofepramine hydrochloride
Mianserin hydrochloride
Mirtazapine
Moclobemide
Nortriptyline hydrochloride
Nortriptyline Hydrochloride
Paroxetine hydrochloride
Phenelzine sulfate
Reboxetine mesilate
Sertraline
Sertraline hydrochloride
Tranylcypromine sulfate
Trazodone Hydrochloride
Trimipramine maleate
Venlafaxine hydrochloride
Venlafaxine Hydrochloride

Substance name
Vortioxetine hydrobromide

Eating disorders

Eating disorder events in primary and secondary care were defined with ICD-10 and medical codes. Because we are looking at the effect of the clock changes (via sleep) on eating disorders we excluded organic conditions which are caused by an underlying medical condition rather than having a psychiatric cause.

ICD-10 codes for the following conditions were included/excluded from our definition:

Included	Excluded
Anorexia nervosa (including atypical)<br>Bulimia nervosa (including atypical)<br>Overeating/vomiting associated with psychological disturbances<br>Unspecified/other eating disorder	

Medical codes for the following conditions were included/excluded from our definition:

Included (diagnoses & symptoms)	Excluded
Anorexia nervosa (including atypical)<br>Bulimia nervosa<br>Compulsive eating disorder<br>Non-organic* eating disorders (specified or unspecified)<br>Hyperorexia nervosa<br>Overeating associated with psychological disturbances/ psychogenic overeating<br>Other eating disorders (including unspecified)<br>Nocturnal sleep-related eating disorder	Organic eating disorders (without an overt psychiatric cause) e.g. hyperalimentation, pica, starvation, anorexia (without nervosa suffix), excessive eating/polyphagia without psychological disturbance.

*Nonorganic eating disorders are those where emotional/psychiatric causes are believed to be the main contributory factor rather than organic eating disorders which are caused by an underlying physical medical condition.

Psychiatric conditions

It was not possible to specify individual codes for anxiety, depression, eating disorders or sleep disorders in the A&E data. Instead we examined the broader condition of 'psychiatric conditions'. To be included as a psychiatric condition in A&E, events had to have one of the following 'aepatgroup' codes (the reason for the A&E visit) and the following 'diag2' code (diagnosis code):

aepatgroup	diag2
Brought in dead<br>Other than above<br>Not known	Psychiatric conditions

Road traffic injuries

Road traffic injury events in primary and secondary care were defined with medical codes, ICD-10 and A&E codes.

ICD-10 codes for the following conditions were included/excluded from our definition:

Included	Excluded
Land traffic accident involving a motor vehicle<br><br>Unspecified / other specified land transport accident<br><br>Unspecified vehicle land transport accident<br><br>Crashing of motor vehicle, undetermined intent	Land transport accidents specifically reported as 'non-traffic'.<br><br>Accidents specifically reporting not involving motor vehicles (e.g. pedestrian hit by pedal bike/train)<br><br>Accidents to persons engaged in the maintenance or repair of transport equipment or vehicle (not in motion) unless injured by another vehicle in motion<br><br>Accidents involving vehicles, but unrelated to the hazards associated with the means of transportation, e.g., injuries received in a fight on board ship; transport vehicle involved in a cataclysm; finger crushed when shutting car door<br><br>Intentional self-harm*

*Suicide and self-harm are examined as a separate condition to road traffic injuries.

Medical codes for the following conditions were included/excluded from our definition:

Included	Excluded
Traffic accidents involving motor vehicles (including MVTA*)<br>Crashing of motor vehicle undetermined intent<br>Road vehicle accidents not otherwise specified<br>Other road vehicle accidents<br>RTA – road traffic & other transport accidents<br>Accidents involving vehicles not elsewhere classifiable/not otherwise specified<br>Vehicle accident not elsewhere classifiable/not otherwise specified	Transport accidents that specifically report being 'non-traffic' (including (MVNTA**)<br><br>Accidents that specifically report not involving motor vehicles (e.g. pedestrian hit by non-motor vehicle or pedal bike)<br><br>Intentional self-harm/ suicide/jumping in front of vehicle/exhaust gas poisoning***

*MVTA = motor vehicle traffic accident
**MVNTA = motor vehicle non-traffic accident
***Suicide and self-harm are examined as a separate condition to road traffic injuries.

To be included as a road traffic injury in A&E, events had to have the following ‘aepatgroup’ code (the reason for the A&E visit):

aepatgroup	
Road traffic accident	

Self-harm

Intentional self-harm events in primary and secondary care were defined using ICD-10, medical codes and A&E codes.

ICD-10 codes for the following conditions were included/excluded from our definition:

Included	Excluded
Intentional self-harm<br>Sequelae of intentional self-harm	Events of undetermined intent<br>Sequelae of events undetermined intent

Medical codes for the following conditions (diagnoses & symptoms) were included/excluded from our definition:

Included	Excluded
Suicide/attempted suicide<br>Self-harm (intentional, self-inflected injury, poisoning, overdose through any means)	Events of undetermined intent<br>Accidental injury/self-harm<br>Events undetermined whether self-inflicted

To be included as self-harm A&E, events had to have the following ‘aepatgroup’ code (the reason for the A&E visit):

aepatgroup	
Deliberate self-harm	

Sleep disorders

Sleep disorder events in primary and secondary care were defined using ICD-10 and medical codes. ICD-10 codes alone were used to define sleep disorders. Sleep disorders are difficult to define which means that most Read codes are not specific and relate to symptoms rather than diagnoses. Patients were therefore only counted as having a sleep disorder if they had a Read code for a sleep disorder diagnosis or symptom and were also prescribed a drug used to treat sleep disorders within 90 days either side of the medical code.

ICD-10 codes for the following conditions were included/excluded from our definition:

Included	Excluded
Disorders of initiating and maintaining sleep [insomnias]<br>Disorders of excessive somnolence [hypersomnias]<br>Disorders of the sleep-wake schedule<br>Other/unspecified sleep disorders/disorders<br>Nonorganic* insomnia/hypersomnia/disorders of sleep-wake schedule<br>Sleep walking/ sleep terrors/nightmares<br>Other/unspecified nonorganic sleep disorders	Sleep apnoea<br>Narcolepsy<br>Cataplexy

*Nonorganic sleep disorders are those where emotional causes are believed to be the main contributory factor rather than an underlying physical medical condition.

Medical codes for the following conditions (diagnoses & symptoms) were included/excluded from our definition:

Included	Excluded
Insomnia<br>Hypersomnia<br>Circadian rhythm sleep-wake disorders<br>Paraomnias<br>Sleep quality/insomnia indexes	Narcolepsy<br>Breathing-related disorders (including sleep apnoea)<br>Cataplexy

Drug substances used to treat sleep disorders:

Included	Excluded*
Temazepam	Promethazine hydrochloride
Diazepam	Promethazine hydrochloride/pholcodine
Zopiclone	Pethidine hydrochloride/promethazine hydrochloride
Clomethiazole	Paracetamol/promethazine hydrochloride/ dextromethorphan hydrobromide
Zolpidem tartrate	Dextromethorphan hydrobromide/promethazine hydrochloride/ paracetamol
Clomethiazole edisilate	
Flunitrazepam	
Flurazepam hydrochloride	
Lormetazepam	
Oxazepam	
Loprazolam mesilate	
Melatonin	
Nitrazepam	

*We excluded antihistamines, cough syrups and cold & flu relief products that are unlikely to be prescribed for a sleep disorder.

Text S4: Creation of code lists from scratch

ICD10 code lists

There were no existing ICD10 code lists for road traffic injuries, eating disorders or sleep disorders so these were created by manually searching the ICD-10 hierarchy (https://icd.who.int/browse10/2016/en).

Medical code lists

There were no existing medical code lists for road traffic injuries. Instead this code list was created by searching descriptions in the CPRD gold medical dictionary (June 2022 build) for the following terms: "*motor*" "*traffic*" "*mvta*" "*vehicle*" "*road*" "*collision*" "*rta*" "*rtc*. Codes not related to road traffic injuries were removed.

Text S5: CODE-EHR Checklist

CODE-EHR framework: Best practice checklist to report on the use of structured electronic healthcare records in clinical research

Date of completion: 11/Apr/25 Study name: The Effects of Daylight Saving Time Clock Changes on Health in England: A Population Based Retrospective Cohort Study Using Linked Electronic Health Records

Item	Objective	Framework standards	Minimum information to provide	Lead Author acknowledgement
1. Dataset construction and linkage	To provide an understanding of how the structured healthcare data were identified and used.	Minimum: Flow diagram of datasets used in the study, and description of the processes and directionality of any linkage performed, published within the research report or supplementary documents.<br>Preferred: Provided within a pre-published protocol or open-access document.	(a) State the source of any datasets used.<br>(b) Comment on how the observed and any missing data were identified and addressed, and the proportion observed for each variable.<br>(c) Provide data on completeness of follow-up.<br>(d) For linked datasets, specify how linkage was performed and the quality of linkage methods.	Select one option:<br>Minimum standard not met ☐<br>Minimum standard met ☐<br>Preferred standard met ☒
2. Data fit for purpose	To ensure transparency with the approach taken, with respect to coding of the structured healthcare data.	Minimum: Clear unambiguous statements on the process of coding in the methods section of the research report.<br>Preferred: Provided within a pre-published protocol or open-access document.	(a) Confirm origin, clinical processes, and the purpose of data.<br>(b) Specify coding systems, clinical terminologies or classification used and their versions, and any manipulation of the coded data.<br>(c) Provide detail on quality assessment for data capture.<br>(d) Outline potential sources of bias.	Select one option:<br>Minimum standard not met ☐<br>Minimum standard met ☐<br>Preferred standard met ☒
3. Disease and outcome definitions	To fully detail how conditions AND outcome events were defined, allowing other researchers to identify errors and repeat the process in other datasets.	Minimum: State what codes were used to define diseases, treatments, conditions and outcomes *prior to statistical analysis*, including those relating to patient identification, therapy, procedures, comorbidities, and components of any composite endpoints.<br>Preferred: Provided within a pre-published protocol or open-access document *prior to statistical analysis.*	(a) Detailed lists of codes used for each aspect of the study.<br>(b) Date of publication and access details for the coding manual (please add to box below).<br>(c) Provide definitions, implementation logic and validation of any phenotyping algorithms used.<br>(d) Specify any processes used to validate the coding scheme or reference to prior work.	Select one option:<br>Minimum standard not met ☐<br>Minimum standard met ☐<br>Preferred standard met ☒
4. Analysis	To fully detail how outcome events were analysed and allow independent assessment of the authenticity of study findings.	Minimum: Describe the process used to analyse study outcomes, including statistical methods and use of any machine learning or algorithmic approaches.<br>Preferred: Provide a statistical analysis plan as a supplementary file, locked *prior to analyses commencing.*	(a) Provide details on all statistical methods used.<br>(b) Provide links to any machine code or algorithms used in the analysis, preferably as open-source.<br>(c) Specify the processes of testing assumptions, assessing model fit and any internal validation.<br>(d) Specify how generalisability of results was assessed, the replication of findings in other datasets, or any external validation.	Select one option:<br>Minimum standard not met ☐<br>Minimum standard met ☐<br>Preferred standard met ☒

5. Ethics and governance	To provide patients, who may or may not have given consent, and regulatory authorities the ability to interrogate the security and provenance of the data.	Minimum: Clear unambiguous statements on how the principles of Good Clinical Practice and Data Protection will be/were met, provided in the methods section of the research report.<br>Preferred: Provided within a pre-published protocol or open-access document with evidence of patient and public engagement.	(a) State how informed consent was acquired, or governance if no patient consent.<br>(b) Specify how data privacy was protected in the collection and storage of data.<br>(c) Detail what steps were taken for patient and public involvement in the research study.<br>(d) Provide information on where anonymised source data or code can be obtained for verification and further research.	Select one option:<br>Minimum standard not met ☐<br>Minimum standard met ☒<br>Preferred standard met ☐
6. Coding manual	DOI of publication or website address: *https://github.com/MeldeLange/dst_cprd*<br>Date published: *14/May/25*			
7. Comments	Click or tap here to enter text.			
**8. Summary declaration**	One or more minimum standards not met ☐ OR **All minimum standards met** ☒<br>Number of preferred standards met: *4* / 5			

Text S6: STROBE-RECORD Statement

The RECORD statement – checklist of items, extended from the STROBE statement, that should be reported in observational studies using routinely collected health data.

	Item No.	STROBE items	Location in manuscript where items are reported	RECORD items	Location in manuscript where items are reported
**Title and abstract**					
	1	(a) Indicate the study's design with a commonly used term in the title or the abstract (b) Provide in the abstract an informative and balanced summary of what was done and what was found		RECORD 1.1: The type of data used should be specified in the title or abstract. When possible, the name of the databases used should be included.<br><br>RECORD 1.2: If applicable, the geographic region and timeframe within which the study took place should be reported in the title or abstract.<br><br>RECORD 1.3: If linkage between databases was conducted for the study, this should be clearly stated in the title or abstract.	Subtitle Title & Abstract<br><br>Title & Abstract<br><br>Abstract
**Introduction**					
Background rationale	2	Explain the scientific background and rationale for the investigation being reported	Introduction		

Objectives	3	State specific objectives, including any prespecified hypotheses	Introduction		
**Methods**					
Study Design	4	Present key elements of study design early in the paper	Abstract<br>Method: Data resource and study population		
Setting	5	Describe the setting, locations, and relevant dates, including periods of recruitment, exposure, follow-up, and data collection	Abstract.<br>Method: Data resource, study population, exposures, outcomes, covariates.		

Participants	6	*(a)* *Cohort study* - Give the eligibility criteria, and the sources and methods of selection of participants. Describe methods of follow-up *Case-control study* - Give the eligibility criteria, and the sources and methods of case ascertainment and control selection. Give the rationale for the choice of cases and controls *Cross-sectional study* - Give the eligibility criteria, and the sources and methods of selection of participants<br><br>*(b)* *Cohort study* - For matched studies, give matching criteria and number of exposed and unexposed *Case-control study* - For matched studies, give matching criteria and the number of controls per case		RECORD 6.1: The methods of study population selection (such as codes or algorithms used to identify subjects) should be listed in detail. If this is not possible, an explanation should be provided.<br><br>RECORD 6.2: Any validation studies of the codes or algorithms used to select the population should be referenced. If validation was conducted for this study and not published elsewhere, detailed methods and results should be provided.<br><br>RECORD 6.3: If the study involved linkage of databases, consider use of a flow diagram or other graphical display to demonstrate the data linkage process, including the number of individuals with linked data at each stage.	Method, paragraph 3 & Figure 1. Supplementary text S3 & S4. Code lists on Github repository.<br><br>N/A<br><br>Method, Study population
Variables	7	Clearly define all outcomes, exposures, predictors, potential confounders, and effect modifiers. Give diagnostic criteria, if applicable.		RECORD 7.1: A complete list of codes and algorithms used to classify exposures, outcomes, confounders, and effect modifiers should be provided. If these cannot be reported, an explanation should be provided.	Supplementary text S4 & Github repository.

Data sources/ measurement	8	For each variable of interest, give sources of data and details of methods of assessment (measurement). Describe comparability of assessment methods if there is more than one group	Method, supplementary tables S1-3, supplementary text S3 & code lists on GitHub.		.

Bias	9	Describe any efforts to address potential sources of bias	Sensitivity analyses to explore the effects of individuals having multiple events over an 8-week clock change period, described in Statistical analysis of Method section.		
Study size	10	Explain how the study size was arrived at	Method: Study Population section		
Quantitative variables	11	Explain how quantitative variables were handled in the analyses. If applicable, describe which groupings were chosen, and why	Method: outcomes, covariates, statistical analysis & supplementary text S2.		

Statistical methods	12	(a) Describe all statistical methods, including those used to control for confounding (b) Describe any methods used to examine subgroups and interactions<br>(c) Explain how missing data were addressed<br>(d) *Cohort study* - If applicable, explain how loss to follow-up was addressed<br>*Case-control study* - If applicable, explain how matching of cases and controls was addressed<br>*Cross-sectional study* - If applicable, describe analytical methods taking account of sampling strategy<br>(e) Describe any sensitivity analyses	Method, Statistical Analysis section		
Data access and cleaning methods		..		RECORD 12.1: Authors should describe the extent to which the investigators had access to the database population used to create the study population.	Method, study population section.

				RECORD 12.2: Authors should provide information on the data cleaning methods used in the study.	Method - Study population, outcomes & covariates sections. Supplementary table S2.
Linkage		..		RECORD 12.3: State whether the study included person-level, institutional-level, or other data linkage across two or more databases. The methods of linkage and methods of linkage quality evaluation should be provided.	Method: Study population section.
**Results**					
Participants	13	(a) Report the numbers of individuals at each stage of the study (*e.g.*, numbers potentially eligible, examined for eligibility, confirmed eligible, included in the study, completing follow-up, and analysed) (b) Give reasons for nonparticipation at each stage. (c) Consider use of a flow diagram		RECORD 13.1: Describe in detail the selection of the persons included in the study (*i.e.,* study population selection) including filtering based on data quality, data availability and linkage. The selection of included persons can be described in the text and/or by means of the study flow diagram.	Method, Study population & Figure 1.

Descriptive data	14	(a) Give characteristics of study participants (*e.g.*, demographic, clinical, social) and information on exposures and potential confounders<br>(b) Indicate the number of participants with missing data for each variable of interest (c) *Cohort study* - summarise follow-up time (*e.g.*, average and total amount)	Table 1		
Outcome data	15	*Cohort study* - Report numbers of outcome events or summary measures over time<br>*Case-control study* - Report numbers in each exposure category, or summary measures of exposure<br>*Cross-sectional study* - Report numbers of outcome events or summary measures	Figure 1.		

Main results	16	(a) Give unadjusted estimates and, if applicable, confounderadjusted estimates and their precision (e.g., 95% confidence interval). Make clear which confounders were adjusted for and why they were included (b) Report category boundaries when continuous variables were categorized<br>(c) If relevant, consider translating estimates of relative risk into absolute risk for a meaningful time period	Results, table 2 & supplementary tables S4-S7.		
Other analyses	17	Report other analyses done—<br>e.g., analyses of subgroups and interactions, and sensitivity analyses	Results: Events in the first week after the clock changes section & negative exposure section. Supplementary tables S4-S8.		
**Discussion**					
Key results	18	Summarise key results with reference to study objectives	Discussion, paragraph 1.		

Limitations	19	Discuss limitations of the study, taking into account sources of potential bias or imprecision. Discuss both direction and magnitude of any potential bias		RECORD 19.1: Discuss the implications of using data that were not created or collected to answer the specific research question(s). Include discussion of misclassification bias, unmeasured confounding, missing data, and changing eligibility over time, as they pertain to the study being reported.	Discussion (paragraphs 7-8)
Interpretation	20	Give a cautious overall interpretation of results considering objectives, limitations, multiplicity of analyses, results from similar studies, and other relevant evidence	Discussion, paragraphs 10-13		
Generalisability	21	Discuss the generalisability (external validity) of the study results	N/A		
**Other Information**					
Funding	22	Give the source of funding and the role of the funders for the present study and, if applicable, for the original study on which the present article is based	Funding statement		

Accessibility of protocol, raw data, and programming code		..		RECORD 22.1: Authors should provide information on how to access any supplemental information such as the study protocol, raw data, or programming code.	Supplementary text (including protocol), tables & figures provided & referenced in the paper. Data sharing statement provided including link to GitHub repository with full code.

*Reference: Benchimol EI, Smeeth L, Guttmann A, Harron K, Moher D, Petersen I, Sørensen HT, von Elm E, Langan SM, the RECORD Working Committee. The REporting of studies Conducted using Observational Routinely-collected health Data (RECORD) Statement. *PLoS Medicine* 2015; in press.

*Checklist is protected under Creative Commons Attribution (CC BY) license.

Table S1: List of dates of DST clock changes 2008-2019

Year	Date of spring clock change	Date of autumn clock change
2008	Sunday 30th March	Sunday 26th October
2009	Sunday 29th March	Sunday 25th October
2010	Sunday 28th March	Sunday 31st October
2011	Sunday 27th March	Sunday 30th October
2012	Sunday 25th March	Sunday 28th October
2013	Sunday 31st March	Sunday 27th October
2014	Sunday 30th March	Sunday 26th October
2015	Sunday 29th March	Sunday 25th October
2016	Sunday 27th March	Sunday 30th October
2017	Sunday 26th March	Sunday 29th October
2018	Sunday 25th March	Sunday 28th October
2019	Sunday 31st March	Sunday 27th October

Table S2: Derivation of covariates

Covariate	Categories	Derivation
Age	Anxiety: 10-50, >50<br>CVD: 40-75, >75<br>Depression: 10-45, >45<br>Eating disorders: 10-25, >25<br>Psychiatric conditions in A&E: 10-35, >35<br>Road traffic injuries: 0-30, >30<br>Self-harm: 10-30, >30<br>Sleep: 10-55, >55	Recorded in primary care record.<br><br>To maximise statistical power we created binary variables for age at the time of the clock change, with roughly equally-sized groups based on the median. The age categories were therefore different for each health condition.<br><br>CPRD does not collect exact date of birth due to the date needing to be anonymised. All patients without a day of birth had this inputted as 1. All participants without a month of birth (those aged 16 & over) had month inputted as July.
Sex	Male, Female, missing	Recorded in primary care record. 'Data not entered', 'indeterminate' or 'unknown' were categorised as missing. However, the missing category was not included in stratified results tables due to sample sizes being too small.
Marital status	In relationship, not in relationship, missing	Recorded in primary care record.<br><br>'Married', 'engaged', 'co-habiting', 're-married', 'stable relationship', 'civil partnership' recategorised as 'in relationship'.<br>'Single', 'widowed', 'divorced', 'separated' categorised as 'not in relationship'.<br>'Data not entered', 'unknown' categorised as missing.
Incident vs prevalent case	Yes, no	First ever event for this condition recorded in either primary or secondary care vs previous event for this condition recorded in either primary or secondary care.
Deprivation	Most deprived, rest, missing	Patient-level Index of Multiple Deprivation for England (2019) based on patient postcode. Provided by CPRD through third-party linkage to Office

		for National Statistics data.<br><br>20 categories recategorised to create a binary variable with the most deprived 20% included in the ‘most deprived’ category.<br><br>The missing category was not included in stratified results tables due to sample sizes being too small.
BMI	Overweight/obese, normal/underweight, missing	Recorded in primary care record. Defined as the most recent record prior to the clock change.<br>Extreme values (BMI <12 or >50 kg/m^2) were excluded. Remaining values recategorised: Overweight/obese: ≥ 25 kg/m^2, normal/underweight <25 kg/m^2.<br><br>https://www.nhs.uk/conditions/obesity/
Alcohol status	Current drinker, non/ex-drinker, missing	Recorded in primary care record. Defined as the most recent record prior to the clock change.<br><br>‘Non-drinker’ and ‘ex-drinker’ combined to create ‘non/ex-smoker’ category.
Smoking status	Current smoker, non/ex-smoker, missing	Recorded in primary care record. Defined as the most recent record prior to the clock change.<br><br>‘Non-smoker’ and ‘ex-smoker’ combined to create ‘non/ex-smoker’ category.
Systolic blood pressure	High, normal, missing	Recorded in primary care record. Defined as the most recent record prior to the clock change.<br><br>Raw values recategorised to be ‘High’: ≥140 mmHg or ‘normal’ <140 mmHg.<br><br>https://www.ncbi.nlm.nih.gov/pmc/articles/PMC381142/
Diastolic blood pressure	High, normal, missing	Recorded in primary care record. Defined as the most recent record prior to the clock change.<br><br>Raw values recategorised to be ‘High’: ≥90 mmHg or ‘normal’ <90 mmHg.<br><br>https://www.ncbi.nlm.nih.gov/pmc/articles/PMC381142/
Cardiovascular event subgroup	Atrial fibrillation/arrhythmia, myocardial infarction, stroke/transient ischemic attack, other	Based on Medical code or ICD10 code (primary care and HES APC data only)
Easter weekend	Yes, no	Binary variable based on whether the event date fell on one of the 5 days of the Easter weekend (including Maundy Thursday).
Region	9 numeric categories indicating where in England the GP practice is, based on its Strategic Health Authority	Provided by CPRD. Categories: North East, North West, Yorkshire & The Humber, East Midlands, West Midlands, East of England, London, South East and South West

Table S3: Number of GP practices per region

English region	Number of GP practices
North East	9
North West	68
Yorkshire & the Humber	14
East Midlands	12
West Midlands	44
East of England	37
London	63
South East	93
South West	51

Table S4: List of dates of 5-day Easter weekends 2008-2019

Year	Easter Sunday	Easter weekend start/end
2008	23rd March	20th-24th March
2009	12th April	9th-13th April
2010	4th April	1st-5th April
2011	24th April	21st-25th April
2012	8th April	5th-9th April
2013	31st March	28th March-1st April
2014	20th April	17th-21st April
2015	5th April	2nd-6th April
2016	27th March	24th-28th April
2017	16th April	13th-17th April
2018	1st April	29th March-2nd April
2019	21st April	18th-22nd April

Table S5: Adjusted* rate ratios of the number of events in the 1 week (including by day and Monday-Friday), 2 week and 4 week periods after the Spring and Autumn clock changes (England, 2008-2019) versus control periods**

**Anxiety**						
	**Spring**			**Autumn**		
	**Events**	**IRR (95% CI)**	**p value**	**Events**	**IRR (95% CI)**	**p value**
Sunday	3,869	0.94 (0.802, 1.092)	0.3997	4,468	0.98 (0.888, 1.072)	0.6114
Monday	18,008	1.00 (0.941, 1.065)	0.9780	20,600	0.94 (0.904, 0.986)	0.0095
Tuesday	18,324	1.00 (0.959, 1.034)	0.8269	18,956	0.96 (0.912, 1.018)	0.1832
Wednesday	17,062	1.00 (0.954, 1.044)	0.9303	18,229	0.96 (0.931, 0.999)	0.0460
Thursday	17,256	0.99 (0.933, 1.053)	0.7820	18,408	0.97 (0.942, 0.997)	0.0326
Friday	15,517	1.00 (0.950, 1.062)	0.8722	17,909	0.95 (0.915, 0.983)	0.0037
Saturday	4,892	0.97 (0.898, 1.038)	0.3395	5,434	1.06 (0.949, 1.189)	0.2917
Mon-Fri	86,167	1.00 (0.970, 1.041)	0.7904	94,102	0.96 (0.942, 0.974)	$7.133x10^{-07}$
1 week	94,928	0.99 (0.958, 1.029)	0.6954	104,004	0.97 (0.947, 0.984)	0.0002
2 weeks	94,928	0.98 (0.960, 0.995)	0.0111	104,004	1.01 (0.996, 1.019)	0.2073
4 weeks	94,928	1.00 (0.982, 1.013)	0.7492	104,004	1.01 (0.997, 1.027)	0.1135
**Acute cardiovascular disease**						
	**Spring**	**Autumn**				
	**Events**	**IRR (95% CI)**	**P value**	**Events**	**IRR (95% CI)**	**P value**
Sunday	27,671	1.00 (0.962, 1.034)	0.8902	27,275	0.97 (0.905, 1.032)	0.3130
Monday	48,591	1.02 (0.978, 1.055)	0.4312	50,109	0.95 (0.916, 0.983)	0.0039
Tuesday	49,904	1.03 (0.987, 1.071)	0.1774	49,640	1.00 (0.963, 1.036)	0.9494
Wednesday	49,020	1.04 (1.003, 1.077)	0.0324	48,232	0.97 (0.940, 1.003)	0.0770
Thursday	49.024	1.00 (0.953, 1.053)	0.9381	48,454	0.95 (0.914, 0.990)	0.0132
Friday	45,443	1.02 (0.979, 1.061)	0.3594	48,007	0.97 (0.936, 1.001)	0.0592
Saturday	29,150	1.01 (0.979, 1.044)	0.5044	29,792	1.05 (1.004, 1.107)	0.0328
Mon-Fri	241,982	1.02 (1.008, 1.038)	0.0026	244,442	0.97 (0.946, 0.990)	0.0046
1 week	298,803	1.02 (1.005, 1.030)	0.0058	301,509	0.98 (0.958, 0.999)	0.0423
2 weeks	298,803	1.00 (0.991, 1.015)	0.6558	301,509	1.00 (0.981, 1.015)	0.8351
4 weeks	298,803	0.99 (0.983, 1.001)	0.0747	301,509	1.01 (1.000, 1.0170	0.0505
**Depression**						
	**Spring**			**Autumn**		
	**Events**	**IRR (95% CI)**	**p value**	**Events**	**IRR (95% CI)**	**p value**
Sunday	7,118	0.92 (0.867, 0.984)	0.0143	7,807	0.95 (0.898, 1.006)	0.0780
Monday	50,837	0.99 (0.956, 1.016)	0.3344	55,831	0.93 (0.905, 0.963)	$1.593x10^{-5}$
Tuesday	51,149	0.98 (0.957, 0.998)	0.0326	51,149	0.96 (0.927, 1.001)	0.0561
Wednesday	48,860	1.00 (0.958, 1.037)	0.8621	48,615	0.97 (0.938, 0.995)	0.0203
Thursday	48,395	1.01 (0.965, 1.048)	0.8018	47,986	0.96 (0.926, 0.985)	0.0039
Friday	42,959	1.01 (0.976, 1.044)	0.5791	47,688	0.96 (0.916, 1.007)	0.0927
Saturday	9,113	1.03 (0.980, 1.089)	0.2234	9,989	0.99 (0.920, 1.067)	0.7990
Mon-Fri	242,200	1.00 (0.983, 1.020)	0.8759	251,269	0.96 (0.941, 0.970)	$6.080x10^{-09}$
1 week	258,431	1.00 (0.978, 1.012)	0.5765	269,065	0.96 (0.946, 0.970)	$9.080x10^{-11}$
2 weeks	258,431	0.98 (0.971, 0.994)	0.0033	269,065	0.98 (0.966, 0.991)	0.0010
4 weeks	258,431	0.99 (0.979, 1.007)	0.3131	269,065	1.00 (0.983, 1.009)	0.5217
**Eating disorders**						
	**Spring**			**Autumn**		
	**Events**	**IRR (95% CI)**	**p value**	**Events**	**IRR (95% CI)**	**p value**
Sunday	134	0.87 (0.392, 1.913)	0.7221	153	0.82 (0.532,1.262)	0.3656
Monday	545	0.99 (0.726, 1.340)	0.9284	531	0.76 (0.566, 1.032)	0.0790
Tuesday	486	0.99 (0.724, 1.348)	0.9395	463	0.64 (0.496, 0.833)	0.0008
Wednesday	460	1.09 (0.895, 1.319)	0.4009	459	1.01 (0.722, 1.422)	0.9380
Thursday	525	1.15 (0.873, 1.510)	0.3230	488	1.49 (1.128, 1.960)	0.0048
Friday	413	0.76 (0.516, 1.123)	0.1695	477	1.10 (0.912, 1.331)	0.3145
Saturday	172	0.92 (0.558, 1.501)	0.7254	183	0.94 (0.522, 1.706)	0.8478
Mon-Fri	2,429	1.01 (0.933, 1.094)	0.8023	2,418	0.99 (0.846, 1.161)	0.9069
1 week	2,735	1.00 (0.906, 1.104)	0.9984	2,754	0.97 (0.878, 1.082)	0.6293
2 weeks	2,735	0.97 (0.912, 1.033)	0.3465	2,754	0.99 (0.933, 1.045)	0.6601
4 weeks	2,735	1.04 (0.939, 1.147)	0.4672	2,754	0.98 (0.937, 1.032)	0.4983
**Psychiatric conditions (in A&E only)**						
	**Spring**			**Autumn**		
	**Events**	**IRR (95% CI)**	**p value**	**Events**	**IRR (95% CI)**	**p value**
Sunday	2695	0.92 (0.803, 1.060)	0.2546	2,977	0.90 (0.843, 0.962)	0.0018
Monday	2893	1.11 (0.968, 1.269)	0.1352	3,203	0.90 (0.793, 1.012)	0.0776
Tuesday	2814	0.97 (0.881, 1.078)	0.6151	3,106	0.93 (0.837, 1.026)	0.1450
Wednesday	2842	0.90 (0.750, 1.077)	0.2469	2,967	0.98 (0.878, 1.083)	0.6379
Thursday	2725	0.97 (0.892, 1.054	0.4718	3,017	0.95 (0.884, 1.019)	0.1477
Friday	2695	1.00 (0.846, 1.181	0.9947	2,981	0.98 (0.862, 1.111)	0.7373
Saturday	2604	1.05 (0.930, 1.193)	0.4154	2,822	0.97 (0.880, 1.075)	0.5819

Mon-Fri	13,969	0.99 (0.942, 1.035)	0.6011	15,274	0.94 (0.891, 1.002)	0.0579
1 week	19,268	0.99 (0.932, 1.044)	0.6289	21,073	0.94 (0.902, 0.984)	0.0078
2 weeks	19,268	0.98 (0.944, 1.016)	0.2745	21,073	1.00 (0.977, 1.029)	0.8411
4 weeks	19,268	1.00 (0.973, 1.034)	0.8257	21,073	1.02 (0.976, 1.063)	0.4049
**Road traffic injuries**						
	**Spring**			**Autumn**		
	**Events**	**IRR (95% CI)**	**p value**	**Events**	**IRR (95% CI)**	**p value**
Sunday	3838	1.02 (0.904, 1.147)	0.7624	4,210	0.90 (0.791, 1.022)	0.1036
Monday	7261	0.88 (0.789, 0.992)	0.0355	8,065	0.95 (0.839, 1.076)	0.4208
Tuesday	6854	0.98 (0.899, 1.076)	0.7226	7,250	0.87 (0.760, 0.993)	0.0390
Wednesday	6589	0.97 (0.886, 1.067	0.5532	7,118	0.99 (0.901, 1.093)	0.8724
Thursday	6474	1.07 (0.966, 1.180)	0.1979	7,107	0.98 (0.945, 1.013	0.2244
Friday	6408	1.03 (0.924, 1.145)	0.6054	7,406	1.09 (0.985, 1.198	0.0989
Saturday	4271	0.99 (0.927, 1.054)	0.7172	4,708	1.04 (0.921, 1.166)	0.5528
Mon-Fri	33,586	0.99 (0.940, 1.043)	0.7069	36,946	0.97 (0.942, 1.007)	0.1239
1 week	41,695	0.99 (0.951, 1.035)	0.7140	45,864	0.97 (0.943, 1.003)	0.0797
2 weeks	41,695	0.99 (0.952, 1.032)	0.6739	45,864	1.01 (0.993, 1.035)	0.2053
4 weeks	41,695	0.99 (0.952, 1.031)	0.6412	45,864	1.01 (0.995, 1.024)	0.1918
**Self-harm**						
	**Spring**			**Autumn**		
	**Events**	**IRR (95% CI)**	**p value**	**Events**	**IRR (95% CI)**	**p value**
Sunday	2812	0.94 (0.812, 1.097)	0.4518	2,986	1.03 (0.959, 1.096)	0.4660
Monday	3137	1.11 (1.034, 1.199)	0.0044	3,205	0.93 (0.841, 1.020	0.1210
Tuesday	3012	0.96 (0.868, 1.063)	0.4403	2,938	1.00 (0.865, 1.163	0.9643
Wednesday	3022	1.07 (0.932, 1.221)	0.3486	2,934	0.94 (0.862, 1.015)	0.1084
Thursday	2969	1.01 (0.958, 1.056)	0.8150	2,813	0.98 (0.906, 1.064	0.6549
Friday	2869	1.07 (0.942, 1.210)	0.3036	2,854	0.97 (0.846, 1.116)	0.6832
Saturday	2679	0.98 (0.820, 1.165)	0.7975	2,684	0.99 (0.862, 1.137)	0.8887
Mon-Fri	15,009	1.04 (1.004, 1.088)	0.0332	14,744	0.96 (0.907, 1.023)	0.2186
1 week	20,500	1.02 (0.981, 1.065)	0.2872	20,414	0.98 (0.929, 1.023)	0.3022
2 weeks	20,500	1.01 (0.990, 1.038)	0.2719	20,414	1.00 (0.966, 1.026)	0.7788
4 weeks	20,500	0.99 (0.968, 1.007)	0.2111	20,414	0.97 (0.938, 1.005)	0.0887
**Sleep disorders**						
	**Spring**			**Autumn**		
	**Events**	**IRR (95% CI)**	**p value**	**Events**	**IRR (95% CI)**	**p value**
Sunday	123	0.91 (0.364, 2.289	0.8465	154	1.35 (0.915, 1.981)	0.1311
Monday	6891	0.94 (0.868, 1.016	0.1167	7,273	0.94 (0.849, 1.041)	0.2345
Tuesday	6702	1.01 (0.913, 1.118	0.8404	6,419	0.87 (0.781, 0.966)	0.0095
Wednesday	6277	0.93 (0.842, 1.018	0.1113	6,106	0.92 (0.828, 1.024)	0.1289
Thursday	6101	1.04 (0.962, 1.127)	0.3197	5,878	0.90 (0.831, 0.983)	0.0181
Friday	5468	1.07 (0.962, 1.187	0.2189	6,042	0.95 (0.885, 1.020)	0.1540
Saturday	282	0.98 (0.738, 1.290	0.8633	274	0.73 (0.500, 1.076)	0.1131
Mon-Fri	31,439	1.00 (0.967, 1.034)	0.9997	31,748	0.92 (0.869, 0.967)	0.0013
1 week	31,844	1.00 (0.965, 1.032)	0.9082	32,146	0.92 (0.870, 0.969)	0.0019
2 weeks	31,844	0.98 (0.954, 1.009)	0.1749	32,146	0.93 (0.895, 0.963)	0.0001
4 weeks	31,844	0.96 (0.918, 0.997)	0.0350	32,146	0.97 (0.938, 0.995)	0.0213

IRR = Incidence Rate Ratio resulting from negative binomial regression. CI = Confidence Interval.
Events = total number of events in 8-week period (4 weeks before & 4 weeks after the clock change).
Data split by year (12 years), region (9 regions) & day (56 days in 8 week period) which gave us 6,048 data points for the 1,2 and 4 week analyses, 4,320 data points for the Monday-Friday analyses and 864 data points for the individual day analyses.

*Adjustments:
1 week, 2 weeks, 4 weeks & Monday-Friday analyses adjusted for day of the week and region. In addition, Spring analyses were adjusted for the 5 days of the Easter weekend.
Individual day analyses were adjusted for region.

Spring individual day analyses also adjusted for the 5 days of the Easter weekend (except for the Tuesday & Wednesday after where the Easter weekend covariate was omitted from analyses because of collinearity).

Number of events adjusted for the Sunday of the clock change having 25 or 23 hours instead of 24 using the following formulas:
Spring: (events on Sunday of clock change / 23) x 24. Autumn: (events on Sunday of clock change / 25) x 24.

**Control periods defined as:
- 1 week after clock change analysis: 4 weeks before & weeks 2-4 after the clock change.
- 2 weeks after clock change analysis: 4 weeks before & weeks 3-4 after the clock change.
- 4 weeks after clock change analysis: 4 weeks before the clock change.
- Monday-Friday in the week after the clock change analysis: weekdays in the 4 weeks before the clock change & weekdays in weeks 2-4 after the clock change.

Table S6: Sensitivity analysis: Adjusted* rate ratios of the number of health events in the week after the Spring and Autumn clock changes (England, 2008-2019) versus control period, with events of patients with an event for the same health condition in the 8 weeks before the study period removed for that clock change and only the first event for that health condition in the 8 week period kept for the remaining patients**

	**Spring (clocks go forward – less sleep)**			**Autumn (clocks go back – more sleep)**		
	**Events**	**IRR (95% CI)**	**P value**	**Events**	**IRR (95% CI)**	**P value**
Anxiety	63,278	0.98 (0.939,1.020)	0.3145	69,691	0.96 (0.940,0.988)	0.0035
Acute CVD	155,442	1.01 (0.999,1.028)	0.0637	159,356	0.97 (0.953,0.986)	0.0003
Depression	168,097	0.98 (0.957,0.997)	0.0241	178,174	0.95 (0.934,0.968)	1.713×10^{-08}
Eating disorders	1,694	1.00 (0.943,1.058)	0.9664	1,747	0.98 (0.857,1.112)	0.7180
Psychiatric conditions in A&E	15,937	0.99 (0.930,1.055)	0.7654	17,487	0.94 (0.895,0.994)	0.0283
Road traffic injuries	35,565	0.99 (0.945,1.036)	0.6512	39,015	0.97 (0.944,1.005)	0.1027
Self-harm	15,465	1.03 (0.981,1.074)	0.2507	15,221	0.97 (0.904,1.042)	0.4119
Sleep disorders	25,042	0.99 (0.952,1.024)	0.4915	25,754	0.88 (0.827,0.939)	0.0001

IRR = Incidence Rate Ratio resulting from negative binomial regression. CI = Confidence Interval.
Events = total number of events in 8-week period (4 weeks before & 4 weeks after the clock change). Data split by year (12 years), region (9 regions) & day (56 days in 8 week period) which gave us 6,048 data points.

*Analyses adjusted for day of week and region. In addition, Spring analyses were adjusted for the 5 days of the Easter weekend.
**Control period: 4 weeks before & weeks 2-4 after the negative control date.

Number of events adjusted for the Sunday of the clock change having 25 or 23 hours instead of 24 using the following formulas: Spring: (events on Sunday of clock change / 23) x 24. Autumn: (events on Sunday of clock change / 25) x 24.

Table S7: Sensitivity analysis: Adjusted* rate ratios of the number of health events in the week after the Spring and Autumn clock changes (England, 2008-2019) versus control period, with events for people with 20 or more events for the same health condition in a single 8-week clock change period excluded from that period & one random event kept for the remaining patients**

	**Spring (clocks go forward – less sleep)**			**Autumn (clocks go back – more sleep)**		
	**Events**	**IRR (95% CI)**	**P value**	**Events**	**IRR (95% CI)**	**P value**
Anxiety	75,740	0.98 (0.942,1.014)	0.2165	82,563	0.95 (0.936,0.972)	7.204×10^{-07}
Acute CVD	188,734	1.00 (0.990,1.018)	0.5819	191,51 2	0.97 (0.951,0.982)	3.838×10^{-05}
Depression	209,184	0.99 (0.965,1.005)	0.1496	217,49 8	0.95 (0.938,0.963)	6.826×10^{-14}
Eating disorders	2,085	0.98 (0.889,1.076)	0.6458	2,146	0.99 (0.864,1.123)	0.8227
Psychiatric conditions in A&E	17,293	0.99 (0.936,1.040)	0.6165	18,867	0.94 (0.898,0.985)	0.0088
Road traffic injuries	36,440	0.99 (0.946,1.033)	0.5940	39,945	0.96 (0.931,0.989)	0.0069
Self-harm	17,170	1.01 (0.966,1.066)	0.5621	17,054	0.96 (0.903,1.021)	0.1921
Sleep disorders	28,728	0.99 (0.951,1.023)	0.4747	28,917	0.91 (0.855,0.962)	0.0011

IRR = Incidence Rate Ratio resulting from negative binomial regression. CI = Confidence Interval.
Events = total number of events in 8-week period (4 weeks before & 4 weeks after the clock change).
Data split by year (12 years), region (9 regions) & day (56 days in 8 week period) which gave us 6,048 data points.

*Analyses adjusted for day of week and region. In addition, Spring analyses were adjusted for the 5 days of the Easter weekend.
**Control period: 4 weeks before & weeks 2-4 after the clock change.

Number of events adjusted for the Sunday of the clock change having 25 or 23 hours instead of 24 using the following formulas: Spring: (events on Sunday of clock change / 23) x 24. Autumn: (events on Sunday of clock change / 25) x 24.

Table S8: **Secondary analysis: Adjusted rate ratios of the number of health events in the week after the Spring and Autumn clock changes compared to the control period (England, 2008-2019), stratified by sociodemographics**

**ANXIETY**								
	**Spring**				**Autumn**			
	**Events**	**IRR (95% CI)**	**P value**	**Cochran's Q p value**	**Events**	**IRR (95% CI)**	**P value**	**Cochran's Q p value**
**Sex**								
Male	30859	1.01 (0.995,1.035)	0.1501	0.218	33379	0.96 (0.945,0.972)	$4.604x10^{-09}$	0.548
Female	64065	0.98 (0.938,1.030)	0.4749		70632	0.97 (0.940,0.998)	0.0367	
**Age**								
10-50	50938	1.00 (0.958,1.034)	0.8079	0.997	55539	0.95 (0.917,0.974)	0.0002	0.011
>50	43985	1.00 (0.950,1.042)	0.8386		48474	0.99 (0.972,1.003)	0.1167	
**Deprivation**								
Most deprived	27058	0.99 (0.938,1.041)	0.6444	0.732	30244	0.96 (0.943,0.984)	0.0006	0.763
Rest	67745	1.00 (0.966,1.033)	0.9326		73663	0.97 (0.947,0.989)	0.0030	
**Alcohol Status**								
Current drinker	54801	0.99 (0.951,1.037)	0.7454	0.930	59695	0.98 (0.959,1.001)	0.0651	0.036
Non/ex-drinker	23390	0.99 (0.950,1.038)	0.7583		25437	0.96 (0.919,1.012)	0.1428	
Missing	16730	1.01 (0.936,1.088)	0.8186		18885	0.92 (0.884,0.961)	0.0001	
**Incident case**								
Yes	31102	0.99 (0.936,1.047)	0.7220	0.800	34625	0.97 (0.933,1.016)	0.2131	0.517
No	63822	1.00 (0.965,1.033)	0.9251		69390	0.96 (0.939,0.978)	$3.132x10^{-05}$	
**CARDIOVASCULAR DISEASE (ACUTE)**								
	**Spring**				**Autumn**			
	**Events**	**IRR (95% CI)**	**P value**	**Cochran's Q p value**	**Events**	**IRR (95% CI)**	**P value**	**Cochran's Q p value**
**Sex**								
Male	160675	1.02 (1.009,1.040)	0.0018	0.373	161329	0.97 (0.945,1.006)	0.1071	0.756
Female	138130	1.01 (0.988,1.036)	0.3450		140163	0.98 (0.957,1.006)	0.1288	
**Age**								
40-75	139136	1.03 (1.012,1.042)	0.0003	0.163	138733	0.96 (0.932,0.993)	0.0179	0.095
>75	159660	1.01 (0.986,1.031)	0.4792		162766	0.99 (0.972,1.017)	0.6176	
**Deprivation**								
Most deprived	67465	1.02 (1.006,1.044)	0.0078	0.403	67754	1.00 (0.974,1.027)	0.9802	0.122
Rest	231110	1.01 (0.997,1.032)	0.1165		233570	0.97 (0.949,0.996)	0.0236	
**BMI**								
Overweight/obese	180424	1.03 (1.020,1.044)	$2.499x10^{-}$	0.010	183539	0.99 (0.963,1.009)	0.2148	0.568

			07					
Normal/underweight	88709	1.00 (0.972,1.018)	0.6793		89096	0.97 (0.939,1.008)	0.1282	
Missing	29655	1.00 (0.956,1.040)	0.8914		28886	0.95 (0.901,1.012)	0.1194	
**Alcohol Status**								
Current drinker	188075	1.02 (1.005,1.040)	0.0119	0.382	190168	0.98 (0.948,1.005)	0.1017	0.742
Non/ex-drinker	78198	1.02 (1.003,1.036)	0.0175		79467	0.99 (0.959,1.016)	0.3931	
Missing	32514	0.98 (0.932,1.037)	0.5311		31887	0.97 (0.938,1.005)	0.0933	
**Smoking Status**								
Current smoker	39240	1.01 (0.991,1.039)	0.2299	0.867	40038	0.97 (0.935,0.999)	0.0408	0.745
Non/ex-smoker	247418	1.02 (1.002,1.033)	0.0252		249503	0.98 (0.957,1.007)	0.1603	
Missing	12125	1.00 (0.938,1.066)	0.9920		11987	0.97 (0.928,1.020)	0.2529	
**Systolic Blood Pressure**								
High	101233	1.03 (1.003,1.058)	0.0277	0.314	101206	0.98 (0.950,1.008)	0.1441	0.450
Normal	185045	1.01 (0.998,1.021)	0.1107		188033	0.98 (0.964,1.001)	0.0600	
Missing	12510	1.03 (0.974,1.093)	0.2853		12280	0.94 (0.888,1.003)	0.0608	
**Diastolic Blood Pressure**								
High	26606	1.01 (0.972,1.056)	0.5493	0.874	27596	0.96 (0.924,1.006)	0.0957	0.391
Normal	259669	1.02 (1.003,1.031)	0.0174		261647	0.98 (0.962,1.003)	0.0873	
Missing	12512	1.03 (0.974,1.093)	0.2884		12281	0.94 (0.888,1.003)	0.0603	
**Incident case**								
Yes	58511	1.02 (0.999,1.048)	0.0648	0.814	58905	0.99 (0.964,1.016)	0.4531	0.472
No	240279	1.02 (1.006,1.033)	0.0055		242617	0.98 (0.956,0.999)	0.0450	
**CVD subgroup (GP & HES APC data only)**								
Atrial Fibrillation / Arrhythmia	107497	1.03 (1.009,1.042)	0.0021	0.654	107146	0.98 (0.958,1.008)	0.1872	0.698
Myocardial Infarction	11245	1.00 (0.950,1.050)	0.9510		11044	0.96 (0.916,1.016)	0.1729	
Stroke / TIA	35788	1.03 (0.983,1.085)	0.1975		35014	1.00 (0.947,1.058)	0.9708	
Other	80910	1.01 (0.993,1.036)	0.1881		81798	0.97 (0.947,0.995)	0.0184	
**DEPRESSION**								
	**Spring**				**Autumn**			
	**Events**	**IRR (95% CI)**	**P value**	**Cochran's Q p value**	**Events**	**IRR (95% CI)**	**P value**	**Cochran's Q p value**
**Sex**								
Male	84687	1.00 (0.985,1.022)	0.7021	0.266	88845	0.96 (0.947,0.975)	7.359×10^{-08}	0.711
Female	173723	0.99 (0.974,1.006)	0.2059		180221	0.96 (0.940,0.974)	1.202×10^{-06}	
**Age**								
10-45	127747	0.99 (0.970,1.013)	0.4439	0.771	131121	0.94 (0.915,0.958)	1.736×10^{-08}	0.015
>45	130670	1.00 (0.980,1.012)	0.5885		137958	0.98 (0.952,1.004)	0.0927	

**Deprivation**								
Most deprived	76732	0.98 (0.966,0.990)	0.0002	0.027	79666	0.96 (0.937,0.988)	0.0037	0.647
Rest	181392	1.00 (0.984,1.024)	0.7152		189162	0.96 (0.949,0.963)	$1.565x10^{-33}$	
**Alcohol Status**								
Current drinker	157444	0.99 (0.972,1.010)	0.3628	0.977	163854	0.96 (0.941,0.978)	$3.031x10^{-05}$	0.528
Non/ex-drinker	57519	0.99 (0.970,1.020)	0.6690		59415	0.96 (0.943,0.982)	0.0002	
Missing	43450	0.99 (0.962,1.025)	0.6636		45817	0.95 (0.923,0.969)	$5.939x10^{-06}$	
**Incident case**								
Yes	51392	0.98 (0.958,1.006)	0.1310	0.218	54133	0.98 (0.960,0.997)	0.0217	0.032
No	207037	1.00 (0.983,1.017)	0.9874		214940	0.95 (0.938,0.967)	$5.110x10^{-10}$	
**EATING DISORDERS**								
	**Spring**				**Autumn**			
	**Events**	**IRR (95% CI)**	**P value**	**Cochran's Q p value**	**Events**	**IRR (95% CI)**	**P value**	**Cochran's Q p value**
**Sex**								
Male	211	1.40 (0.927,2.108)	0.1100	0.164	208	1.05 (0.755,1.466)	0.7657	0.655
Female	2524	0.97 (0.846,1.108)	0.6422		2546	0.97 (0.883,1.062)	0.4915	
**Age**								
10-25	1295	1.02 (0.930,1.123)	0.6482	0.618	1316	0.99 (0.856,1.143)	0.8869	0.763
>25	1440	0.98 (0.861,1.118)	0.7761		1438	0.96 (0.856,1.080)	0.5087	
**Deprivation**								
Most deprived	561	1.04 (0.784,1.378)	0.7877	0.816	620	1.17 (0.840,1.642)	0.3481	0.220
Rest	2158	1.00 (0.920,1.093)	0.9498		2130	0.92 (0.841,1.003)	0.0572	
**BMI**								
Overweight/obese	319	1.02 (0.806,1.302)	0.8433	0.879	310	0.98 (0.656,1.458)	0.9139	0.988
Normal/underweight	1668	1.01 (0.898,1.137)	0.8647		1606	0.97 (0.884,1.062)	0.4967	
Missing	748	0.97 (0.868,1.092)	0.6461		838	0.98 (0.831,1.164)	0.8451	
**Incident case**								
Yes	845	0.92 (0.770,1.088)	0.3148	0.209	830	1.11 (0.869,1.418)	0.4016	0.198
No	1890	1.04 (0.936,1.152)	0.4770		1924	0.92 (0.825,1.024)	0.1247	
**PSYCHIATRIC CONDITIONS (IN A&E ONLY)**								
	**Spring**				**Autumn**			
	**Events**	**IRR (95% CI)**	**P value**	**Cochran's Q p value**	**Events**	**IRR (95% CI)**	**P value**	**Cochran's Q p value**
**Sex**								
Male	9289	0.98 (0.895,1.064)	0.5814	0.813	10170	1.00 (0.948,1.057)	0.9589	0.005
Female	9976	0.99 (0.934,1.045)	0.6755		10906	0.89 (0.835,0.947)	0.0003	

**Age**								
10-35	9430	0.97 (0.891,1.059)	0.5065	0.704	10099	0.90 (0.843,0.954)	0.0006	0.029
>35	9835	0.99 (0.924,1.066)	0.8359		10977	0.99 (0.930,1.044)	0.6226	
**Deprivation**								
Most deprived	7416	0.97 (0.877,1.066)	0.4958	0.718	8044	0.93 (0.876,0.989)	0.0199	0.673
Rest	11828	0.99 (0.921,1.060)	0.7438		13001	0.95 (0.880,1.027)	0.1990	
**Alcohol Status**								
Current drinker	8541	1.02 (0.929,1.126)	0.6493	0.389	9137	0.95 (0.902,0.992)	0.0225	0.002
Non/ex-drinker	4134	0.93 (0.857,1.019)	0.1236		4600	1.05 (0.987,1.119)	0.1193	
Missing	6590	0.96 (0.912,1.011)	0.1232		7339	0.87 (0.800,0.957)	0.0034	
**Incident case**								
Yes	11844	1.03 (0.988,1.076)	0.1649	0.019	13000	0.92 (0.858,0.984)	0.0151	0.129
No	7421	0.90 (0.810,1.006)	0.0631		8076	0.99 (0.927,1.052)	0.6967	
**ROAD TRAFFIC INJURIES**								
	**Spring**				**Autumn**			
	**Events**	**IRR (95% CI)**	**P value**	**Cochran's Q p value**	**Events**	**IRR (95% CI)**	**P value**	**Cochran's Q p value**
**Sex**								
Male	22798	0.97 (0.922,1.026)	0.3124	0.400	25097	0.98 (0.946,1.024)	0.4351	0.539
Female	18889	1.01 (0.944,1.080)	0.7819		20773	0.97 (0.926,1.009)	0.1218	
**Age**								
0-30	19024	1.02 (0.956,1.079)	0.6108	0.248	20586	0.99 (0.937,1.048)	0.7441	0.469
>30	22665	0.97 (0.918,1.022)	0.2395		25286	0.97 (0.928,1.005)	0.0884	
**Deprivation**								
Most deprived	12327	0.96 (0.892,1.028)	0.2336	0.279	13179	0.93 (0.857,1.015)	0.1054	0.163
Rest	29333	1.00 (0.960,1.046)	0.9218		32657	0.99 (0.961,1.027)	0.6897	
**Alcohol Status**								
Current drinker	19015	0.95 (0.911,1.000)	0.0498	0.038	21229	0.96 (0.935,0.994)	0.0172	0.002
Non/ex-drinker	6956	0.95 (0.892,1.011)	0.1070		7477	0.89 (0.851,0.938)	$6.466x10^{-06}$	
Missing	15717	1.05 (0.986,1.116)	0.1284		17166	1.03 (0.965,1.100)	0.3692	
**SELF-HARM**								
	**Spring**				**Autumn**			
	**Events**	**IRR (95% CI)**	**P value**	**Cochran's Q p value**	**Events**	**IRR (95% CI)**	**P value**	**Cochran's Q p value**
**Sex**								
Male	8438	1.04 (0.964,1.112)	0.3432	0.592	8551	1.02 (0.946,1.102)	0.5961	0.152
Female	12056	1.01 (0.968,1.057)	0.6091		11864	0.95 (0.882,1.015)	0.1211	

**Age**								
10-30	10311	1.03 (0.965,1.089)	0.4204	**0.838**	9962	0.95 (0.893,1.009)	0.0928	0.288
>30	10187	1.02 (0.979,1.058)	0.3759		10455	1.00 (0.927,1.083)	0.9595	
**Deprivation**								
Most deprived	7692	1.08 (1.023,1.141)	0.0054	0.036	7559	0.94 (0.865,1.026)	0.1734	0.324
Rest	12771	0.99 (0.931,1.052)	0.7409		12814	0.99 (0.934,1.056)	0.8240	
**Alcohol Status**								
Current drinker	8524	0.99 (0.938,1.049)	0.7807	0.308	8687	1.02 (0.973,1.079)	0.3648	0.117
Non/ex-drinker	3523	1.06 (0.989,1.146)	0.0978		3587	0.93 (0.827,1.036)	0.1809	
Missing	8451	1.03 (0.968,1.104)	0.3160		8143	0.95 (0.881,1.023)	0.1716	
**Incident case**								
Yes	9893	1.02 (0.961,1.083)	0.5083	0.937	9526	0.92 (0.890,0.960)	4.082×10^{-05}	0.018
No	10605	1.02 (0.965,1.086)	0.4334		10891	1.02 (0.951,1.099)	0.5486	
**SLEEP**								
	**Spring**				**Autumn**			
	**Events**	**IRR (95% CI)**	**P value**	**Cochran's Q p value**	**Events**	**IRR (95% CI)**	**P value**	**Cochran's Q p value**
**Sex**								
Male	12651	0.99 (0.957,1.021)	0.4871	0.563	12711	0.92 (0.856,0.992)	0.0297	0.925
Female	19192	1.01 (0.955,1.061)	0.8006		19434	0.93 (0.861,0.996)	0.0375	
**Age**								
10-55	16684	1.00 (0.971,1.024)	0.8367	0.857	16784	0.94 (0.891,0.982)	0.0066	0.415
>55	15160	1.00 (0.958,1.049)	0.9292		15362	0.91 (0.866,0.954)	0.0001	
**Deprivation**								
Most deprived	7593	1.00 (0.935,1.080)	0.9016	0.973	7646	0.95 (0.897,1.006)	0.0767	0.284
Rest	24222	1.00 (0.969,1.038)	0.8578		24480	0.91 (0.852,0.965)	0.0021	
**Alcohol Status**								
Current drinker	20779	1.01 (0.974,1.049)	0.5756	0.258	20876	0.90 (0.841,0.970)	0.0051	0.341
Non/ex-drinker	6972	0.96 (0.909,1.012)	0.1273		7162	0.97 (0.912,1.025)	0.2610	
Missing	4093	1.01 (0.928,1.109)	0.7541		4108	0.94 (0.895,0.993)	0.0255	
**Incident case**								
Yes	14670	1.03 (0.978,1.081)	0.2764	0.123	15204	0.90 (0.851,0.961)	0.0012	0.352
No	17174	0.97 (0.929,1.021)	0.2726		16942	0.94 (0.894,0.985)	0.0104	

IRR = Incidence Rate Ratio resulting from negative binomial regression. CI = Confidence Interval.
Events = total number of events in 8-week period (4 weeks before & 4 weeks after the clock change).
Data split by year (12 years), region (9 regions) & day (56 days in 8 week period) which gave us 6,048 data points.

Control period: 4 weeks before & weeks 2-4 after the negative control date.
Analyses adjusted for day of week and region. In addition, Spring analyses were adjusted for the 5 days of the Easter weekend.
Number of events adjusted for the Sunday of the clock change having 25 or 23 hours instead of 24 using the following formulas: Spring: (events on Sunday of clock change / 23) x 24. Autumn: (events on Sunday of clock change / 25) x 24.

Table S9: Negative control exposure analysis: Adjusted* rate ratios of the number of health events in the week after the Spring and Autumn negative controls (4 weeks before the clock changes) (England, 2008-2019) versus control period**

	**Spring negative control**			**Autumn negative control**		
	**Events**	**IRR (95% CI)**	**P value**	**Events**	**IRR (95% CI)**	**P value**
Anxiety	96,700	1.00 (0.980,1.013)	0.6749	103,150	1.01 (0.987,1.032)	0.4255
Acute CVD	305,054	1.00 (0.986,1.011)	0.8136	296,563	1.00 (0.982,1.008)	0.4507
Depression	268,746	1.01 (0.993,1.023)	0.3047	267,634	1.02 (1.006,1.043)	0.0106
Eating disorders	2,645	0.99 (0.885,1.112)	0.8862	2,686	1.02 (0.940,1.101)	0.6678
Psychiatric conditions in A&E	19,066	1.02 (0.969,1.075)	0.4438	20,846	1.00 (0.949,1.061)	0.9061
Road traffic injuries	43,293	1.01 (0.971,1.041)	0.7719	45,129	1.02 (0.977,1.061)	0.3974
Self-harm	20,548	1.02 (0.986,1.049)	0.2985	20,742	1.00 (0.954,1.054)	0.9120
Sleep disorders	35,322	1.04 (1.021,1.053)	$3.222x10^{-06}$	32,502	1.00 (0.962,1.039)	0.9835

IRR = Incidence Rate Ratio resulting from negative binomial regression. CI = Confidence Interval. Events = total number of events in 8-week period (4 weeks before & 4 weeks after the clock change). Data split by year (12 years), region (9 regions) & day (56 days in 8 week period) which gave us 6,048 data points.

*Analyses adjusted for day of week and region. In addition, Spring analyses were adjusted for the 5 days of the Easter weekend.

**Control period: 4 weeks before & weeks 2-4 after the negative control date.

Figure S1: Exposure and control periods for primary and secondary analyses.

S1a: Primary analysis: Exposed period = first week after the clock change

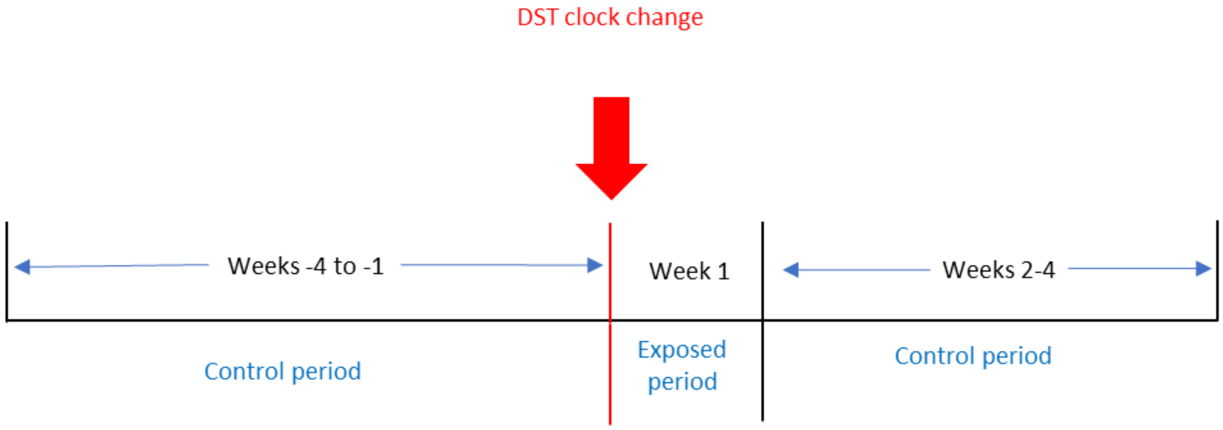

S1b: Secondary analysis. Exposed period = individual days of the week in the first week after the clock change (diagram shows Monday)

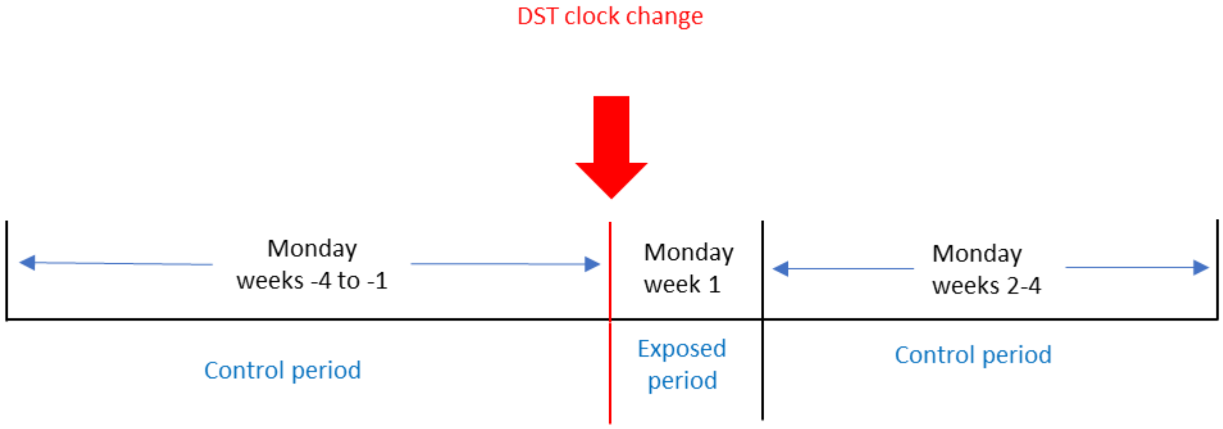

S1c: Secondary analysis: Exposed period = Monday to Friday in first week after the clock change

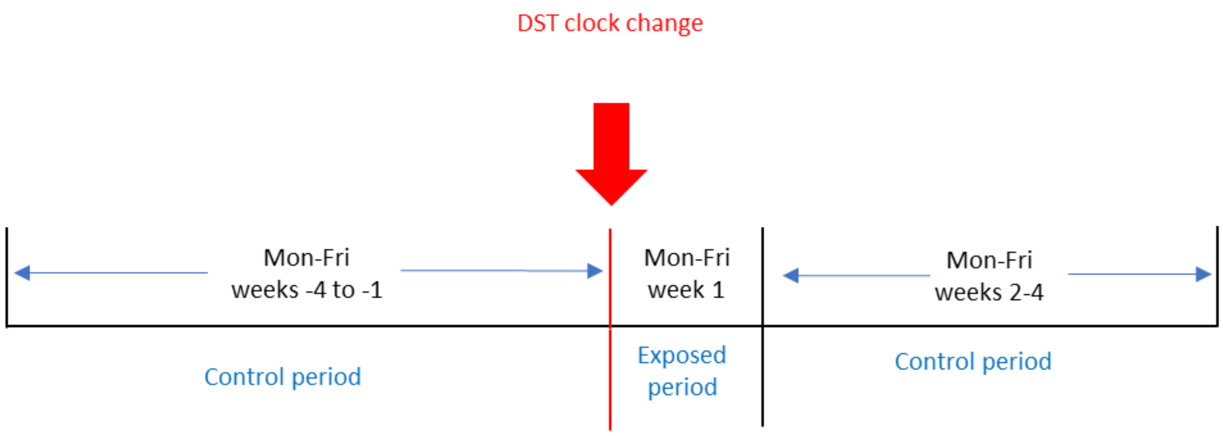

S1d: Secondary analysis: Exposed period = first 2 weeks after clock change

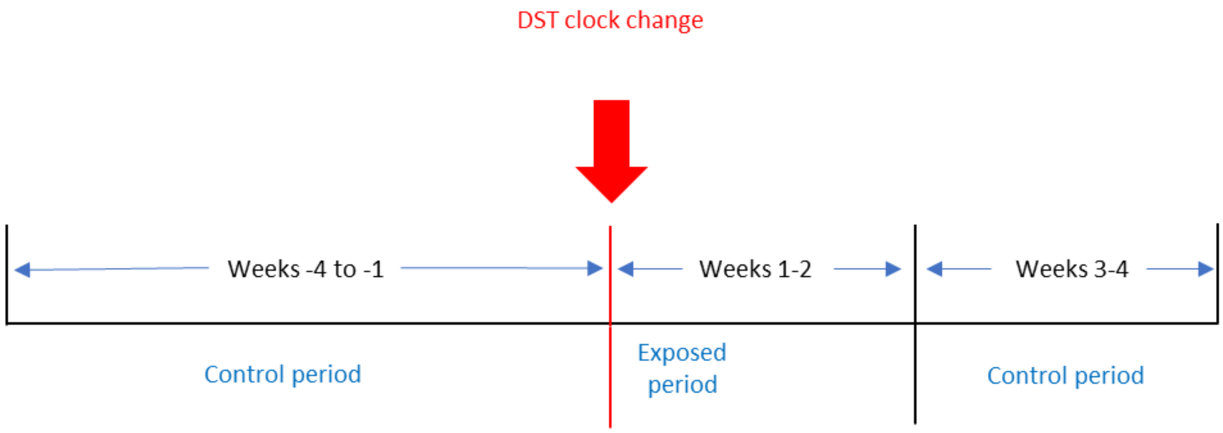

S1e: Secondary analysis: Exposed period = first 4 weeks after clock change

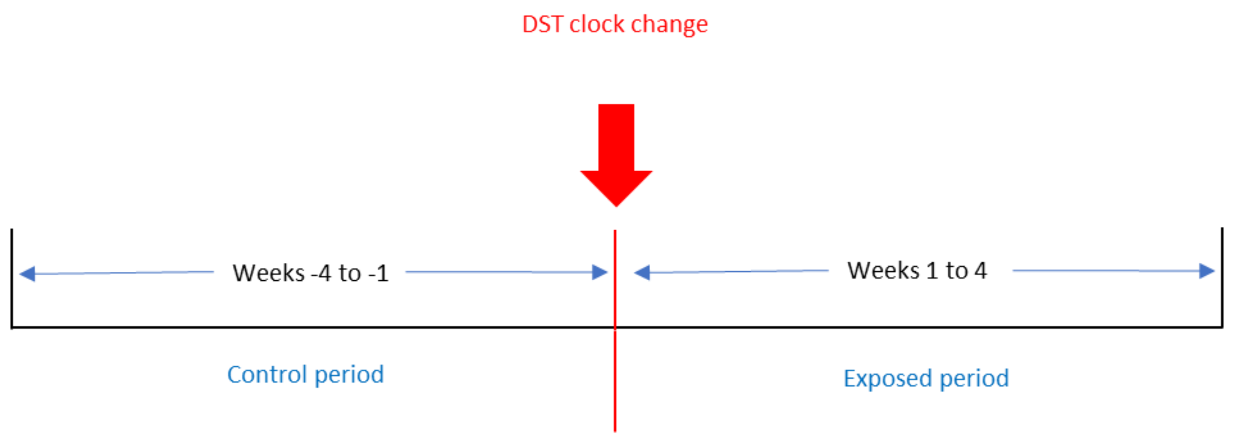

S1f: Negative control analysis: Exposed period = week 4 before clock change

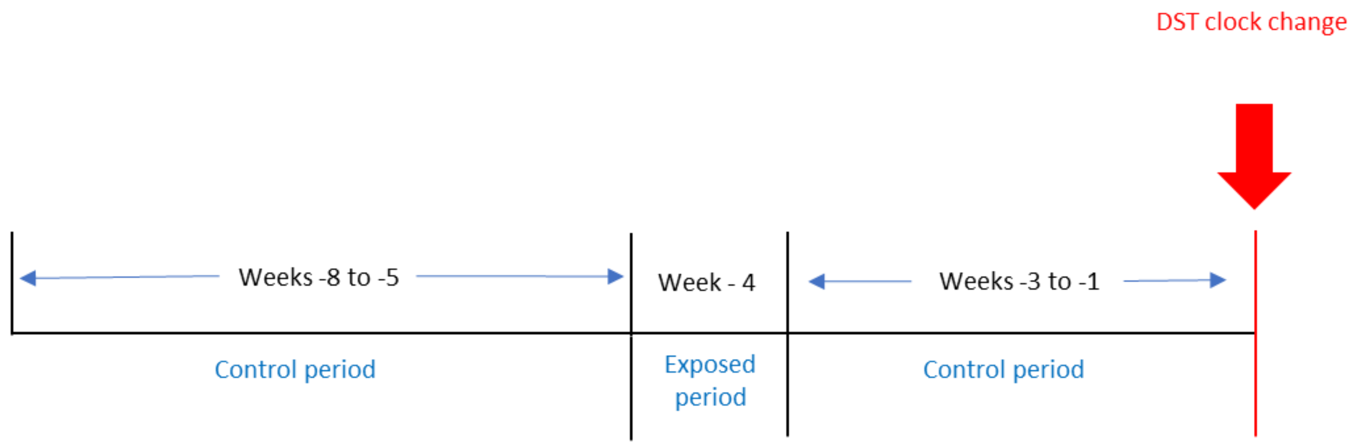

Figure S2: Anxiety events per day over the Spring clock changes.

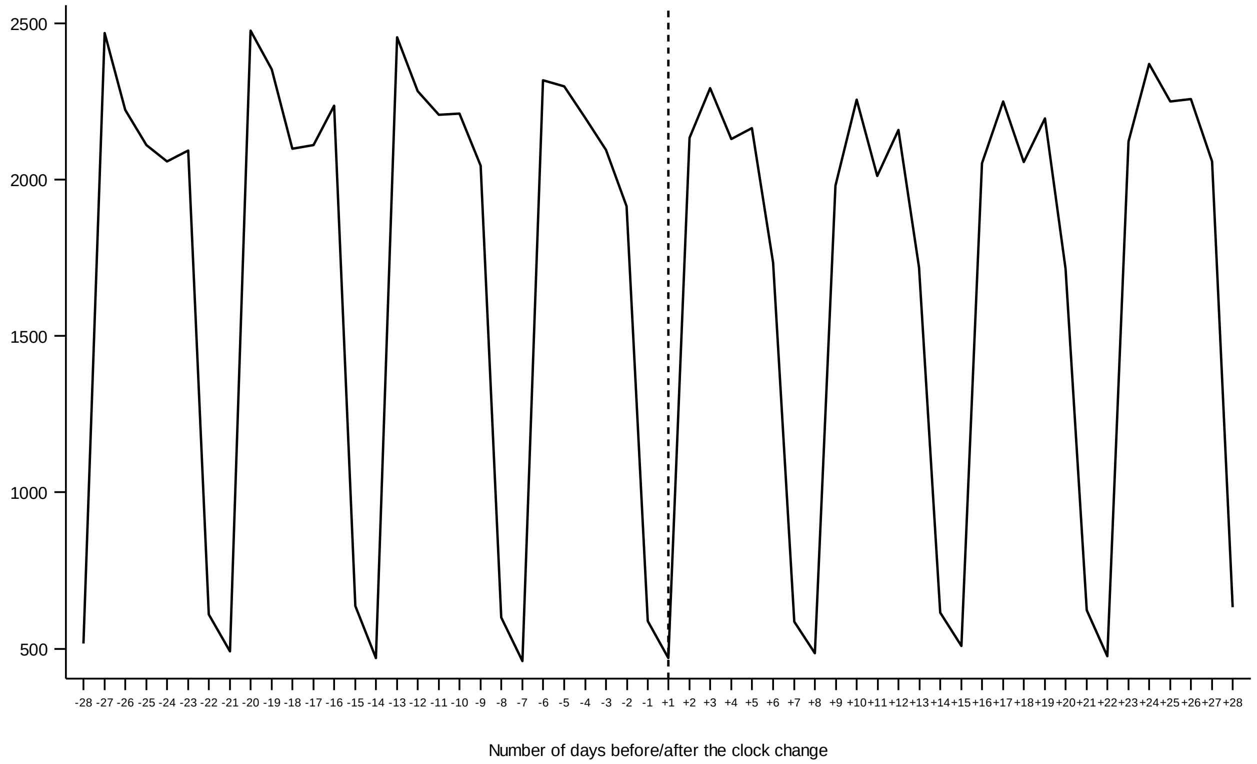

*Dashed line = day of the clock change.

Figure S3: CVD events per day over the Spring clock changes.

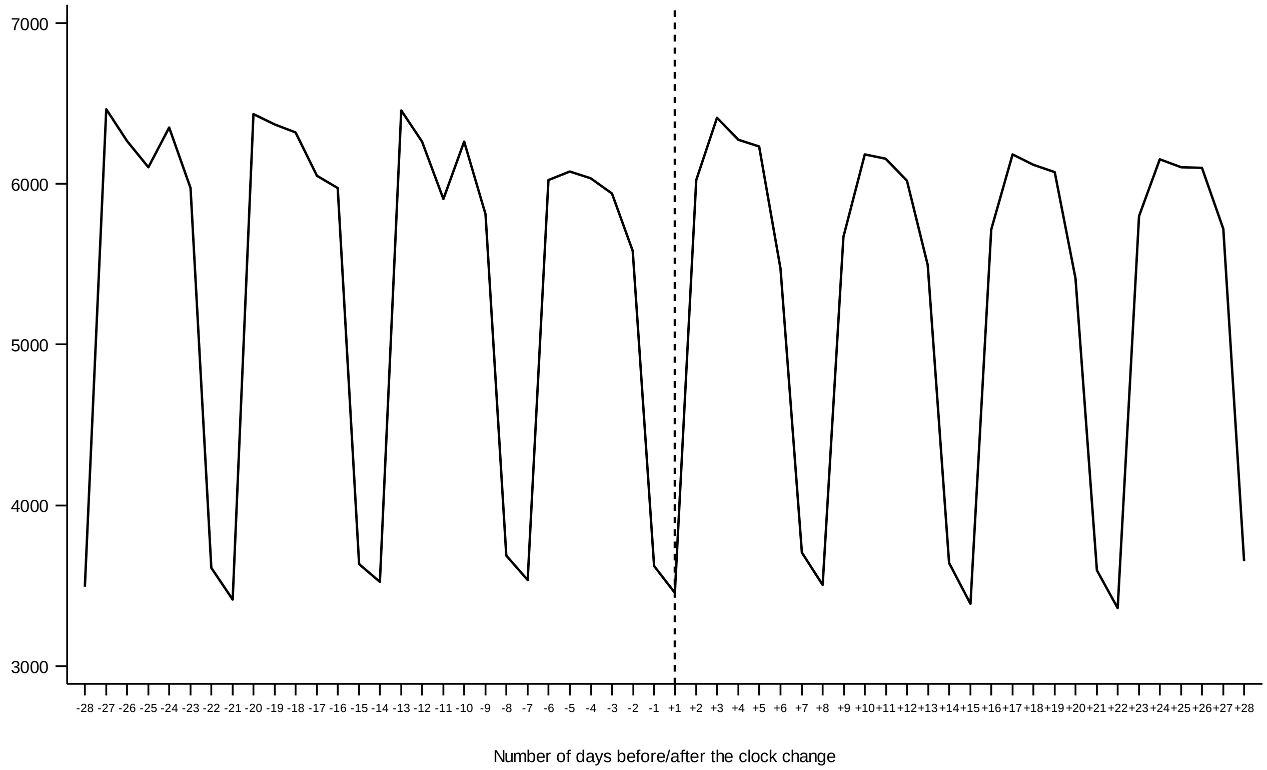

*Dashed line = day of the clock change.

Figure S4: Depression events per day over the Spring clock changes.

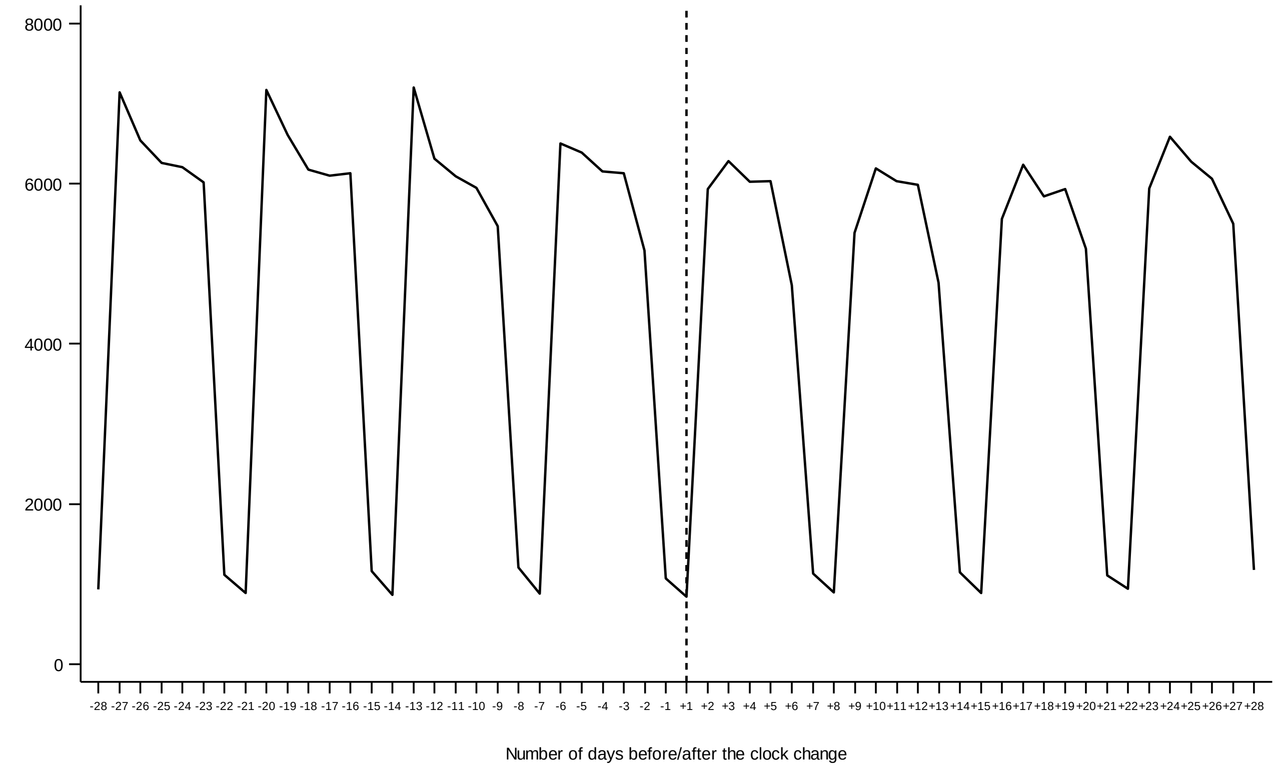

*Dashed line = day of the clock change.

Figure S5: Eating disorder events per day over the Spring clock changes.

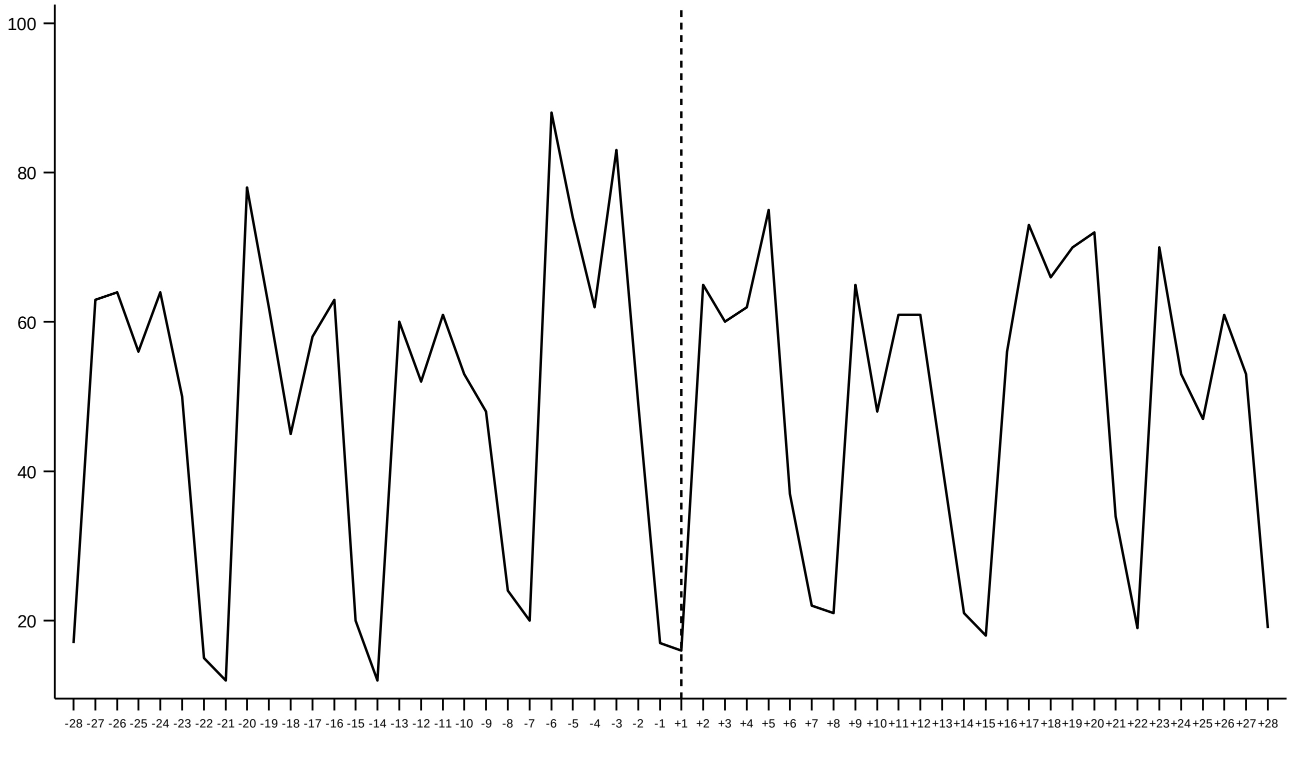

*Dashed line = day of the clock change.

Figure S6: Psychiatric conditions events per day over the Spring clock changes.

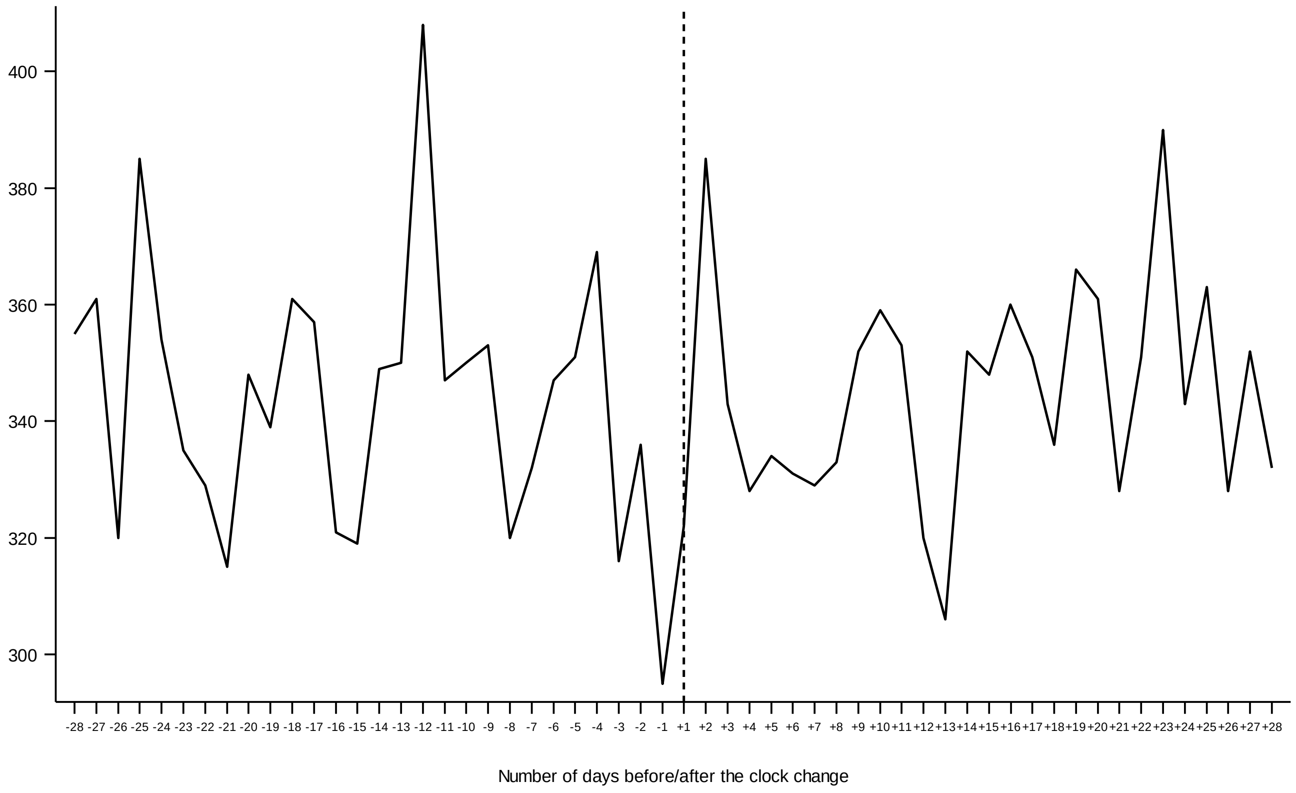

*Dashed line = day of the clock change.

Figure S7: Road traffic injury events per day over the Spring clock changes.

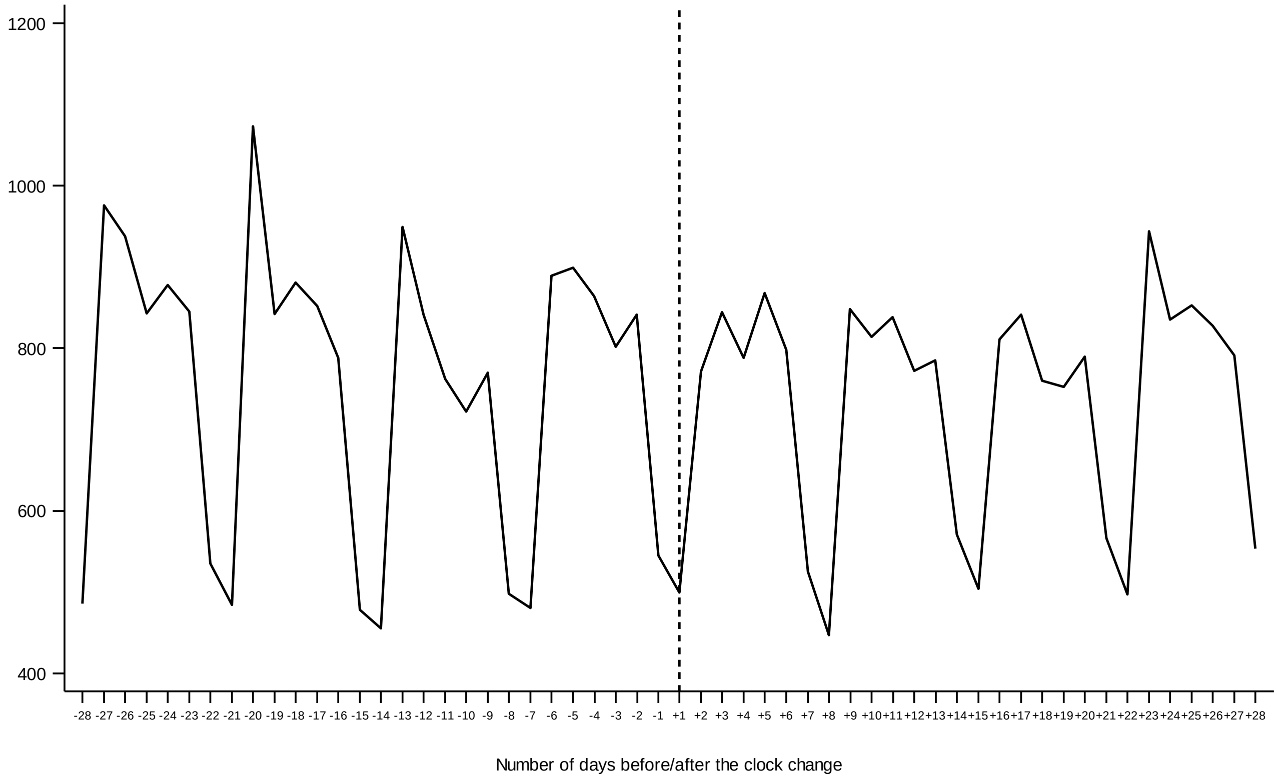

*Dashed line = day of the clock change.

Figure S8: Self-harm events per day over the Spring clock changes.

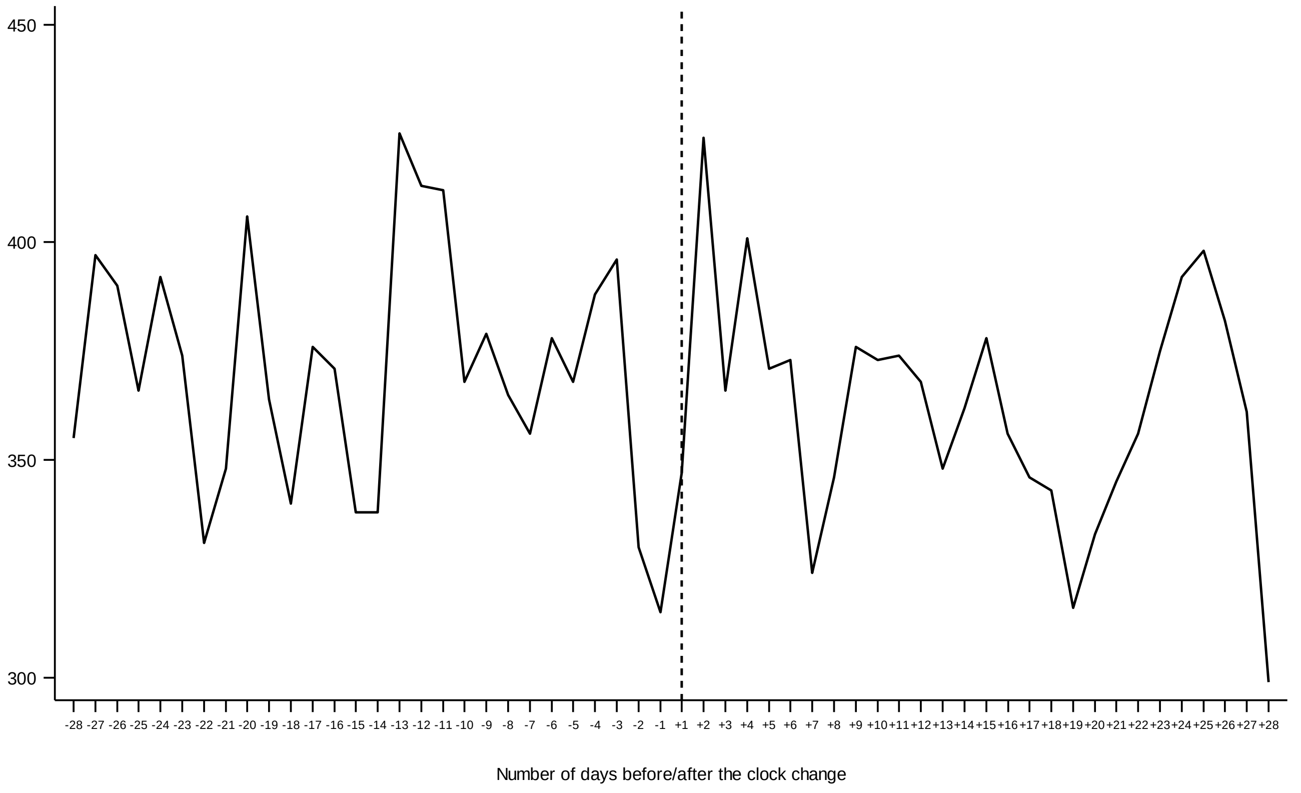

*Dashed line = day of the clock change.

Figure S9: Sleep disorder events per day over the Spring clock changes.

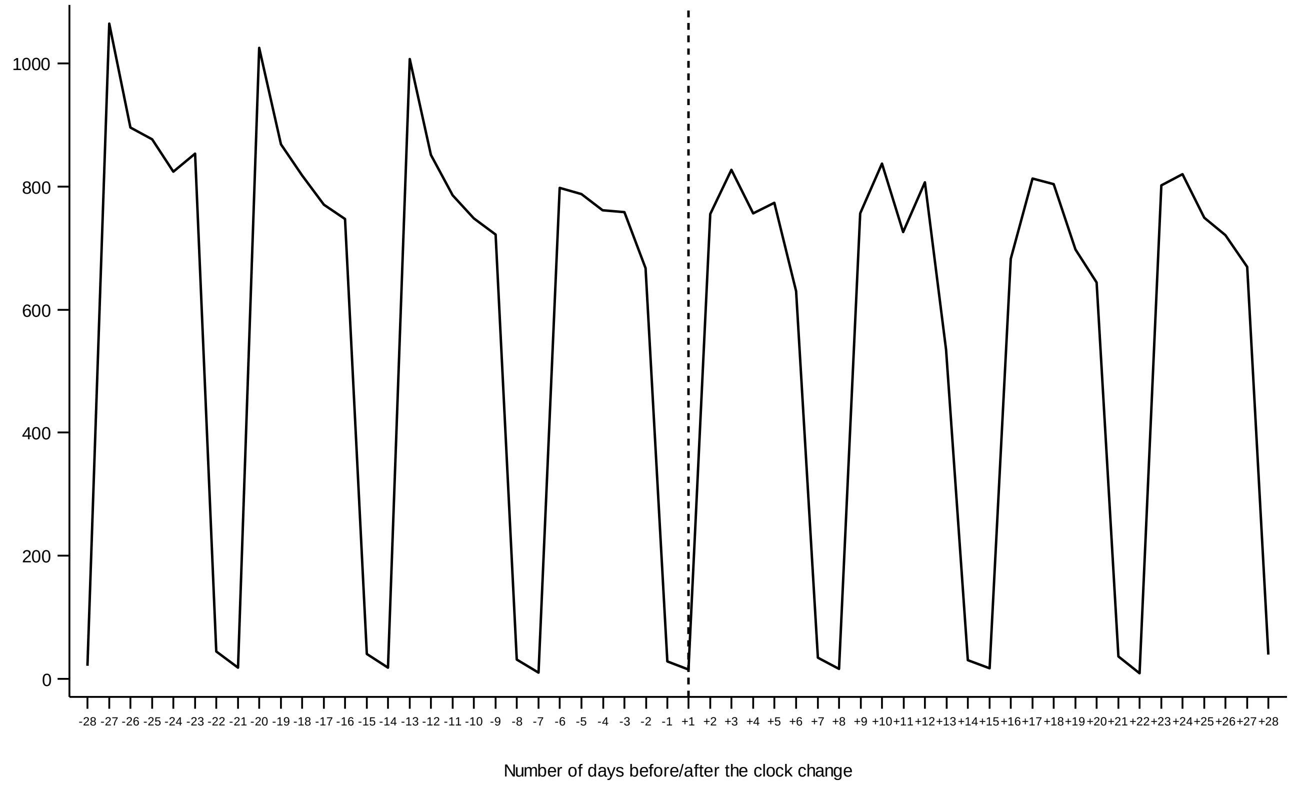

*Dashed line = day of the clock change.

Figure S10: Anxiety events per day over the Autumn clock changes.

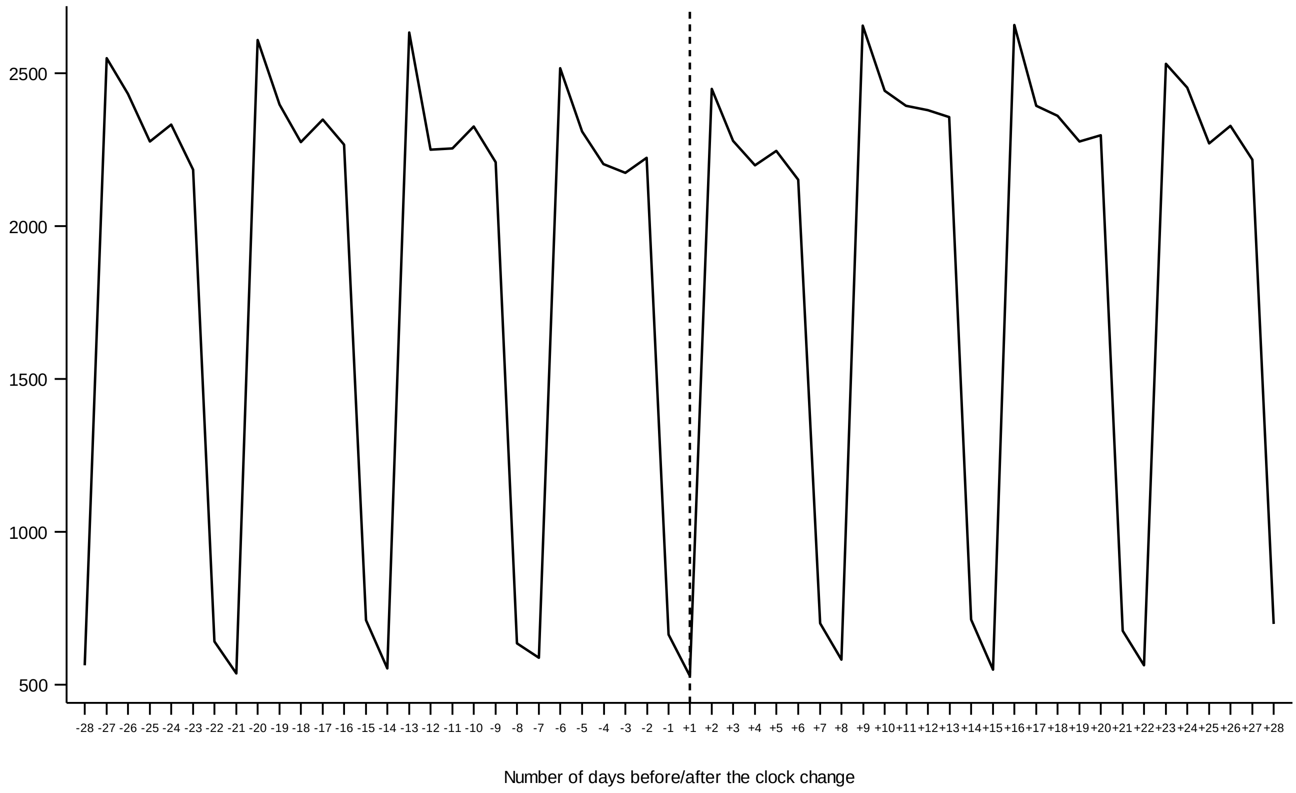

*Dashed line = day of the clock change.

Figure S11: CVD events per day over the Autumn clock changes.

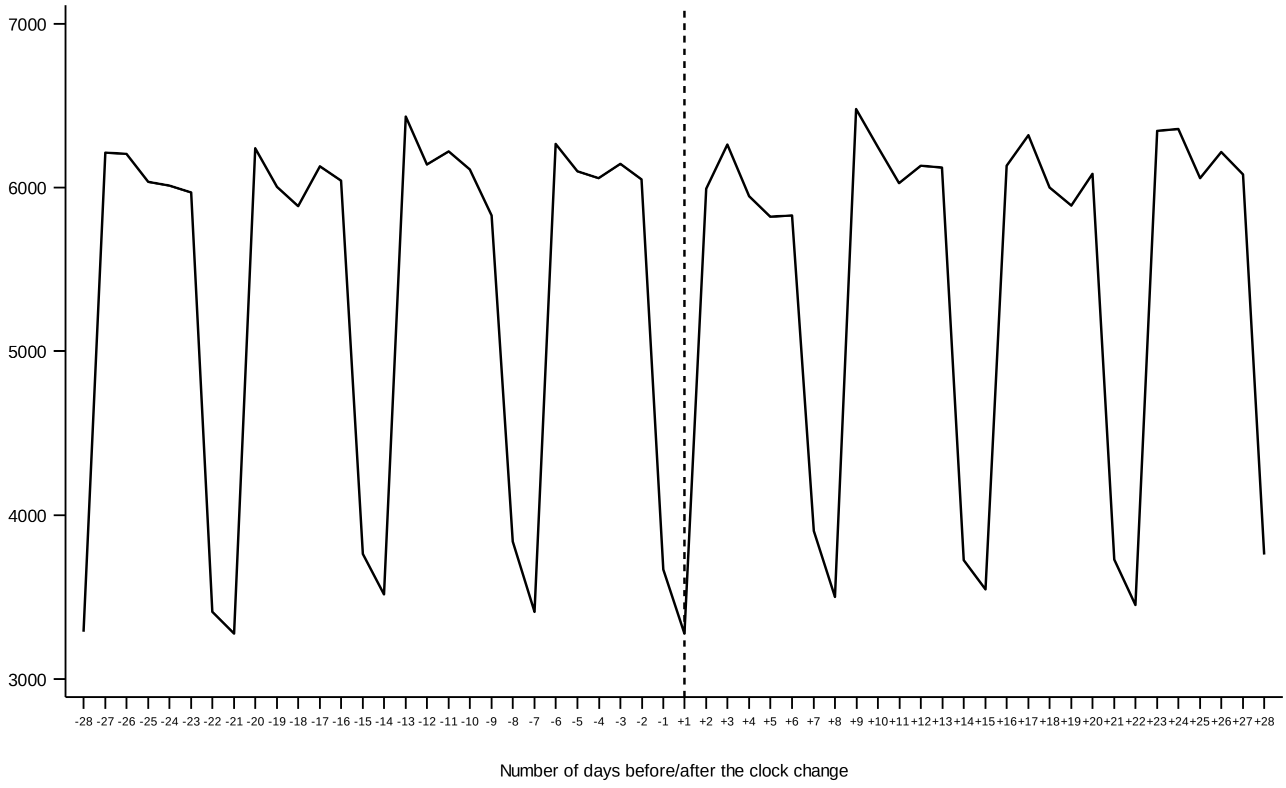

*Dashed line = day of the clock change.

Figure S12: Depression events per day over the Autumn clock changes.

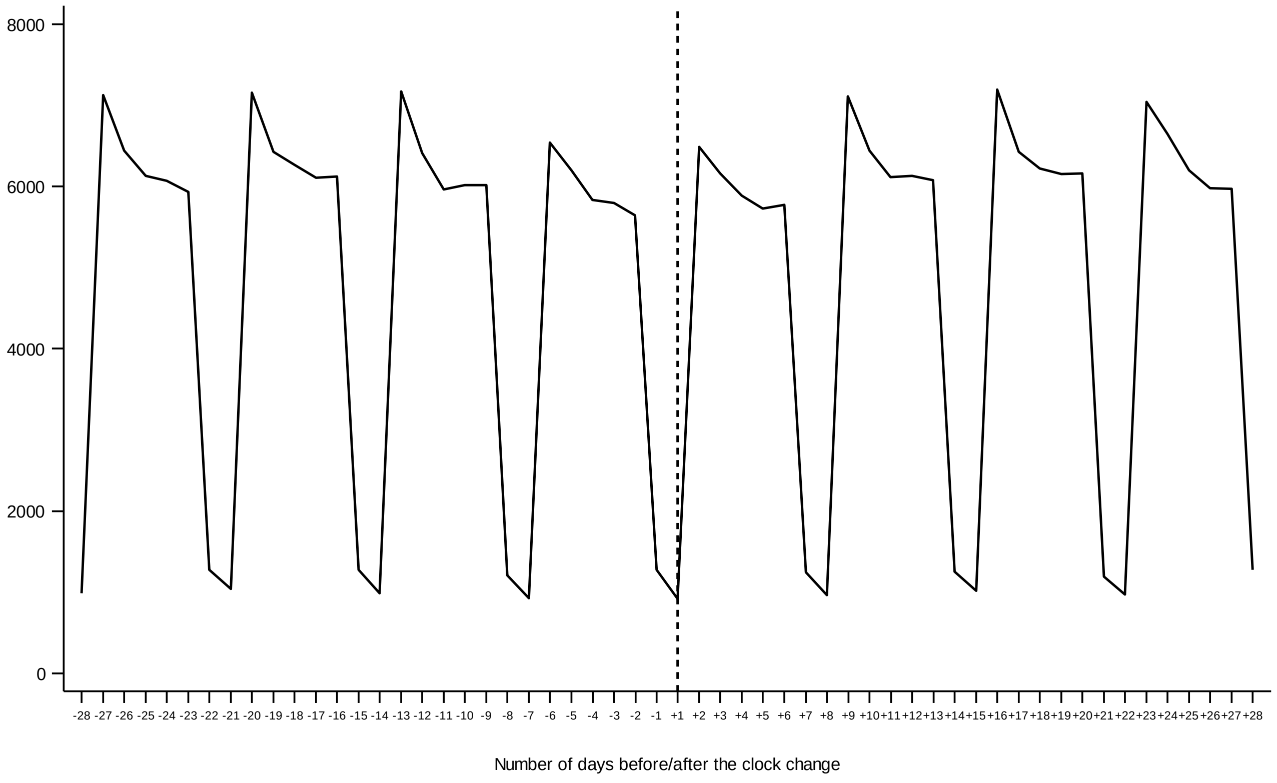

*Dashed line = day of the clock change.

Figure S13: Eating disorder events per day over the Autumn clock changes.

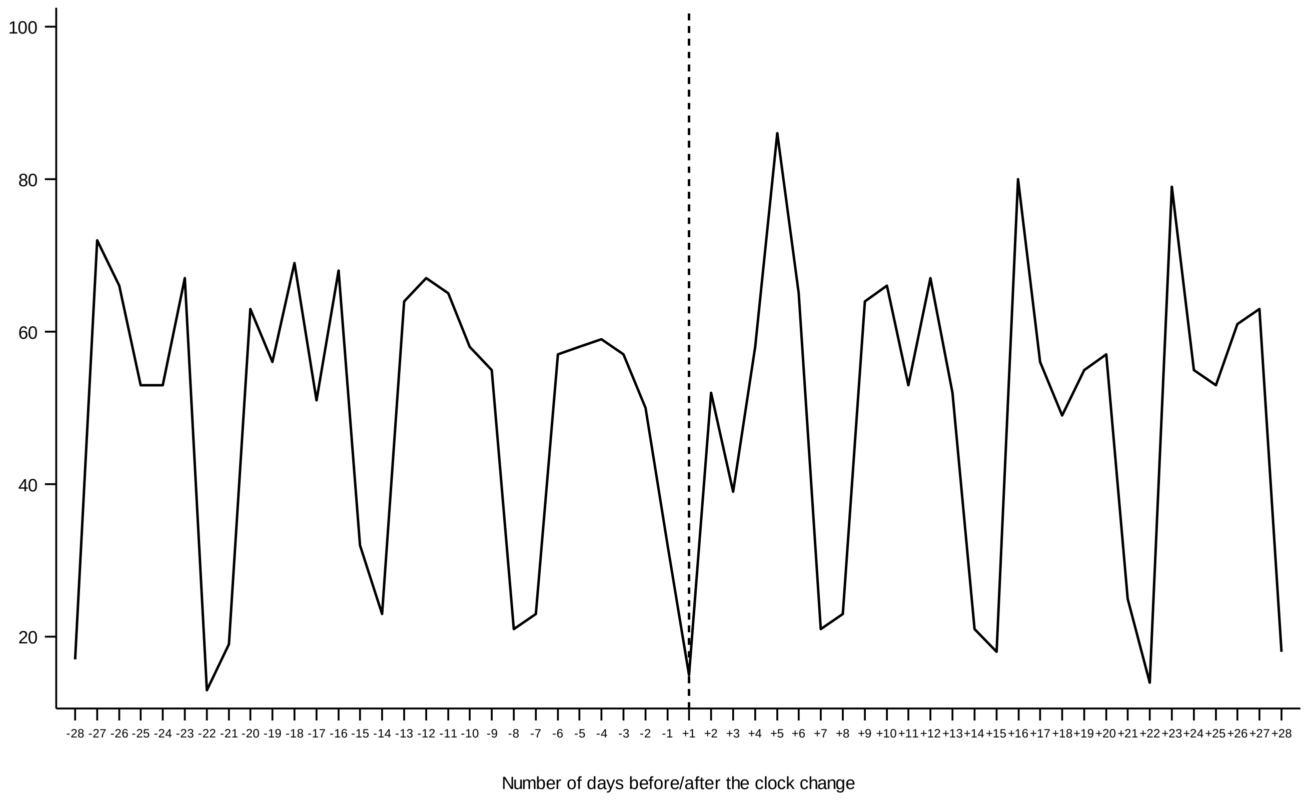

*Dashed line = day of the clock change.

Figure S14: Psychiatric conditions events per day over the Autumn clock changes.

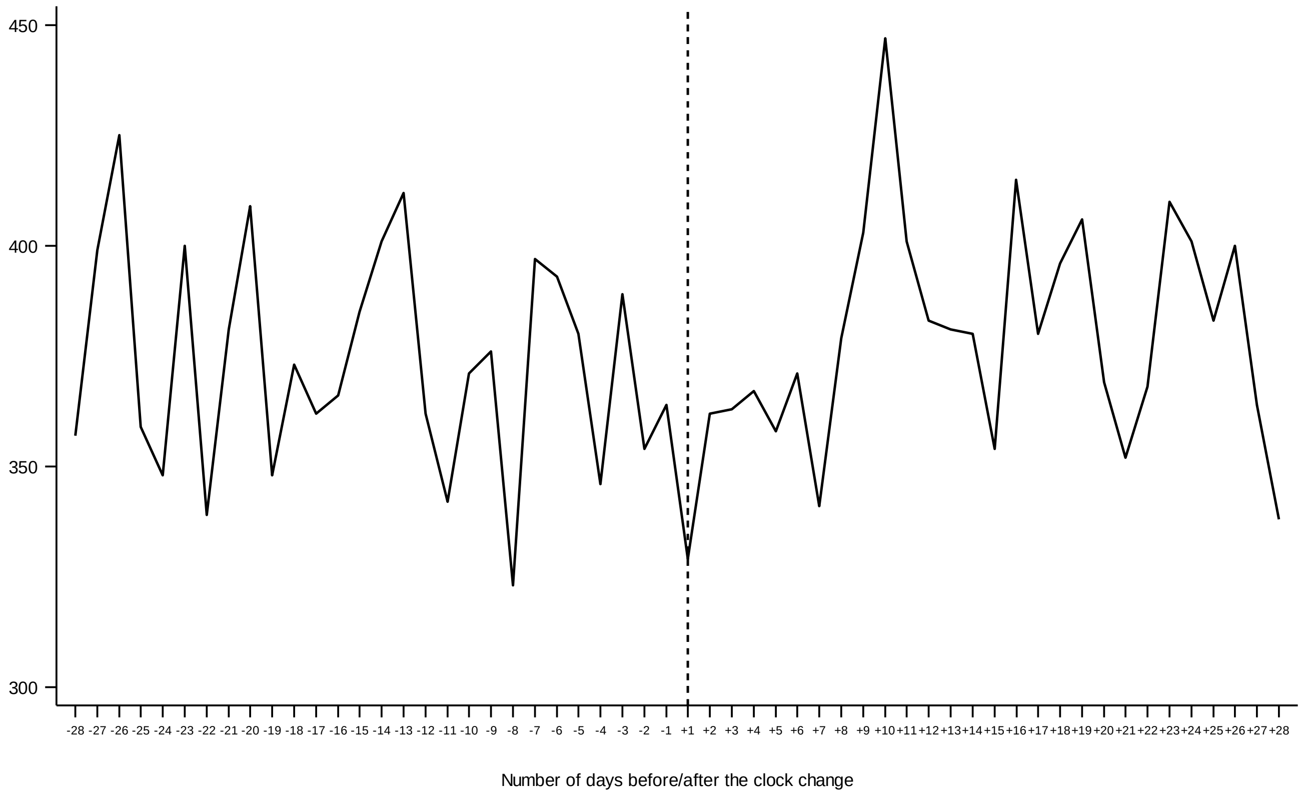

*Dashed line = day of the clock change.

Figure S15: Road traffic injuries events per day over the Autumn clock changes.

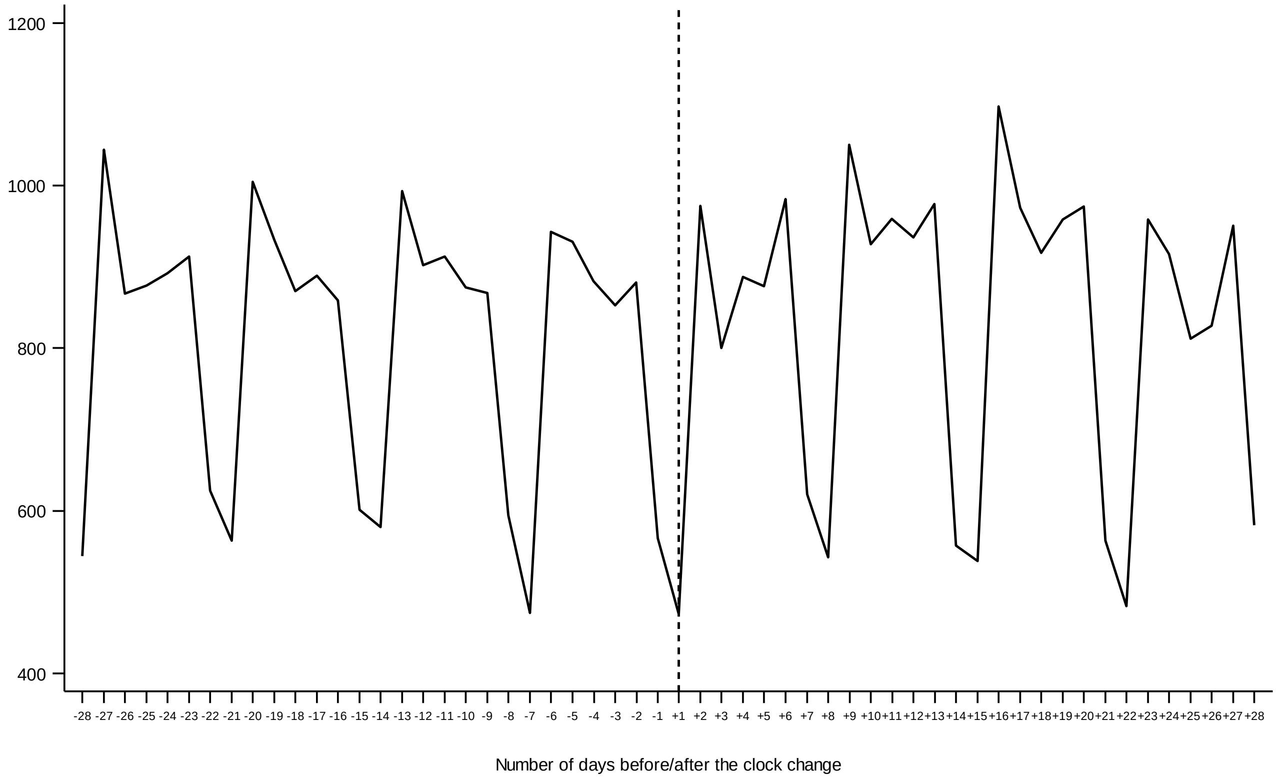

*Dashed line = day of the clock change.

Figure S16: Self-harm events per day over the Autumn clock changes.

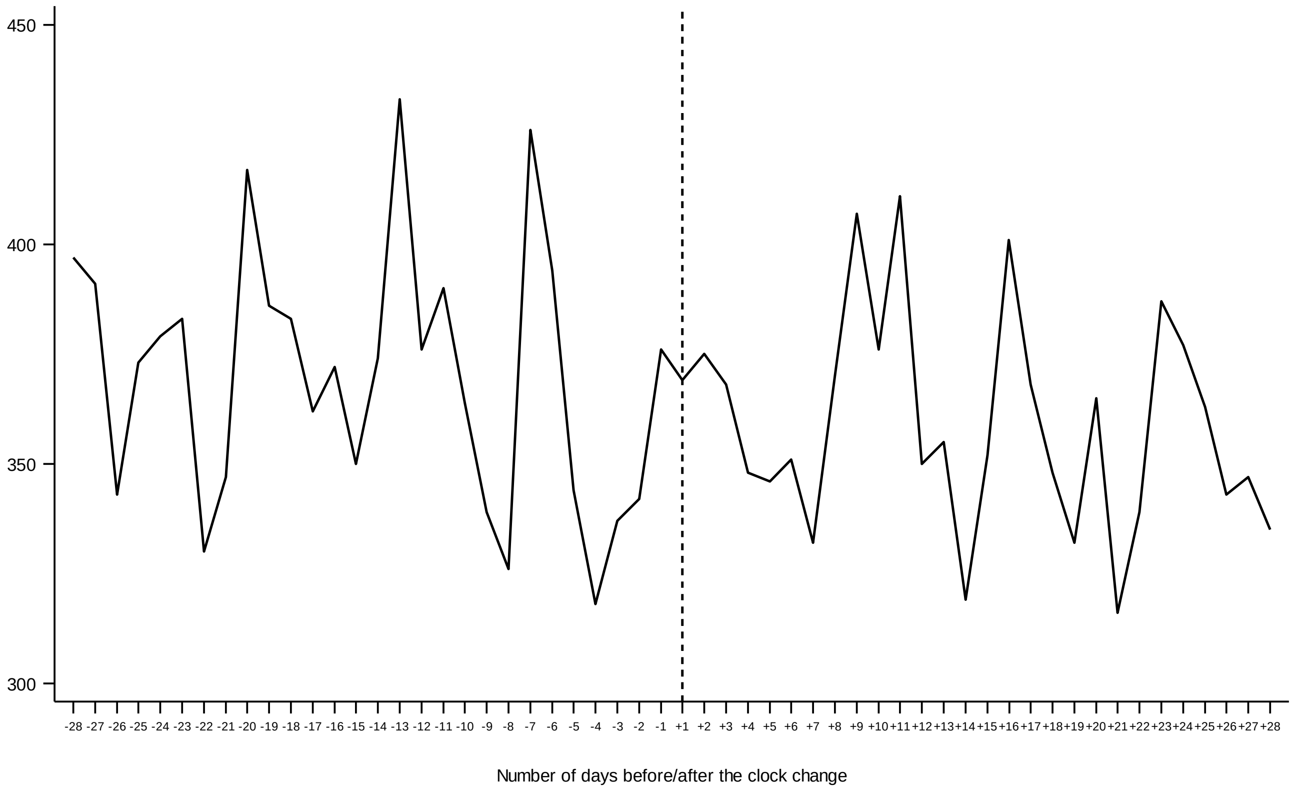

*Dashed line = day of the clock change.

Figure S17: Sleep disorders events per day over the Autumn clock changes.

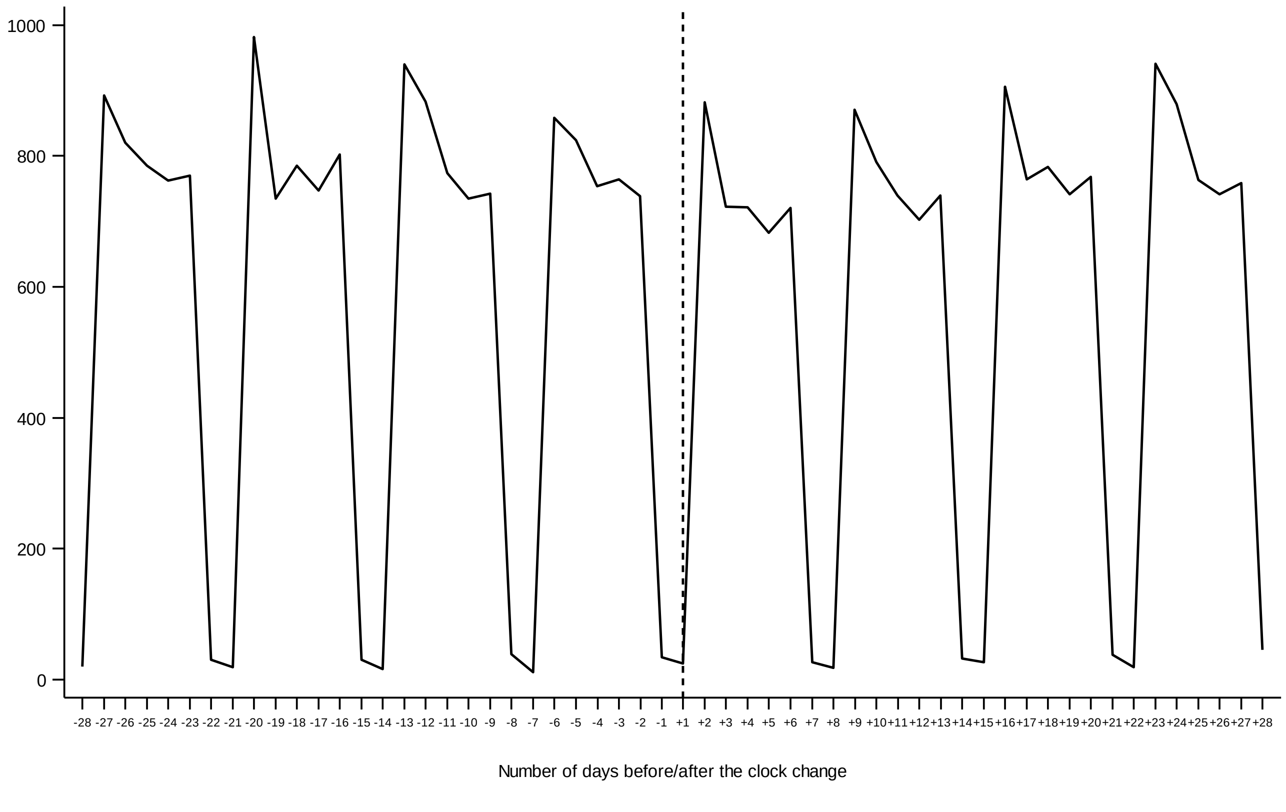

*Dashed line = day of the clock change.

Figure S18: Anxiety events per day over the Spring clock changes versus expected number of events per day.

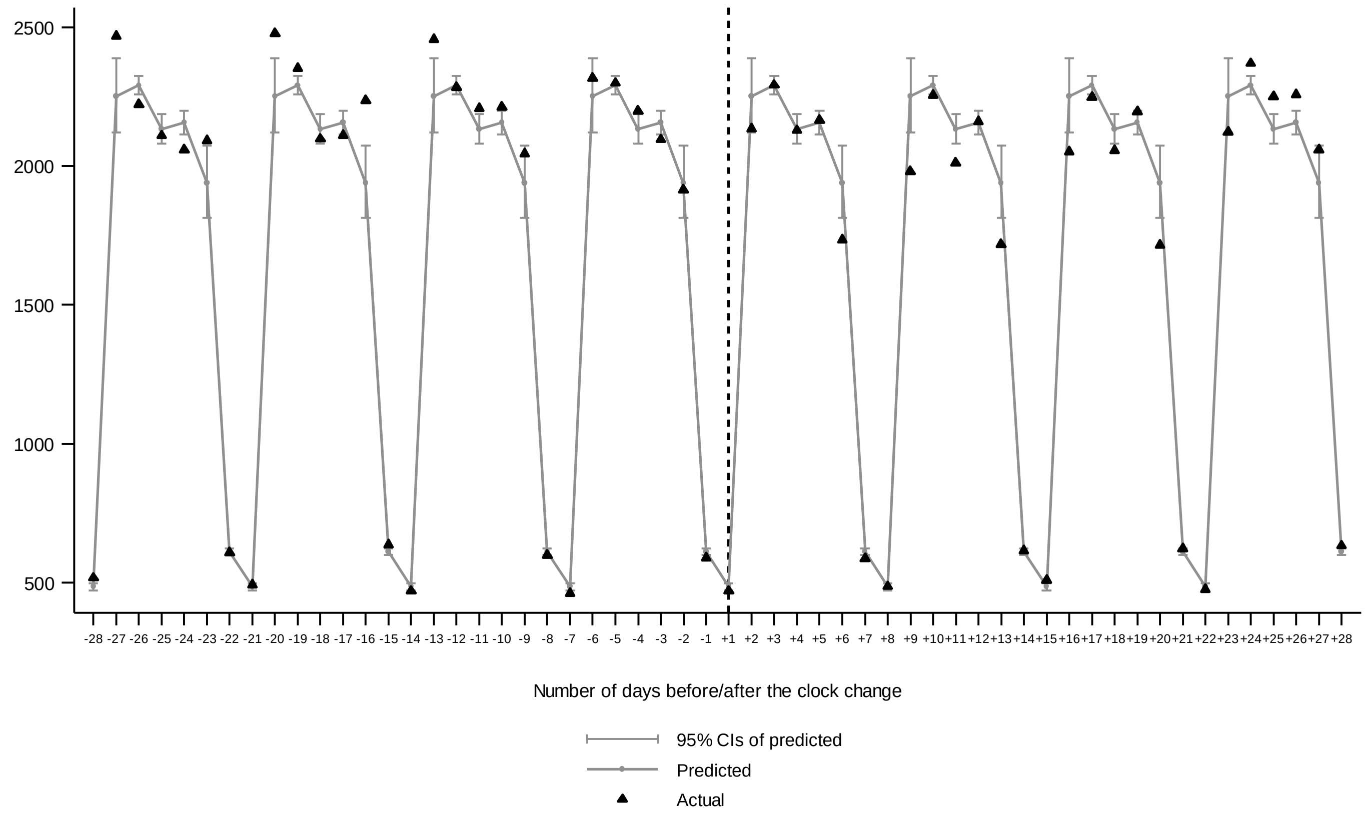

Figure S19: CVD events per day over the Spring clock changes versus expected number of events per day.

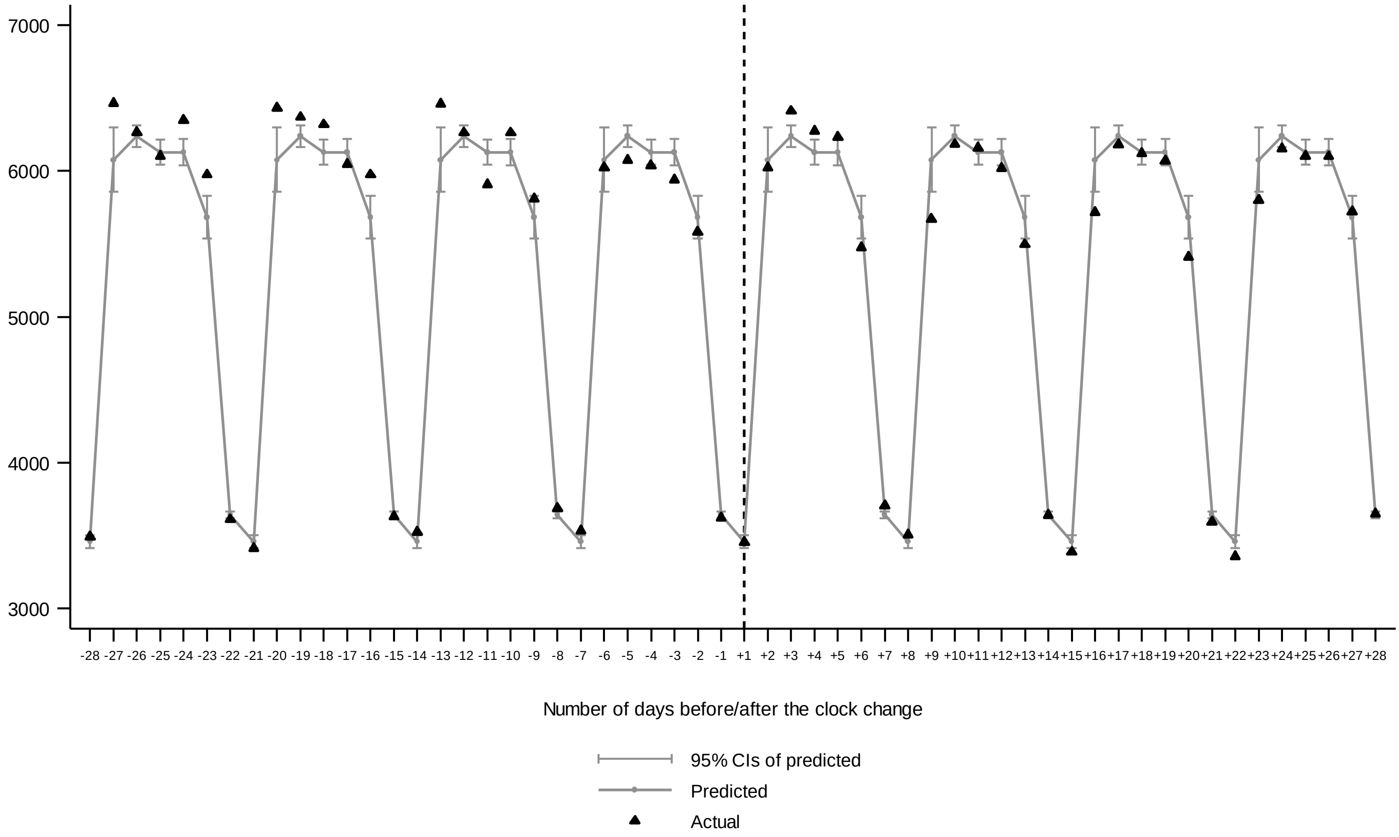

Figure S20: Depression events per day over the Spring clock changes versus expected number of events per day.

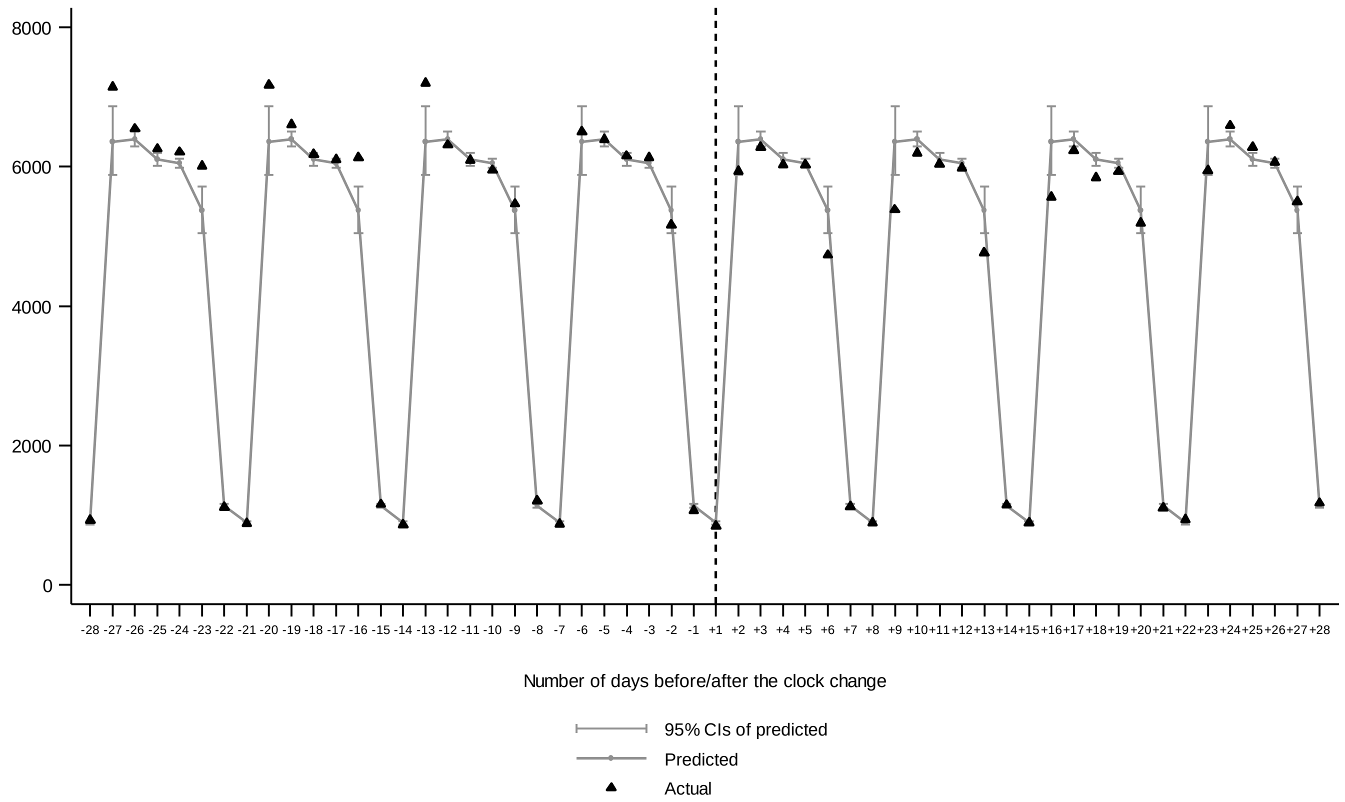

Figure S21: Eat disorder events per day over the Spring clock changes versus expected number of events per day.

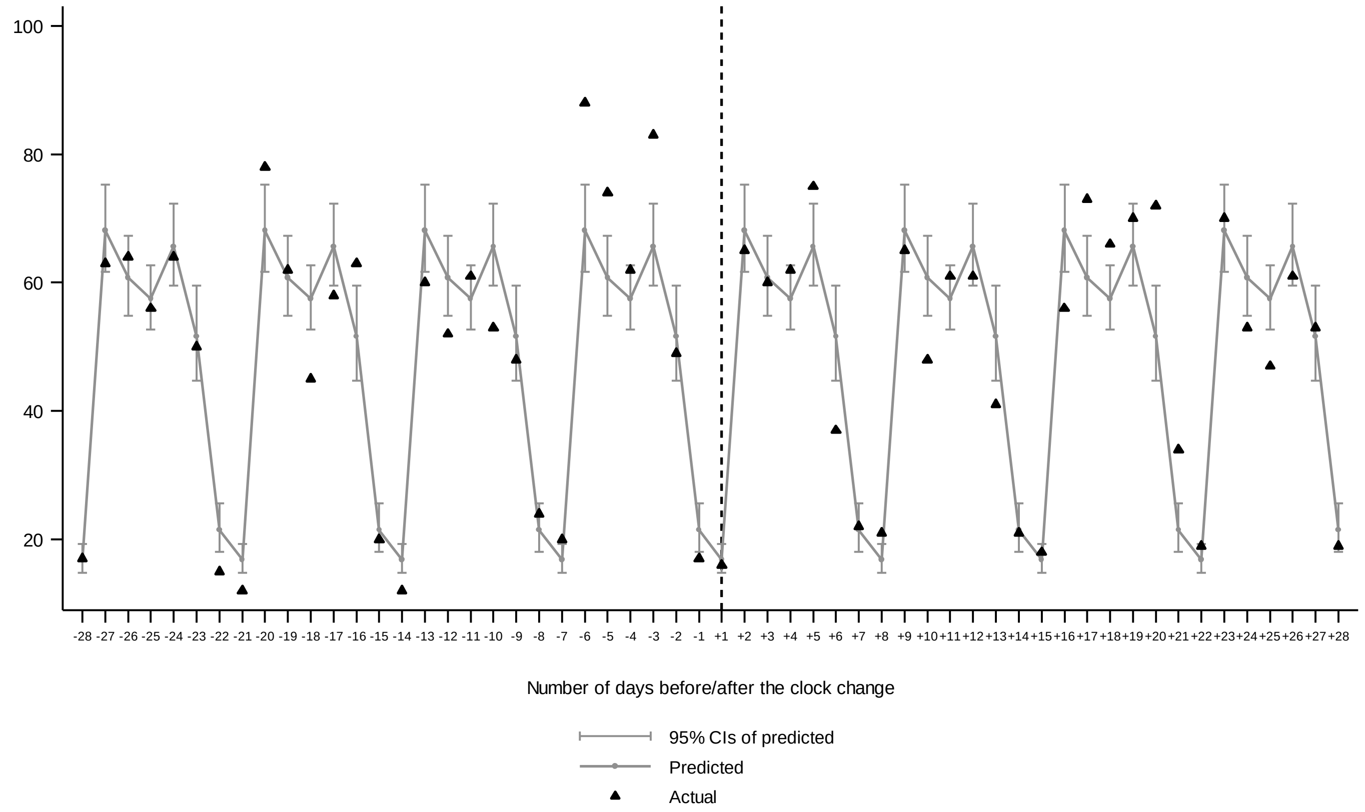

Figure S22: Psychiatric conditions events per day over the Spring clock changes versus expected number of events per day.

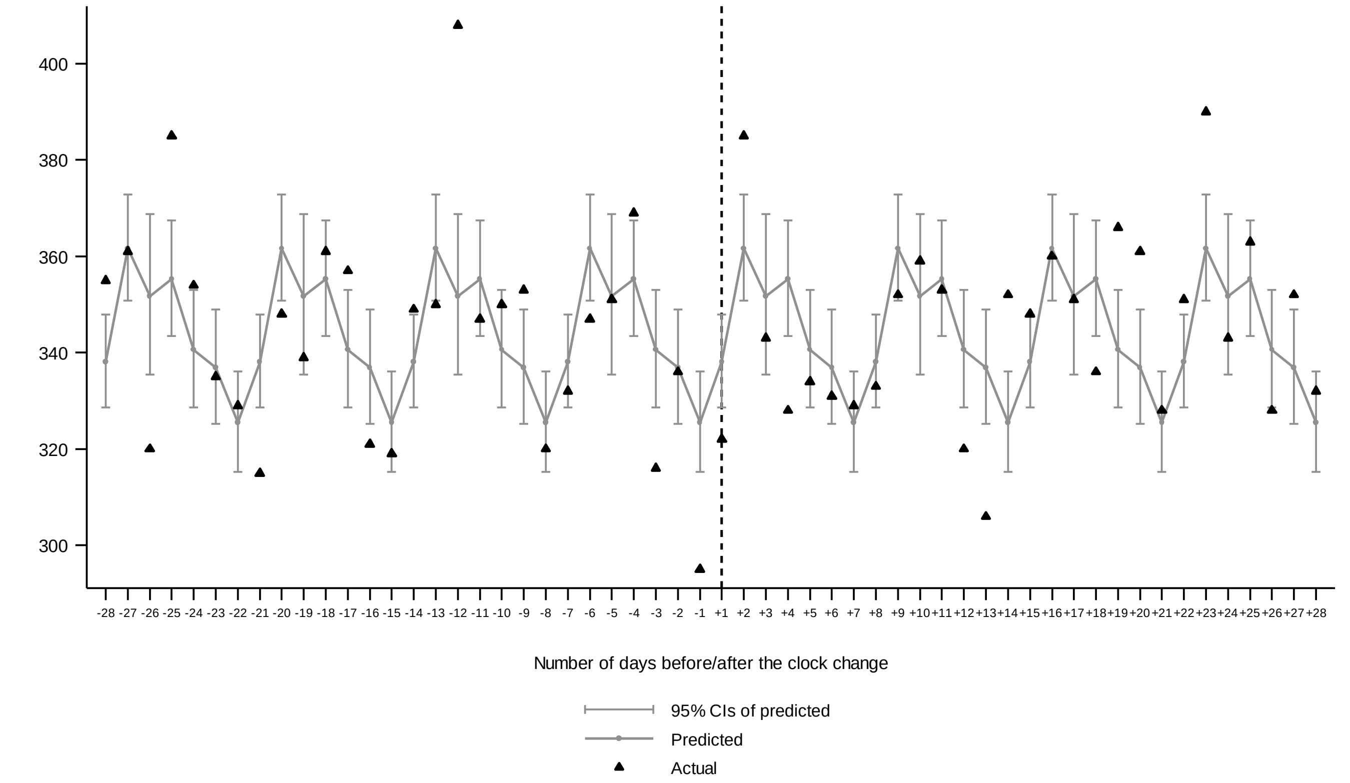

Figure S23: Road traffic injuries events per day over the Spring clock changes versus expected number of events per day.

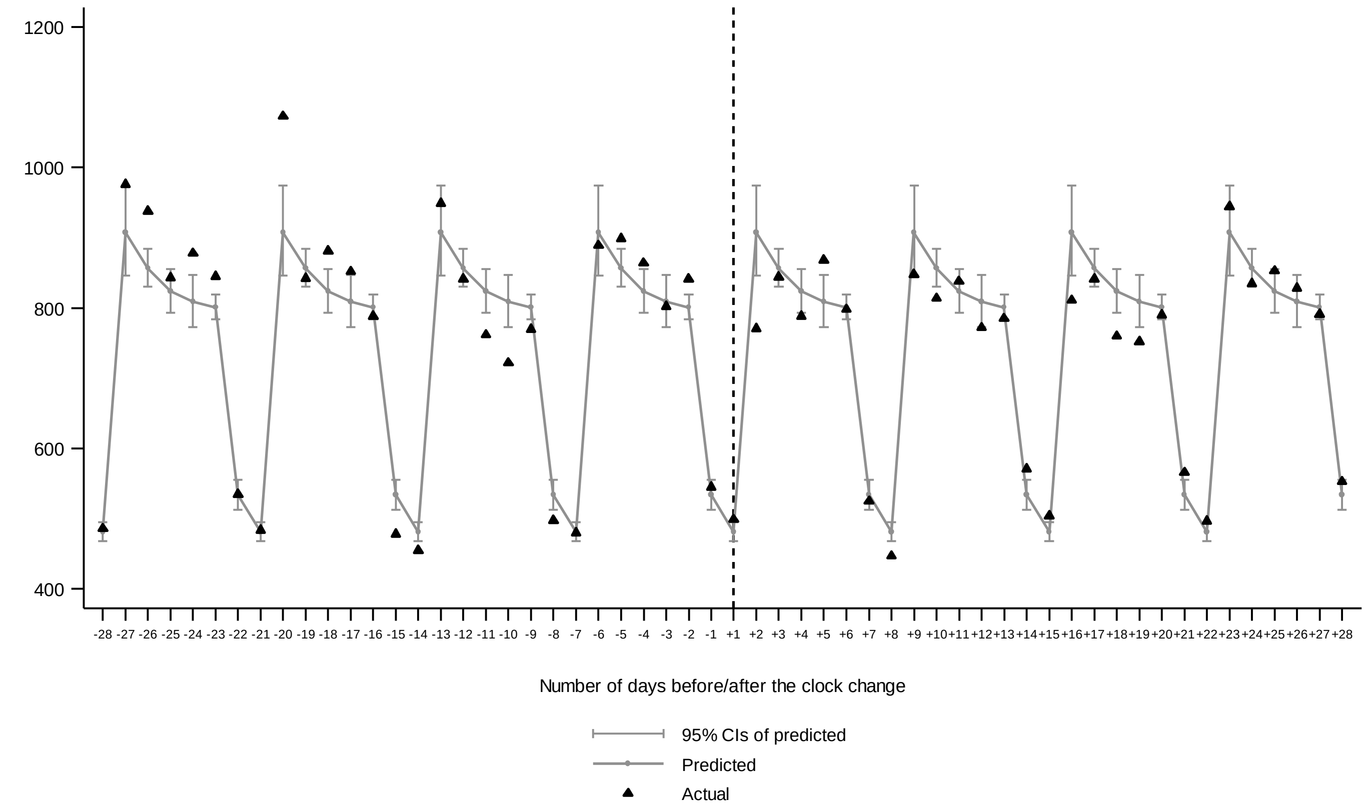

Figure S24: Self-harm events per day over the Spring clock changes versus expected number of events per day.

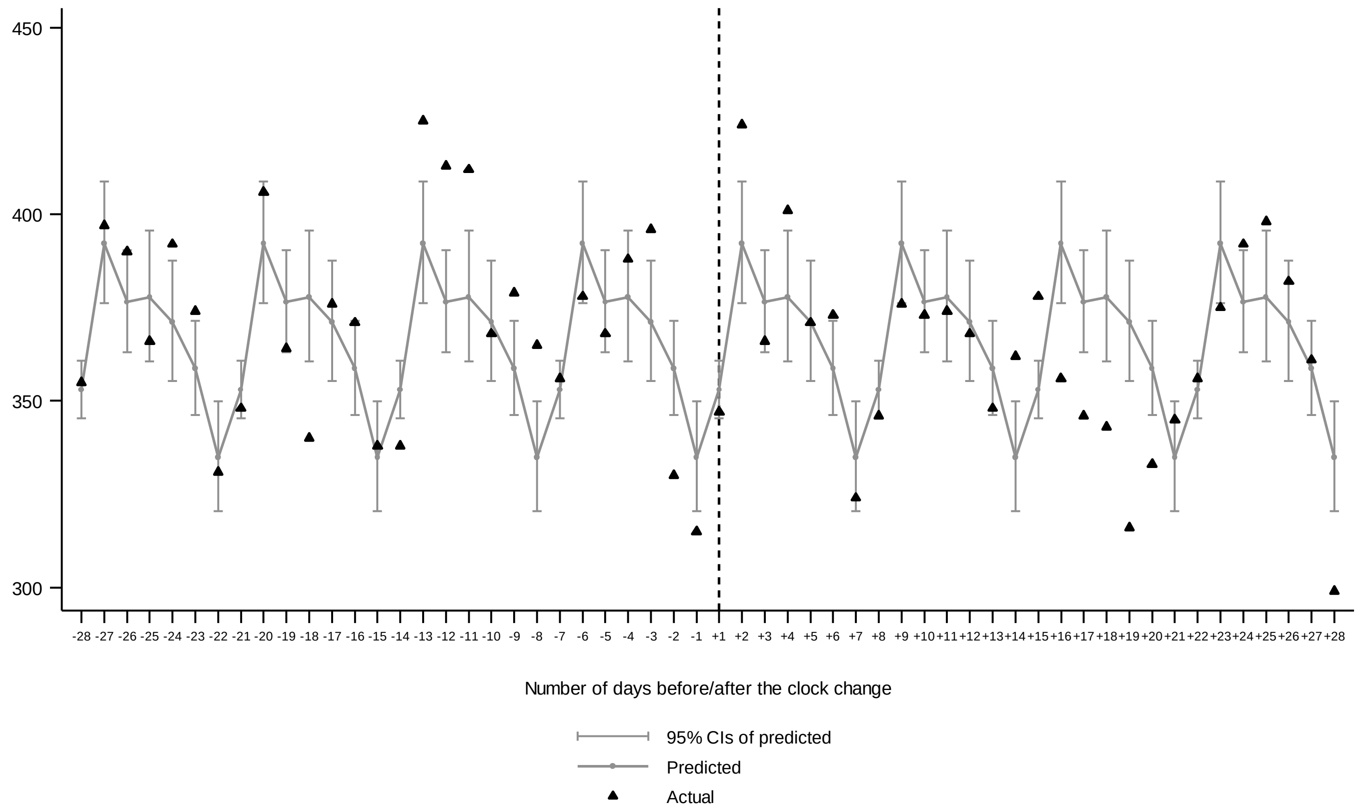

Figure S25: Sleep disorders events per day over the Spring clock changes versus expected number of events per day.

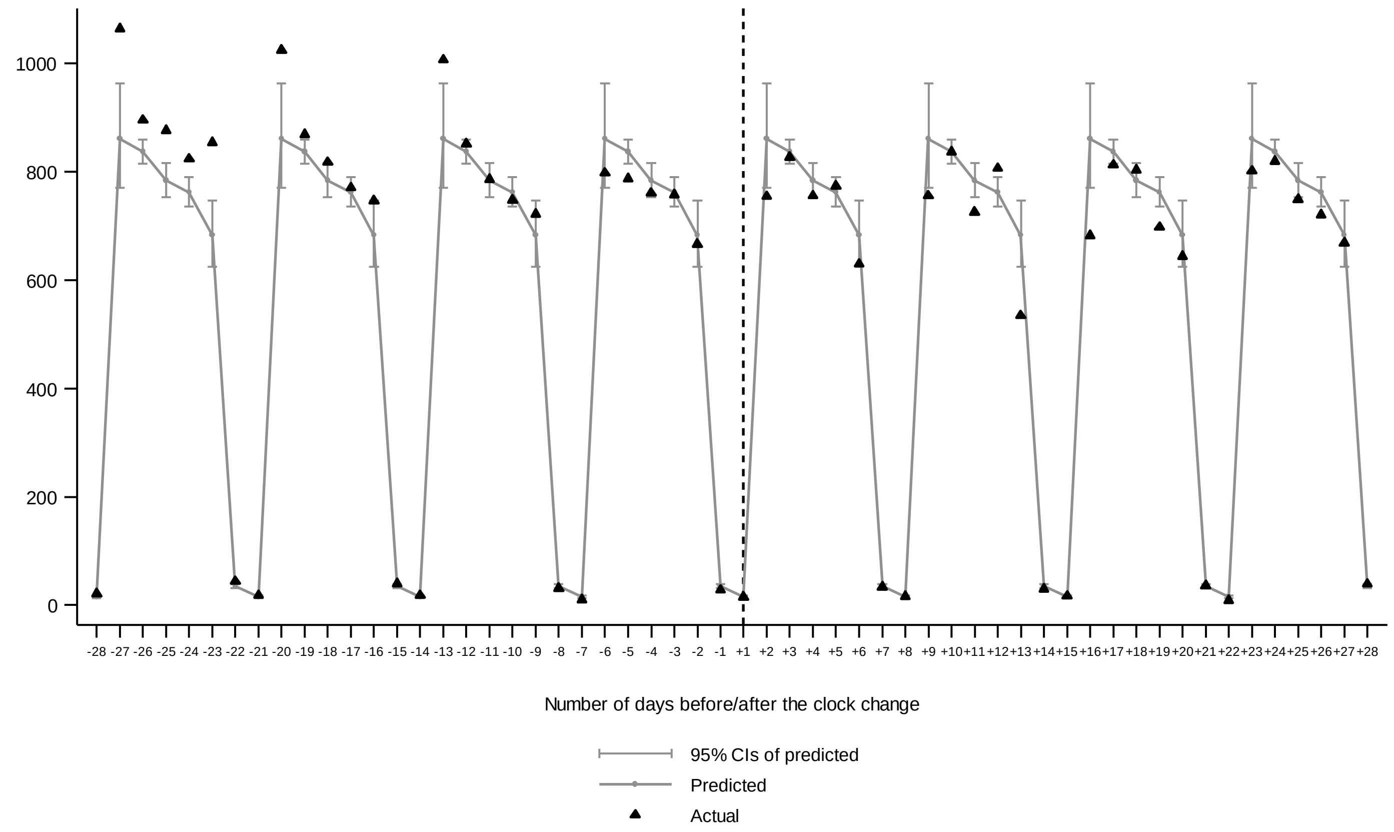

1000
800
600
400
200
0
-28 -27 -26 -25 -24 -23 -22 -21 -20 -19 -18 -17 -16 -15 -14 -13 -12 -11 -10 -9 -8 -7 -6 -5 -4 -3 -2 -1 +1 +2 +3 +4 +5 +6 +7 +8 +9 +10 +11 +12 +13 +14 +15 +16 +17 +18 +19 +20 +21 +22 +23 +24 +25 +26 +27 +28
Number of days before/after the clock change
95% CIs of predicted
Predicted
Actual

Figure S26: Anxiety events per day over the Autumn clock changes versus expected number of events per day.

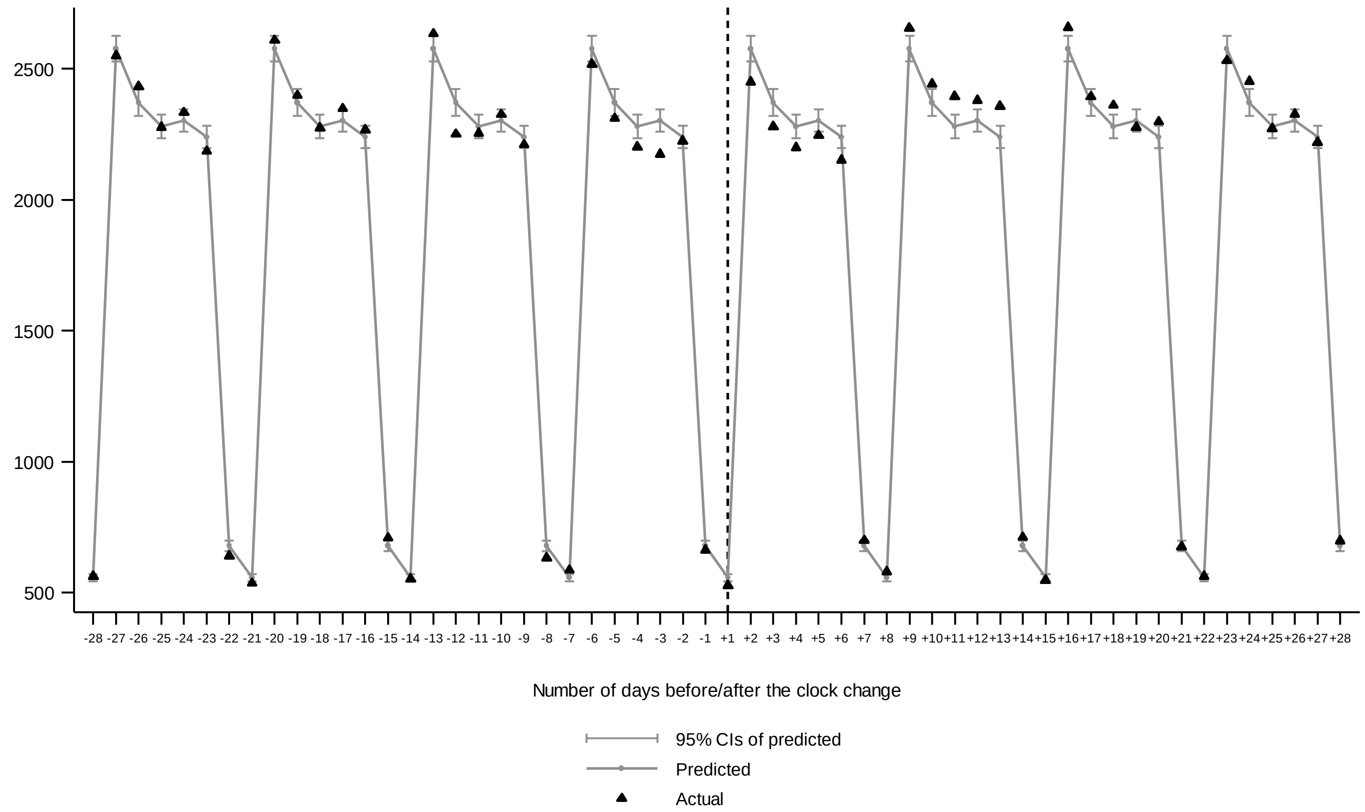

Figure S27: CVD events per day over the Autumn clock changes versus expected number of events per day.

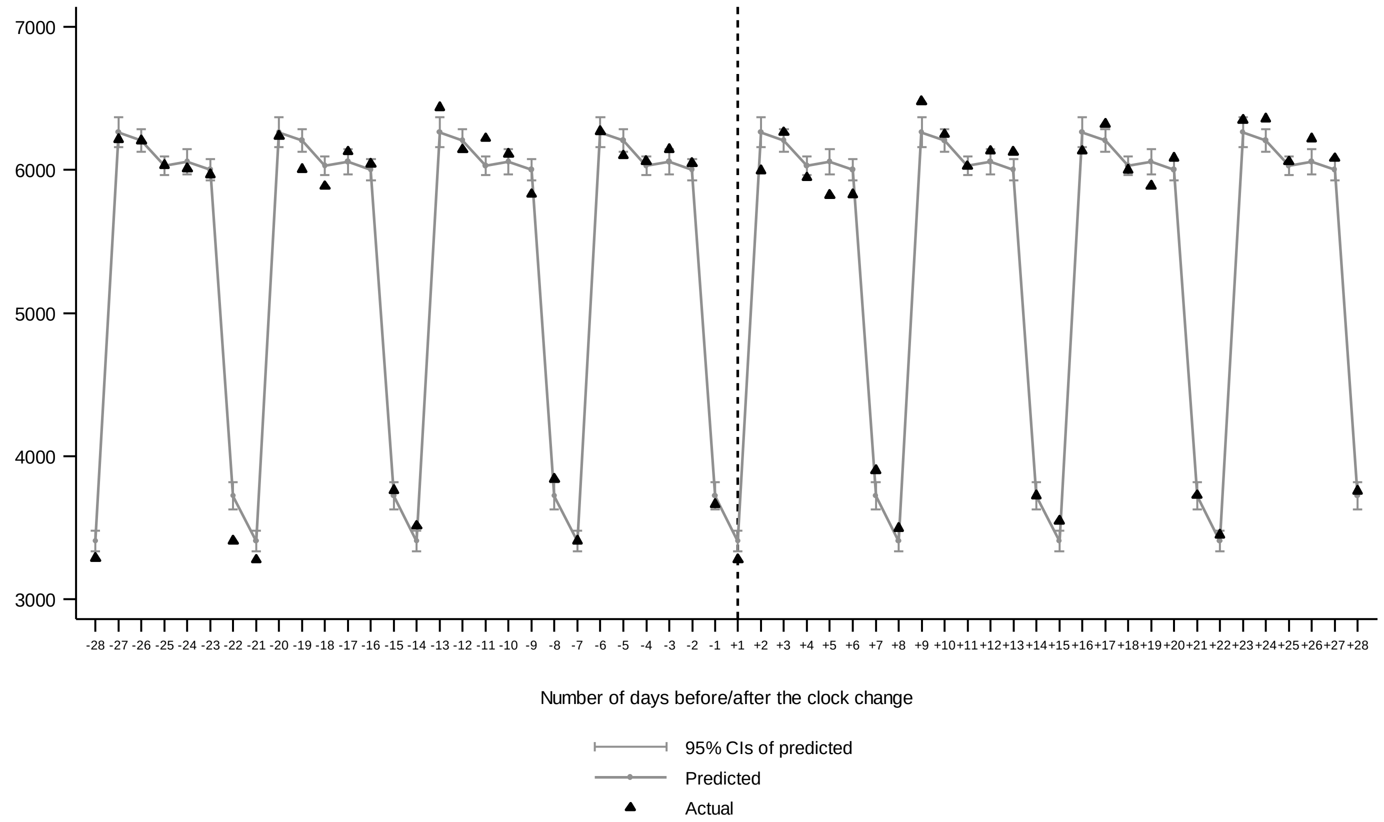

Figure S28: Depression events per day over the Autumn clock changes versus expected number of events per day.

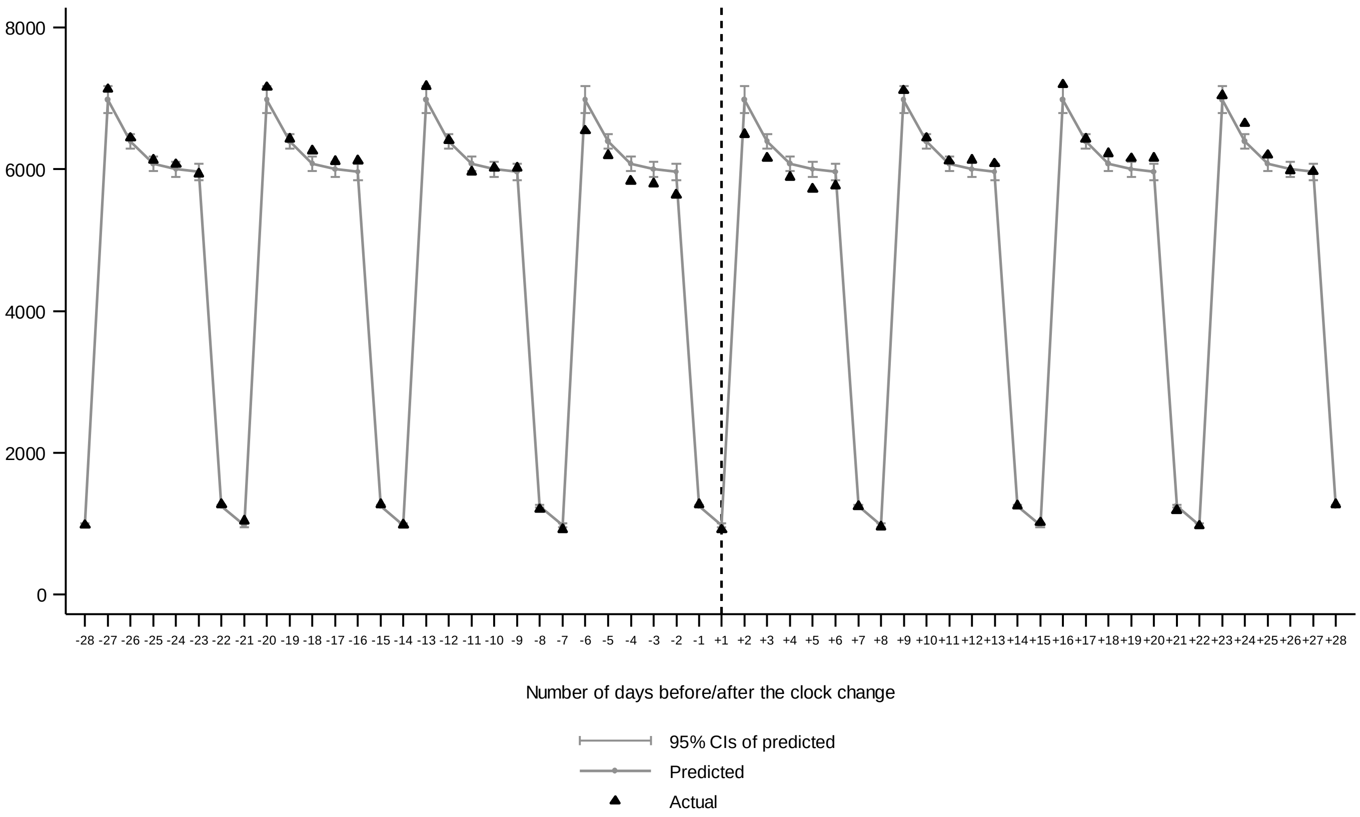

Figure S29: Eating disorder events per day over the Autumn clock changes versus expected number of events per day.

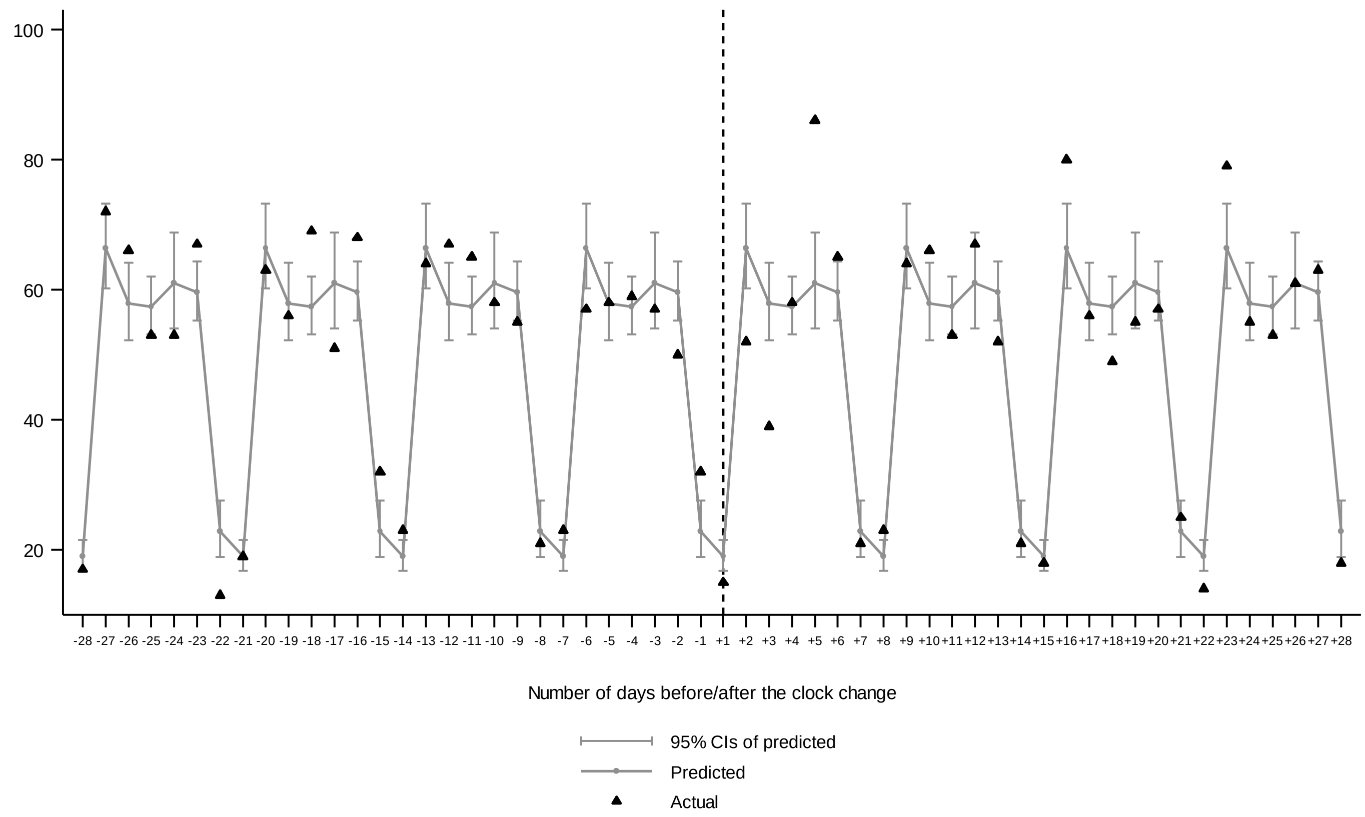

Figure S30: Psychiatric conditions events per day over the Autumn clock changes versus expected number of events per day.

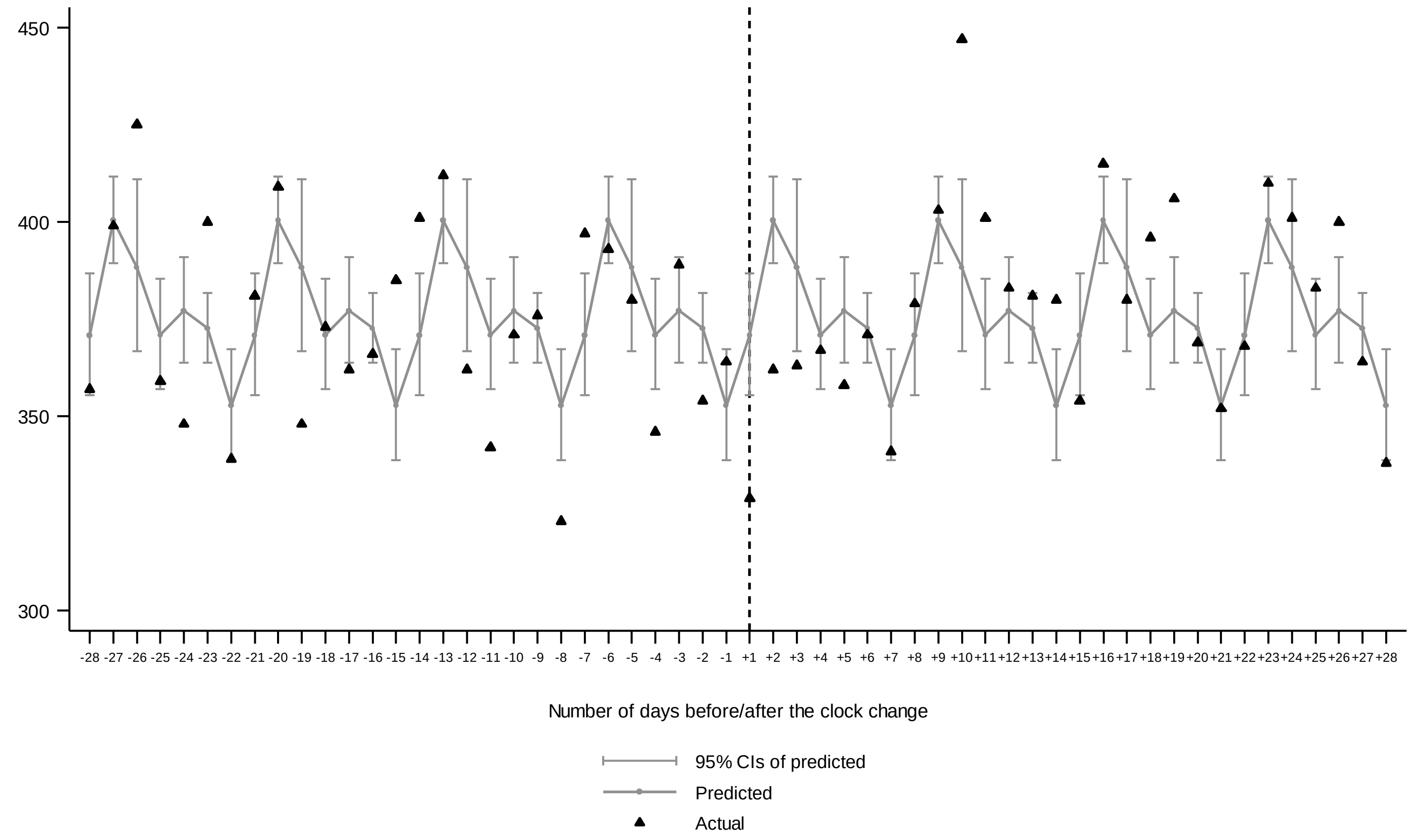

Figure S31: Road traffic injuries events per day over the Autumn clock changes versus expected number of events per day.

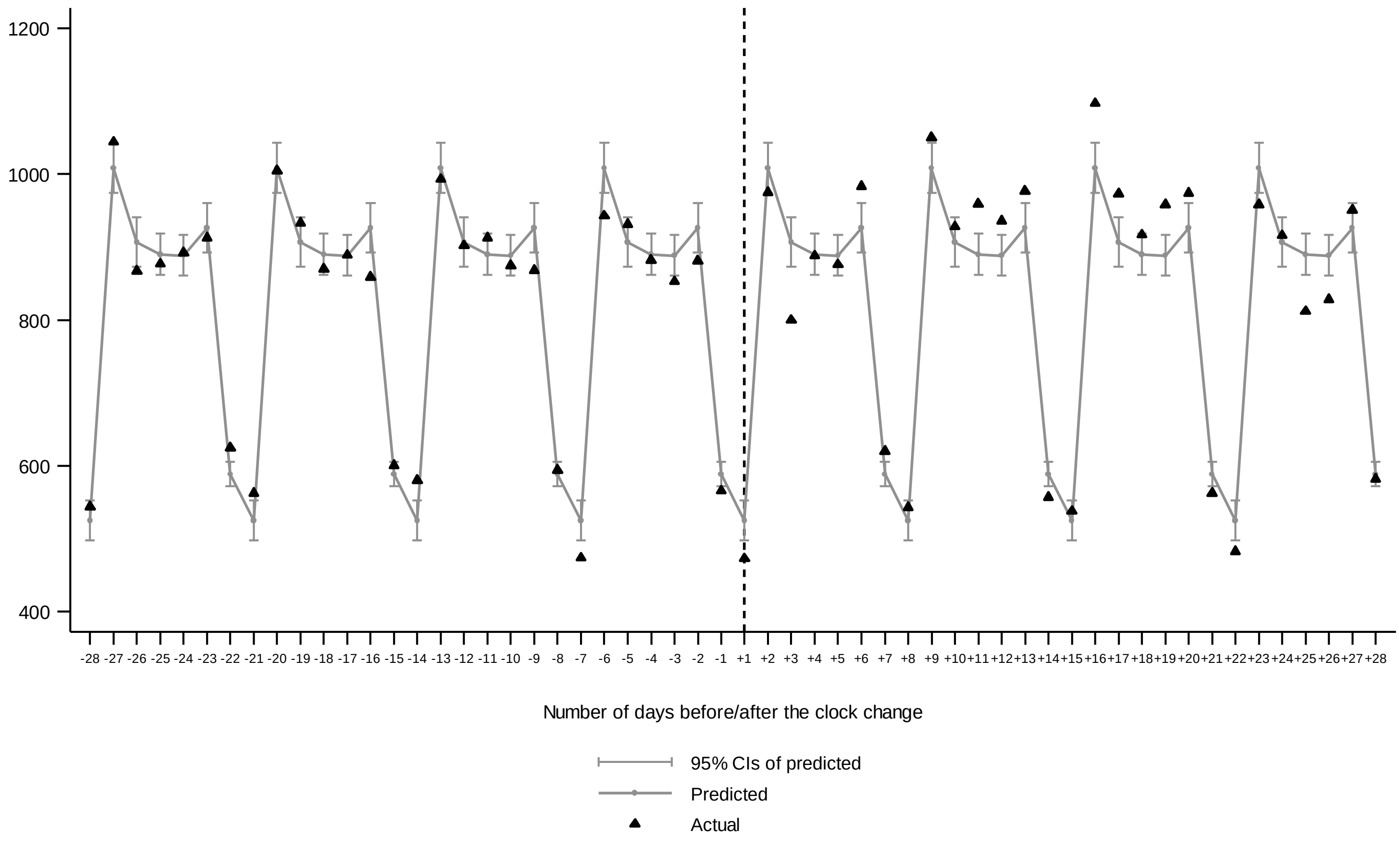

Figure S32: Self-harm events per day over the Autumn clock changes versus expected number of events per day.

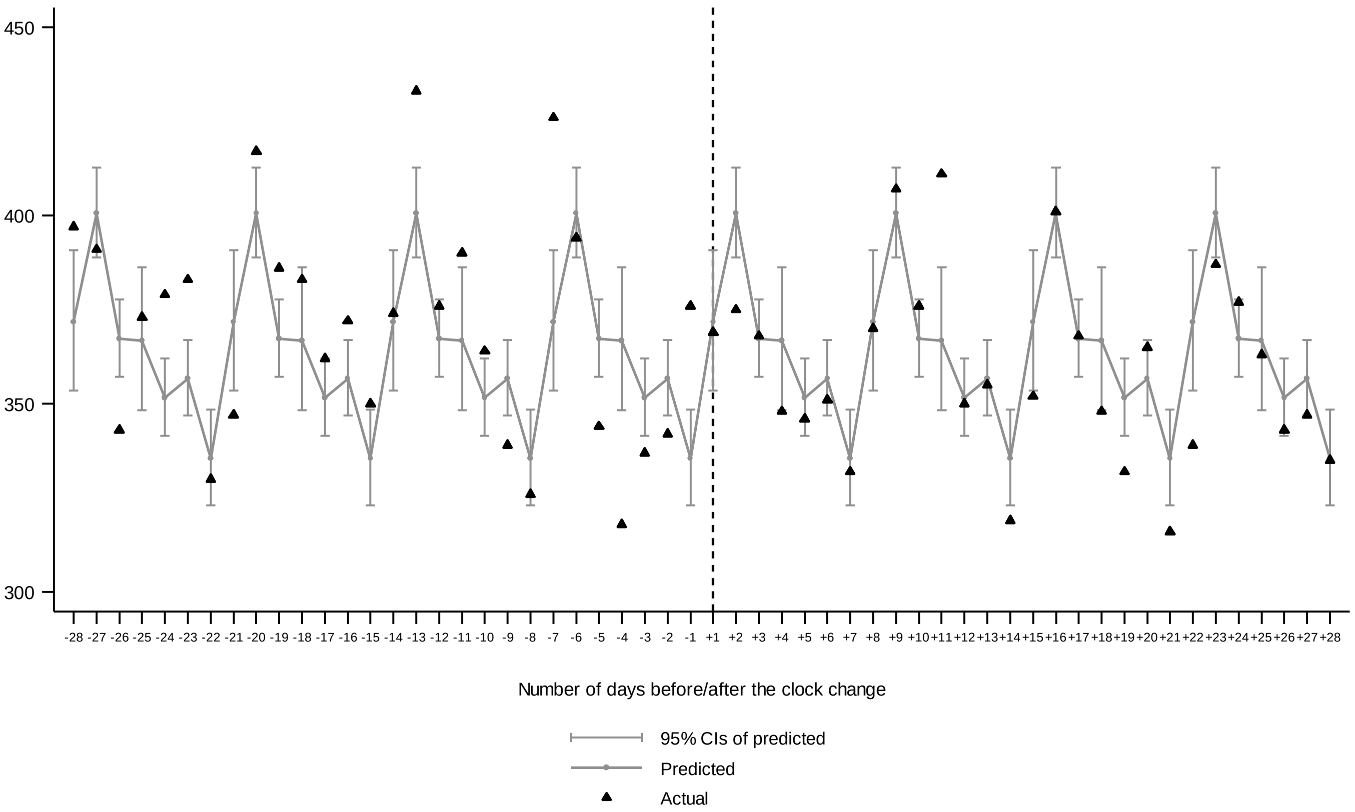

Figure S33: Sleep disorder events per day over the Autumn clock changes versus expected number of events per day.

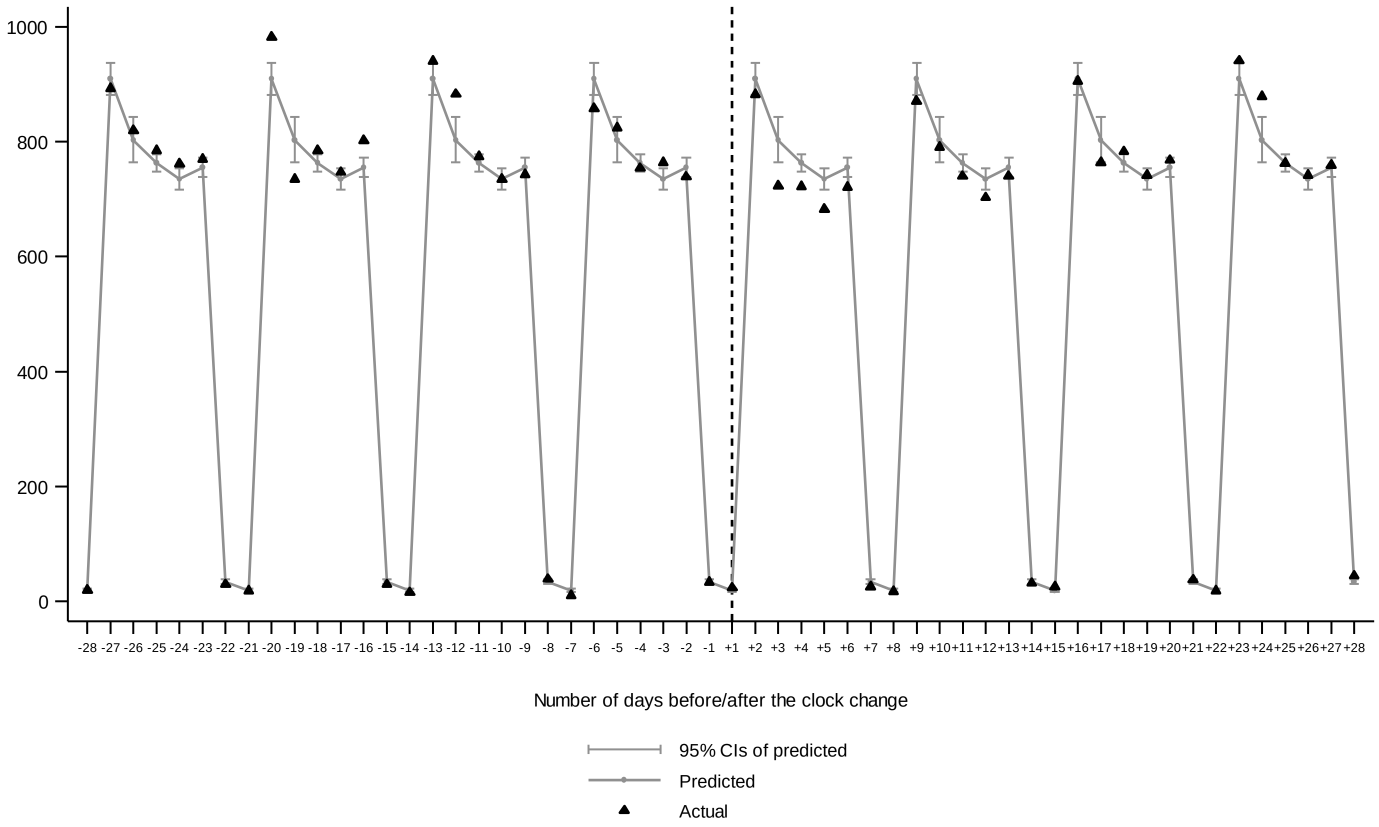

References

1. Mathur R, Bhaskaran K, Chaturvedi N, et al. Completeness and usability of ethnicity data in UK-based primary care and hospital databases. *Journal of Public Health* 2013;36:684-692. doi:10.1093/pubmed/fdt116
